# Four Common Simplifications of Multi-Criteria Decision Analysis do not hold for River Rehabilitation

**DOI:** 10.1371/journal.pone.0150695

**Published:** 2016-03-08

**Authors:** Simone D. Langhans, Judit Lienert

**Affiliations:** Swiss Federal Institute of Aquatic Science and Technology, Eawag, Duebendorf, Switzerland; US Army Engineer Research and Development Center, UNITED STATES

## Abstract

River rehabilitation aims at alleviating negative effects of human impacts such as loss of biodiversity and reduction of ecosystem services. Such interventions entail difficult trade-offs between different ecological and often socio-economic objectives. Multi-Criteria Decision Analysis (MCDA) is a very suitable approach that helps assessing the current ecological state and prioritizing river rehabilitation measures in a standardized way, based on stakeholder or expert preferences. Applications of MCDA in river rehabilitation projects are often simplified, i.e. using a limited number of objectives and indicators, assuming linear value functions, aggregating individual indicator assessments additively, and/or assuming risk neutrality of experts. Here, we demonstrate an implementation of MCDA expert preference assessments to river rehabilitation and provide ample material for other applications. To test whether the above simplifications reflect common expert opinion, we carried out very detailed interviews with five river ecologists and a hydraulic engineer. We defined essential objectives and measurable quality indicators (attributes), elicited the experts´ preferences for objectives on a standardized scale (value functions) and their risk attitude, and identified suitable aggregation methods. The experts recommended an extensive objectives hierarchy including between 54 and 93 essential objectives and between 37 to 61 essential attributes. For 81% of these, they defined non-linear value functions and in 76% recommended multiplicative aggregation. The experts were risk averse or risk prone (but never risk neutral), depending on the current ecological state of the river, and the experts´ personal importance of objectives. We conclude that the four commonly applied simplifications clearly do not reflect the opinion of river rehabilitation experts. The optimal level of model complexity, however, remains highly case-study specific depending on data and resource availability, the context, and the complexity of the decision problem.

## 1. Introduction

Due to human impact, freshwaters are among the most seriously threatened and modified environments on the planet [1]. Habitat destruction and fragmentation, physical and chemical alterations, and the introduction of exotic species or overexploitation of freshwater resources, have severely altered the ecology of freshwaters [2]. During the next decades, the pressure on freshwater ecosystems is expected to increase, driven by the combined effects of population growth and climate change [3].

In response to the poor conservation status of freshwater biodiversity and the increasing loss of services that humans receive from freshwater ecosystems, river rehabilitation is a global priority [3, 4]. The success of a rehabilitation that aims at improving the overall ecological state of a degraded river system depends on concurrently achieving quality targets for biological, chemical, and physical elements. The valuation of these elements, however, is case-specific and therefore difficult to base on empirical data, which is often sketchy. To satisfy this complexity, river rehabilitation often relies on the knowledge of experts, who deal with the assessment of and the trade-offs between the quality elements. Additionally, socio-economic considerations might be included [5], in which case the assessment also depends on the preferences of stakeholders or the public. However, socio-economic preference elicitation from stakeholders is not the focus of this paper; for a more extensive discussion see [6].

Expert knowledge has gained momentum as a source of information for decision making, particularly in contexts where empirical information is sparse or unobtainable [7–9]. In such situations, previous learning and experience provide valuable insight into the behavior of environmental systems [10]. Multi-Criteria Decision Analysis (MCDA; [11–14]) is naturally suited to incorporate expert knowledge through value functions. These are expert preferences for objectives (see explanation in Table 1) on a standardized scale. MCDA helps to structure and facilitate complex projects with multi-stakeholder involvement, which is typical for river rehabilitation. During the last two decades, MCDA has been increasingly adopted to support decision making in the environmental field [15, 16]. However, in river rehabilitation it is still rarely applied. For example, a recent meta-analysis found that of 60 river rehabilitation projects 26 would benefit from MCDA, but only four actually used it [17].

**Table 1:** Glossary. Terminologies used in Multi-Criteria Decision Analysis (MCDA; see also Table 1 in [20]).

MCDA terminology	Explanation
Multi-Criteria Decision Analysis (MCDA)	"Thus we use the expression MCDA as an umbrella term to describe a collection of formal approaches which seek to take explicit account of multiple criteria in helping individuals or groups explore decisions that matter." ([11], p. 2)
Multi-Attribute Value Theory (MAVT)	“Value measurement models (e.g. [13]) in which numerical scores are constructed, in order to represent the degree to which one decision option may be preferred to another. Such scores are developed initially for each individual criterion, and are then synthesized in order to effect aggregation into higher level preference models.” ([11], p. 9)
Multi-Attribute Utility Theory (MAUT)	“Utility theory can be viewed as an extension of value measurement, relating to the use of probabilities and expectations to deal with uncertainty.” ([11], p. 95; also see [13]). “… we want to model a decision maker’s preferences in case he faces risky alternatives.” ([12], p. 235).
Main objective	Overall ecological target or goal to be met after the rehabilitation, defined by the experts. Is placed at the highest level, if objectives are organized into an objectives hierarchy.
Sub-objective	“Each sub-objective covers an important aspect of the objective at the higher level; all sub-objectives associated with the same higher level objective cover all relevant aspects.” ([20], Table 1).
Attribute	Indicator that assesses how well an endpoint is reached when a rehabilitation measure is implemented. Each sub-objective at the lowest level is associated with at least one attribute [12].
Value function	“Description of the degree of fulfilment of the corresponding objective as a function of associated attributes on a common scale from 0 to 1.” ([20], Table 1).
Utility function	As value function, but considering the expert’s risk attitude when confronted with uncertain (risky) outcomes.
Bisection method to elicit value function	One asks, e.g., for an improvement from the worst-possible state (value = 0) to an approximate medium state (value = 0.5) that is equal in value to an improvement from this medium to the best-possible state (value = 1). We defined "the good state" to be the condition of the objective which could be achieved when implementing the most appropriate rehabilitation measure under given restrictions, such as existing towns, roads, and agricultural land use. (further explanations see [12]; example interview see S7 File).
Swing method to elicit scaling constants (weights) for Construction of value function	Hereby, all objectives are set to their worst level, then “swung” one after the other to their best level, resulting in a ranking of the importance of objectives, according to the expert’s opinion. Then scoring (weighting) is carried out, whereby the combination of the most important objective being on its best level, and all others on their worst, receives 100 points, and all objectives being on their worst level receives 0 points. Remaining combinations receive scores in-between (e.g. second-important objective on its best level, all others on worst); scores reflect value differences between 0 and 100 points. Scores are normalized to [0, 1], (see [11, 12]).
Reversed-Swing method to elicit scaling constants (weights)	Same as Swing method, but all objectives are set to their best level, and then “swung” one after the other to their worst level [18].
Direct rating method to elicit scaling constants (weights)	Ranking of objectives according to their importance and assigning direct values to each objective compared to the importance of the other objectives; method not generally recommended [12], but see [31].

In this paper, we focus on Multi-Attribute Value and Utility Theory (MAVT/MAUT), two methods within the MCDA family that are grounded on axioms of rational choice (e.g. [12, 13]). These methods can easily deal with a large number of alternatives (Table 1) and new or compromise alternatives can be included at any stage of the decision process [18]. This has been found to be important in the case of conflicting interests in river rehabilitations in Switzerland [19]. MAVT and MAUT can also consider uncertainty of predictions inherent in environmental data, and MAUT includes the risk attitude of the decision makers, i.e., their preferences in face of uncertain outcomes (e.g. [6, 12, 13]. MAVT/MAUT allow assessing different objectives targeted by river rehabilitation, e.g. reaching a good physical, chemical or biological state of the river, and aggregating these assessments into an overall score. A value function represents the degree of fulfilment of the overall objective on a scale from zero to unity as a function of scientifically measurable system properties, the attributes. To facilitate the construction of such a value function, the overall objective is broken down into objectives at lower hierarchical levels. The value function for the overall objective is constructed by formulating individual value functions for each lowest-level objective, as a function of a number of attributes, and aggregating the values to the next-higher levels. Value functions representing expert preferences for ecological objectives and their aggregation may be elicited from scratch following a pre-defined, rigorous protocol to avoid biases; as demonstrated in this paper. To decrease elicitations costs, value functions can also be translated from existing assessment protocols. Thereby, the assessments of the individual physical, chemical, or biological elements defined in the protocols, be it in a qualitative or quantitative way, are standardized onto a common scale from 0 to 1 (please see [20, 21] for detailed information on this procedure). If an outcome of a decision, such as the consequences of implementing a rehabilitation measure is uncertain, the experts’ preferences can change. In this case, risk attitudes have to be elicited additionally to value functions, to transform the values to utilities.

Experts should be selected in a way to cover all aspects of the respective MCDA. If stakeholders are included, this can be done with the help of a stakeholder or social network analysis [22]. For river rehabilitation that aims at improving the ecological state, different experts concerning the functioning of river ecosystems such as ecologists, hydraulic engineers, water chemists, or physicists should be involved.

MCDA-applications often use a relatively pragmatic approach to elicit expert knowledge, because quantifying preferences is a laborious and time-consuming process. The most frequent simplification in on-the-ground river rehabilitation is to include only a limited number of objectives and attributes. We found a restriction to some five to ten objectives or attributes in MCDA-approaches [17, 19, 23–26] and non-MCDA approaches that strived to set standards for successful river rehabilitation [27].

Another simplification is assuming that all value functions are linear [25], thus circumventing their laborious elicitation [19, 24]. Although intuitively compelling, linear value functions may not reflect the preferences of decision makers. To give an example, for an attribute ranging from 0 to 100 units (e.g. diversity index), linearity implies that an improvement from 0 to 50 units is equally valuable as an improvement from 50 to 100 units. In reality, experts may value the improvement of a heavily degraded river higher than of one which is already in a good condition (or vice versa) resulting in non-linear value functions.

Additivity (i.e. weighted averaging) is often assumed when aggregating values of different objectives [17, 19, 24], despite its use being problematic. Most importantly, this is due to the fact that additivity entails the assumption of preferential independence of objectives, i.e. that preferences for the level of one objective can be specified independently of the levels of other objectives [12, 13]. The additive model also implies that a low value of one objective can be compensated by a high value of another one. As an example: if fish are in a very bad state, the additive model implies that the bad state of fish can be fully compensated by benthic organisms being in a very good state (given that both receive equal weights). The result is that at the next higher level of the objectives hierarchy, biodiversity will be in a medium state when the two sub-objectives fish and benthic organisms are aggregated. However, experts may not want to allow for any compensation or for only some, which is possible for example by using minimum or multiplicative aggregation, respectively (S4 File).

Finally in the face of uncertain (rehabilitation) outcomes, it is necessary to consider the risk attitude of decision makers. The risk attitude is needed to convert value functions into utility functions [28]. When eliciting utility functions, decision makers have to express preferences for probabilistic outcomes, typically presented as lotteries which is more demanding than asking for value functions [12]. Thus, in many applications, risk neutrality is assumed with the implication that the shape of the value function is identical to that of the utility function. Giving again an example: risk neutrality implies that a decision maker is indifferent between two options A and B. In option A, the ecological state of the river is 50% for sure. In option B there is uncertainty, and there is a 50% probability that the river is in a very bad state, and a 50% probability that it is in a very good state. In real-world applications, many decision makers might prefer the sure outcome A to the uncertain one B, implying risk aversion [29]. This risk attitude, be it risk prone or risk averse, depends on an individual person’s preferences. Mathematically, it implies a convex or concave shift in the shape of the value function, respectively [30].

The main aim of this study was to investigate whether the simplifications discussed above, i.e. concerning the number of objectives and attributes, the shape of the value functions, the choice of the aggregation method, and the assumption of risk neutrality reflect expert opinion, specifically for the evaluation of the success of ecological river rehabilitation. For this, we base our study on intensive and very detailed face-to-face interviews with river rehabilitation experts. We hypothesized that 1) if given the choice, experts prefer to include a broad range of objectives and attributes, 2) most of the elicited value functions will be non-linear, 3) experts prefer other aggregation methods than additivity, and 4) the risk attitude of the experts will vary. Additionally, we demonstrate in detail, how an expert-interview-based MCDA-approach can be implemented and provide ample (supplementary) material for other applications. Based on our findings, we aim to formulate recommendations for the elicitation of expert knowledge, particularly in the context of river rehabilitation.

## 2. Material and Methods

### 2.1 Interview procedure

We started the knowledge elicitation process with a fish ecologist (hereafter called Fish-expert), a biologist/physicist (BioPhys-expert) and a river ecologist (BioA; Table 2). These experts suggested additional experts needed to cover the whole range of objectives that are usually targeted in small-scale river rehabilitation: two river ecologists with different expertise (BioB, and BioC) and a hydraulic engineer (Phys). This method of expert identification is often referred to as snowball sampling [32]. All experts had proven expertise in river rehabilitation.

**Table 2:** Summary of expert information. Expert nickname (Exp.), main research fields of expert and approx. years of experience (Exper.), the objectives for which each expert provided information (Elicitation), the method to elicit weights (Weight), and whether the expert’s risk attitude was elicited (Risk).

Exp.	Research fields	Exper.	Elicitation	Weight	Risk
Fish	fish ecology, radio telemetry, rehabilitation, ecology	35 y	fish, physico-chemical water quality, natural discharge regime	swing, reversed-swing	no
BioA	aquatic-terrestrial interactions, ecological river assessment, river rehabilitation	20 y	shoreline fauna, benthic organisms	swing	yes
BioB	ecological stream assessment, multi- criteria decision support, floodplain ecology	10 y	benthic organisms, floodplain vegetation, ecosystem stability	swing	yes
BioC	ecohydrology, freshwater biology, biodiversity, river and floodplain ecology	25 y	ecosystem stability	swing	no
BioPhys	effects of flow and temperature on carbon fluxes, biogeochemical cycles, ecosystem services	10 y	benthic organisms, floodplain vegetation, organic cycles, ecosystem stability, physico-chemical water quality, hydromorphology	swing, direct rating (biology), swing (physical)	yes
Phys	hydraulics, river morphology	10 y	hydromorphology	reversed-swing, direct rating	no

We carried out two series of face-to-face interviews with each of the six experts. Face-to-face interviews are among the most likely to produce high-quality responses [33, 34] compared to remote (online) surveys or group methods. The main aim of the first interview series was to find the minimum set of objectives and attributes to adequately assess the potential ecological improvement after the implementation of a river management action. To facilitate these interviews, we adapted an existing objectives hierarchy, which was constructed previously for the support of river rehabilitation [5, 19, 24]. In the second interview series, we elicited the value functions, the weights of the objectives, the additional scaling factor if experts chose multiplicative (and not additive) aggregation, and the experts’ attitude towards risk. Each interview lasted between two to five hours, was recorded, transcribed, and sent to the experts for feedback.

We followed a rigorous interview protocol including a structured, documented, and systematic protocol for the quantification of expert knowledge (S4 File). It is well known in decision analysis that the elicitation of expert knowledge is prone to subjective biases; with the use of such a protocol, we countered these, including the splitting bias, framing, overconfidence, anchoring, halo effects, dominance, or linguistic uncertainty (e.g. [35, 36–39]).

Compared to unstructured questions, structured elicitation procedures produce more reliable and better-calibrated estimates of facts (e.g. [38]), and thus presumably also of preferences.

### 2.2 Ethics statement

Five of the six experts are active scientists themselves that publish in the field; one expert now works in practice, but has a scientific background (Phys; Table 2). All experts were fully aware of the fact that the information from these interviews would be used to construct an assessment procedure for river rehabilitation, with the aim of a scientific publication. They fully agreed with this, and they received a detailed information package about the content before each interview.

After each interview, the experts received the transcribed interview protocols, which were corrected according to their written feedback, and they agreed in their Email responses that we could use the information to construct an assessment procedure for river rehabilitation. We did not regard it as necessary to pass this study about the quantification of expert opinion (also see e.g. [38] and Bayesian approaches) through an ethics committee beforehand, since we did not ask the experts for any sort of personal, medical, or psychological information. To protect the expert’s anonymity, we do not disclose their names or affiliations (Table 2).

### 2.3 Study site

Assessing the ecological state of a river, including the selection of objectives and attributes as well as their evaluation, strongly depends on the river type. We selected the lower reaches of the Wigger as a study site to test the elicitation simplifications, because it represents a common river type in Switzerland and is well studied (e.g. [40]; see S6 File). The Wigger is a 41 km long, gravel-bed river located in the Swiss Midlands with a catchment area of 368 km^2^. It originates at 1300 m a.s.l., flowing through steep and narrow valleys in its upper reaches. The middle and lower reaches are wider and would naturally be of a braided river type, structured by gravel bars. However, large parts of the lower reaches are channelized, cleared from most of the riparian vegetation, and impacted by surrounding agriculture. The Wigger is a typical trout brook characterized by brown trout (*Salmo trutta*), barbel (*Barbus barbus*), nase (*Chondrostoma nasus*), spirlin (*Albumoides bipunctatus*), and stone loach (*Barbatula barbatula*).

### 2.4 Objectives and attributes

The procedure to identify the objectives and attributes consisted of four steps. In each step, we moved from higher-level to lower-level objectives to avoid the well-known splitting bias (e.g. [18, 41, 42]). We asked the experts i) to come up with necessary objectives and attributes to include, ii) to decide on the final objectives and attributes (now also considering the objectives and attributes elicited in a previous study [24]) and iii) to classify each chosen attribute as “essential”, “very valuable”, or “desirable”. To consolidate the information from all experts, we built the final objectives hierarchy including only those objectives and attributes that were classified as “essential” by at least one expert. All objectives were chosen adhering to the formal requirements of decision theory including comprehensive coverage of the decision problem, being fundamental, complete, concise, and simple [11–13].

The objectives may not necessarily seem non-ambiguous in the sense that they may be difficult to understand for lay persons. However, since the objectives hierarchy in our example was built for expert use, based on expert knowledge in the respective field, this requirement is fulfilled. Hence, we assumed that any biodiversity-expert would understand the goal of having a good fish status and would agree to measure this goal with the suggested attributes. To validate that the proposal of an expert would also match the opinion of another, we confronted each expert with the objectives and attributes identified as essential by one or several of the other experts. Hence, this cross-validation allowed us to be sure that all objectives were well covered by the sub-objectives and attributes and that the attributes were unambiguous and defined in sufficient detail to be easily understood by any other expert in the field. However, in case of strong disagreement, we kept the proposition of each expert, resulting in an individual objectives hierarchy for each expert (S3 File).

Although decision theory also asks for non-redundancy, we allowed for (partly) redundant lower-level objectives and especially attributes. In the context of environmental management this is favourable [6, 21], and probably a rather unique property of environmental decision support compared to other application fields. In any case where an objectives hierarchy is used, an objective is divided into lower-level sub-objectives, whose values are aggregated to the next higher level. If redundant sub-objectives are available, averaging across these data may likely be beneficial: It decreases the uncertainty associated with environmental assessment and the measurement procedure of any environmental indicator and therewith increases statistical significance. Additionally, due to the redundancy of sub-objectives, an assessment can even be performed if not all values are available which is often the case in real-world applications. In this case, simply the weights for the remaining sub-objectives need to be renormalized to prevent biases. To exemplify this: there are several indices available with which we can assess the status of macroinvertebrates in rivers. These redundant indices should give similar assessment results. Averaging these results will provide a value with higher statistical significance than using a single result of only one of the indices. Hence, considering redundant lower-level objectives accounts for the facts that ecosystem valuation is typically based on measurable ecological attributes, that it is uncertain, and also that data are often scarce.

### 2.5 Value functions

In the second interview, we first presented the consolidated objectives hierarchy and adapted objectives and attributes if the expert did not agree with them. Then, we asked each expert for possible measurement methods of the attributes, the measurement units, and the range of each attribute from the worst- to the best-possible state considering a near-natural state of our study river. Based on this information, we elicited the value functions using the bisection method ([11, 12]; Table 1). Each expert elicited the value functions that corresponded with her/his competences (Table 2). Examples of typical questions in an interview to ask for preferences are given in S7 File.

### 2.6 Aggregation methods

We first explained hierarchical aggregation to the experts: objectives at the lowest level of the objectives hierarchy are combined and build the basis for the aggregation at each next-higher level. We then introduced three different methods including additive aggregation, minimum aggregation (also called one-out, all-out; [43]), and multiplicative aggregation (see also S4 File). We chose additive and minimum aggregation as both are widely applied in ecological assessments [44], and multiplicative aggregation which requires weaker preference independence conditions [12, 13, 31] and therefore is a popular alternative to the additive model. We discussed the implications of the three different aggregations for the assessment outcomes [21] and asked the experts to choose a method for each branch of the objectives hierarchy. Depending on the method, we had to elicit different parameters: For additive aggregation [12, 13, 31], we had to identify value functions and weights [that sum up to one), for minimum aggregation value functions, and for the multiplicative aggregation value functions, weights and an additional scaling constant, the synergy factor k (see below).

#### 2.6.1. Additive aggregation

For additive aggregation, the aggregated value is calculated as the sum of the n values, *v*_*i*_, of the sub-objectives each of them multiplied with its weight, *w*_*i*_:

$${\text{f}}_{\text{add}}\left({\text{v}}_{1},\ldots ,{\text{v}}_{\text{n}}\right)\;=\;\sum_{\text{i }=\;1}^{\text{n}}{\text{w}}_{\text{i}}{\text{v}}_{\text{i}}\;={\text{ w}}_{1}{\text{v}}_{1}+{\text{w}}_{2}{\text{v}}_{2}+\ldots +{\text{w}}_{\text{n}}{\text{v}}_{\text{n}}$$ (1)

If the weights are equal for all elements (*w*_*i*_ = 1/n), the result is identical to the (unweighted) arithmetic mean which is often referred to as unweighted averaging. In decision science, the weighted arithmetic mean is called additive aggregation, which is by far the most widely used aggregation function for multi-criteria decision support [12, 13].

#### 2.6.2. Minimum aggregation

For the minimum aggregation the aggregated value, v, is calculated as the minimum of the values, *v*_*i*_, of the sub-objectives:

$$f_{min}(v_{1},\;\ldots ,\;v_{n})\;=\;\text{min}(v_{1},\ldots ,\;v_{n})$$ (2)

The minimum aggregation method indicates a pessimistic attitude that may even hold the potential of a pessimism bias [45], since the aggregated value only reflects the worst sub-objective and does not account for an improvement of a better-performing sub-objective.

#### 2.6.3. Multiplicative aggregation

Despite requiring weaker preference independence, multiplicative aggregation is rarely applied in practice (but see e.g. [46–51]), which could be due to the complexity of the model including the necessity of identifying an additional parameter (the synergy factor k):

$$f_{mult\;}\left(v_{1},\ldots ,v_{n\;}\right)\;=\;\frac{\Pi_{i=1}^{n}\left[k\;w_{i}v_{i}+1\right]-1}{k}$$ (3)

**Fig 1:**
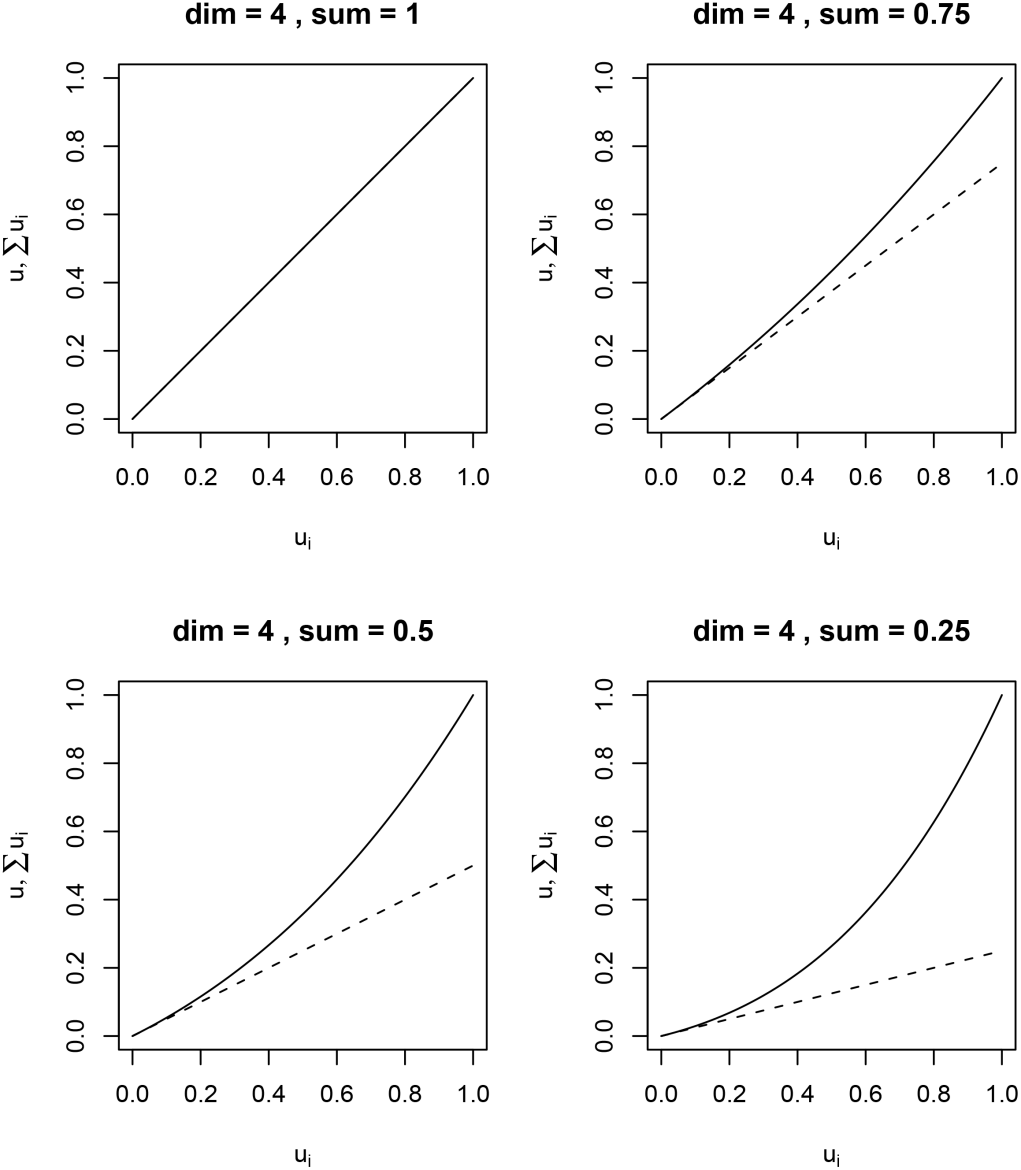
Synergy effect for multiplicative aggregation. Diagram used in the second interviews to discuss implications of multiplicative aggregation using the example of four objectives with equal weights. These objectives can achieve different values between 0 (worst-possible state) and 1 (best-possible state). Solid line: all objectives are increased from a value of 0 to 1 together. Dashed line: value of only one objective at a time is increased from 0 to 1, i.e. this is done for each objective one after the other, and resulting values are summed up. A) No synergy effect (same as additive aggregation), B) small synergy effect of having all objectives on a similarly good level, C) medium synergy effect, and D) large synergy effect. B-D) A better overall value is achieved if all objectives are increased together (solid lines) than if only one individual objective at a time is improved, but not the others (dashed lines).

In our application, this synergy factor could take one of four values including 0.25 (large synergy effect of several attributes having a rather good state), 0.5 (medium synergy), 0.75 (small synergy), and 1 (no synergy effect = additive aggregation), which we assessed directly from the experts [31] with the help of an illustration (Fig 1 and Figures A and B in S4 File). Multiplicative aggregation penalizes options with extremely divergent attribute scores (i.e. some attributes being in a poor state, and some in a high state), while attributes with similar, medium level-outcomes are assessed superiorly (see discussion).

### 2.7 Scaling constants: weights and synergy factor

With each expert, we identified the weights for those lowest-level objectives for which the expert was able to give value functions as well as for all higher-level objectives. To assess the weights, we used the Swing, Reversed-Swing, or the direct rating method (Table 1). Because we were working with scientists who understood the implications, we assessed some of the scaling constants directly although direct rating is not generally recommended (but it is a recommended procedure by [31]). Whenever experts chose multiplicative aggregation, we elicited a synergy factor to define the importance of synergistic effects, i.e. when several objectives are improved simultaneously (Fig 1 and S1 File). For those cases where several experts proposed different weights or different synergy factors for the same objective, we calculated the median over all contributing experts. To exclude unwanted objectives from the assessment, experts were allowed to assign a weight of 0.

### 2.8 Risk attitudes

We only asked about the experts´ risk attitude on the highest-level objective (i.e. "high level of ecological integrity"). Identifying risk attitudes on the lower hierarchical levels requires expertise in the respective branches. However our experts had “only” deep knowledge of a few branches, why we assumed that they would agree with the risk attitude of their fellow expert who does have the required expertise. We elicited the risk attitude with the bisection version of the variable certainty equivalent method (e.g. [12]) from three of the six experts, including the BioA-, BioB-, and BioPhys-expert (Table 2). The risk attitude is needed to translate the values of the state of the river (between 0 = worst state and 1 = best state) to utilities. In a rehabilitation project, one would determine whether the ranking of the possible rehabilitation actions changes when using values or utilities, i.e. without or with risk attitude. If the ranking of the best-performing action(s) does not change, there is no need to go through the extra effort to identify risk attitudes.

To assess the risk attitude, we presented 50–50 lotteries to the experts and asked for the respective certainty equivalent (CE). In our case, the CE is that ecological state of our study-river (defined as a value between 0 and 1) the expert would be willing to accept instead of the presented lottery. First, we asked for the CE x_0.5_. The CE x_0.5_ is the state of the river (given as a value between 0 and 1) that the expert would equally likely accept as a 50–50 lottery (x_min_, 0.5; x_max_, 0.5). Thereby, the 50–50 lottery represents a 50% chance of the river being in its worst state (value = 0) and a 50% chance of being in its best state (value = 1). A risk neutral expert is indifferent between the 50–50 lottery and the river being in a medium state for sure (value = 0.5; corresponding to a linear utility function), and a risk averse expert between the lottery and the river being in a poorer state for sure (i.e. CE < 0.5; for risk prone CE > 0.5). Following the elicitation of the CE x_0.5_ we asked for x_0.75_ and x_0.25_. We carried out consistency checks for some intermediate points (see interview example in S7 File).

## 3. Results

### 3.1 Objectives and attributes

**Fig 2:**
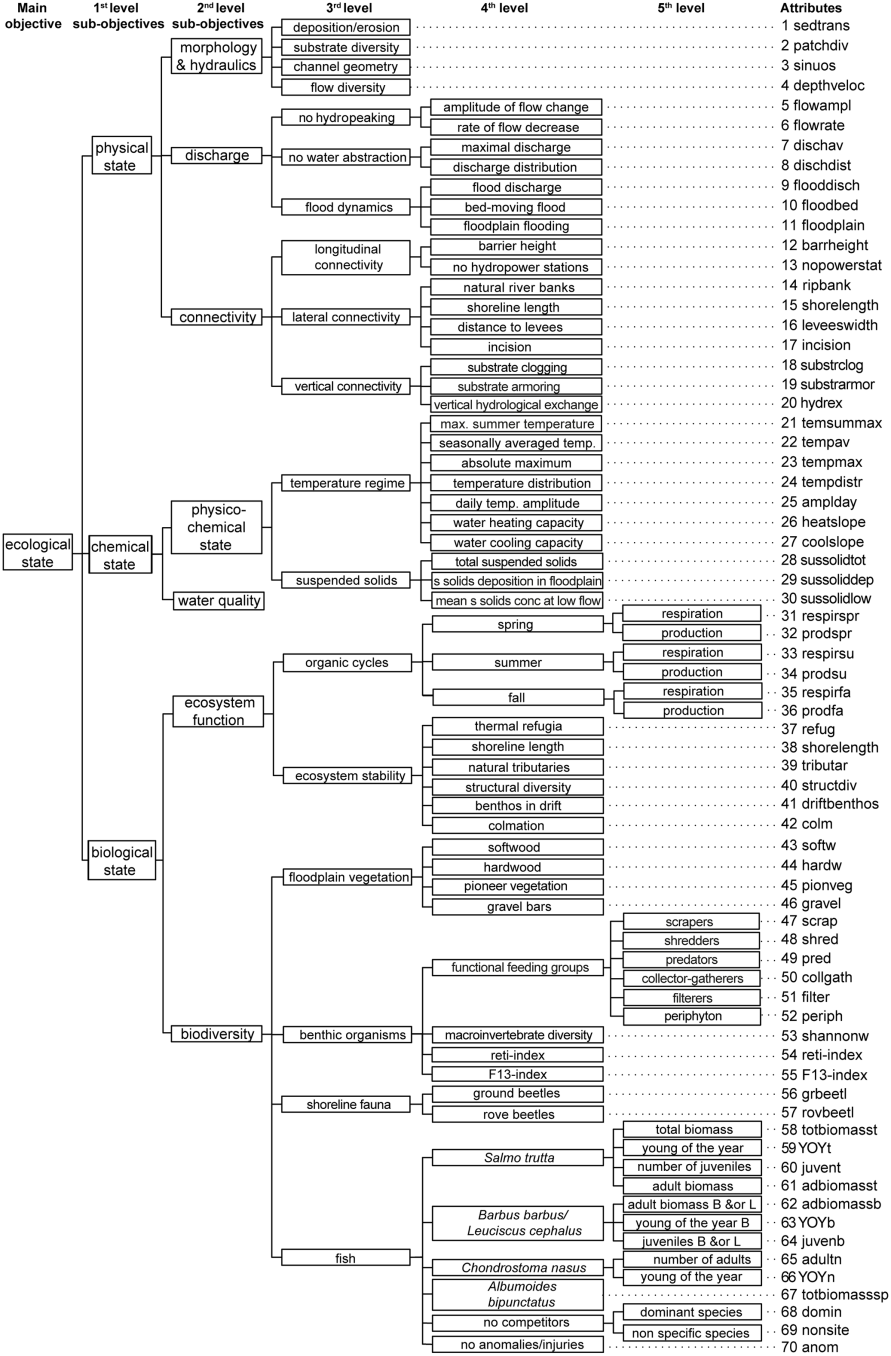
Full objectives hierarchy. This hierarchy shows all the objectives (arranged at levels 1 to 5) and respective measurable system attributes that were considered to be essential by the experts to assess whether a good "ecological state" (main objective) was reached after rehabilitation. Objectives and attributes were identified for the lower, braided reach of the river Wigger in Switzerland, and may therefore change when considering a different river type (abbreviations see Table 3 and S3 Table).

The final objectives hierarchy integrates the objectives and attributes that the experts defined individually as essential to assess a change in the ecological river status after the implementation of a rehabilitation action along the lower reaches of our study stream. Together, the experts identified 102 essential objectives: three on level 1, seven on level 2, 18 on level 3, 51 on level 4, and 23 on level 5 (Fig 2). An individual objectives hierarchy for each expert is given in the S3 File.

Five of the six experts agreed to quantify the main objective on the basis of the three objectives "good physical", "good chemical", and "good biological state" (level 1 of the objectives hierarchy, Fig 2). On the consecutive levels, these experts (Fish, BioB, BioC, BioPhys, and Phys) included 75, 78, 89, 76, and 90 essential objectives, respectively. The BioA-expert included only one objective, namely the "biological state" on the first level, and 53 objectives on the levels 3 to 6.

All experts together identified a total of 70 essential attributes to quantify the lowest-level objectives (level 5, Fig 2 and Table 3). Thereof, the Fish-expert included 50, BioA 37, BioB 51, BioC 60, Phys 61, and the BioPhys-expert 49 attributes in their individual objectives hierarchies (S3 File).

**Table 3:** Attributes. Explanation, units, and ranges (worst possible to best possible level) of the 70 essential attributes included in the objectives hierarchy of Fig 2. Reference river refers to the lower reach of the Wigger. L: attribute and value function from the literature; BA: BioA-expert; BB: BioB-expert; BC: BioC-expert BP; BioPhys-expert; F: fish-expert; P: Phys-expert (see Table 2; more explanations see S3 Table).

No.	Attribute abbr.	Measure	Unit	Range (worst—best)
1	sedtrans	amount of sediment transported downstream per year	m^3^/year	0–3000
2	patchdiv-L	diversity of observed sediment patches	class 1 to 7	7–1
3	sinuos	length of braids per river length	m/m	1–3
4	depthveloc	Shannon Weaver diversity index of Froude numbers	number	0–1
5	flowampl-F	maximal discharge	l/s	28000–4000
5	flowampl-BP	ratio high to low discharge per day	(m^3^/s)/(m^3^/s)	8–0
6	flowrate	rate of decrease of artificial flow	cm/hour	200–10
7	dischav	% deviation of maximal discharge from reference river	%	100–0
8	dischdist	% deviation of 5^th^ percentile of discharge distribution from reference river	%	100–0
9	flooddisch	% deviation of discharge of annual flood from reference river	%	100–0
10	floodbed-BP	relative deviation of frequency of bed-moving floods from reference river	%	100–0
10	floodbed-P	years between riverbed-forming discharges	HQ_xy_	1–3 // 20–5
11	floodplain-BP	relative deviation of frequency of floodplain flooding from reference river	%	100–0
11	floodplain-L	number of floodings per year	n°/year	0–1
12	barrheight	height of artificial barrier	cm	100–0
13	nopowerstat	number of power stations à 1 KW	n°	6–0
14	ripbank	length of natural river banks per total length of both banks	m/m	0–1
15	shorelength	length of the thalweg relative to the total length of both river banks	m/m	2–28
16	leveeswidth	distance between levees compared to total floodplain width	m/m	0.05–1
17	incision	depth of incision	m	5–0
18	substrclog-L	class of substrate clogging	1 to 5	5–1
19	substrarmor	relative values of sigma = sqrt (D16/D84)	number	0–1
20	hydrex	ratio of the observed vertical hydrological exchange between surface and ground water: exchange in a reference river	number	0.0001–1
21	tempsummax	maximum water temperature in summer	°C	24–10
22	tempav	maximum deviation of average water temperature compared to reference river	°C	15–0
23	tempmax	highest water temperature recorded compared to temp. of reference river	°C	15–0
25	amplday	deviation of daily amplitude from reference river	°C	20–0
26	heatslope	difference between heating gradient of assessed and reference river	°C/hour	2–0
27	cooslope	difference between cooling gradient of assessed and reference river	°C/hour	2–0
28	sussolidtot-L	total suspended solids	mg/l	500–0
29	sussolidlow	mean suspended solids concentration at low flow	g/m^3^	No value function
30	sussoliddep	solids´ deposition in floodplain	yes or no	0 or 1
31	respirspr	in-stream respiration in spring	gO_2_/m^2^d	0–7 // 14–7
32	prodspr	in-stream productivity in spring	gO_2_/m^2^d	0–2.5 // 10–2.5
33	respirsu	in-stream respiration in summer	gO_2_/m^2^d	0–5 // 10–5
34	prodsu	in-stream productivity in summer	gO_2_/m^2^d	0–0.5 // 10–0.5
35	respirfa	in-stream respiration in fall	gO_2_/m^2^d	0–10 // 20–10
36	prodfa	in-stream productivity in fall	gO_2_/m^2^d	0–0.5 // 10–0.5
37	refug-BP	area with significant drop in temperature (max. temp.–temp. in certain spot)	m^2^	0–40
37	refug-BB	area with significant drop in temperature (max. temp.–temp. in certain spot)	m^2^	0–50
38	shorelength-BP	shoreline length per channel length	m/m	2–60
38	shorelength-BB	shoreline length per channel length	m/m	2–17
38	shorelength-BC	shoreline length per channel length	m/m	2–4
39	tributar-BP	relative proportion of tributaries in a natural state	%	0–100
39	tributar-BB	relative proportion of tributaries in a natural state	%	0–100
39	tributar-BC	relative proportion of tributaries in a natural state	%	0–100
40	structdiv	rel. proportion of area with deadwood per total river area	%	0–15 // 100–17
41	driftbenthos	rel. proportion of benthos in drift compared to total benthos	%	10–2 // 0–1.5
42	colm	rel. proportion of total interstitial space clogged with fine sediments	%	100–0
43	softw-BP	area of softwood vegetation per wetted channel area per river length	proportion/m	0–5
43	softw-BB	area of softwood vegetation per river length	m^2^/m	0–40
44	hardw	area of hardwood vegetation per wetted channel area per river length	proportion/m	0–6
45	pionveg	area of pioneer vegetation per wetted channel area per river length	proportion/m	0–5
46	gravel-BP	area of gravel bars per wetted channel area per river length	proportion/m	0–2
46	gravel-BB	area of gravel bars per river length	%/m	0–100
47	scrap	rel. proportion of scrapers in the macroinvertebrate community	%	0–30 // 100–30
48	shred	rel. proportion of shredders in the macroinvertebrate community	%	0–20 // 40–20
49	pred	rel. proportion of predators in the macroinvertebrate community	%	0–15
50	collgath	rel. proportion of collector-gatherers in the macroinvertebrate community	%	0–20 // 50–20
51	filter	rel. proportion of filterers in the macroinvertebrate community	%	0–20 // 100–20
52	periph-BA	rel. proportion of periphyton	individuals/m^2^	0–50 // 100–50
52	periph-BB	amount of periphyton biomass per area	g ash free dry mass/m^2^	0–100 // 200–100
53	reti-index	Reti-index for macroinvertebrates: (scrapers + wood-eaters + shredders)/ all feeding types	number	0–50
54	F13-index	F13 Yoshimura-index for macroinvertebrates: (scrapers + filterers)/ (shredders + gatherers-collectors)	number	0.20–1.25
55	shannonw	Shannon Weaver Index	number	0–4
56	grbeetl	mean density of ground beetles	individuals/m^2^	0–50
57	rovbeetl	mean density of rove beetles	individuals/m^2^	0–20
58	totbiomasst	total biomass of trout	kg/ha	20–250
59	YOYt	number of young-of-the-year (age-0 fish) trout	n° of ind.	0–8,000
60	juvent	number of juvenile (age-1 fish to sexual maturity) trout	n° of individuals	0–3,000
61	adbiomasst	total biomass of adult trout	kg/ha	0–150
62	adbiomassb	total biomass of adult barbel and/or chub	kg/ha	0–80
63	YOYb	number of young-of-the-year barbel	n° of ind.	0–3,000
64	juvenb	number of juvenile barbel and/or chub	n° of ind.	0–3,000
65	adultn	number of adult nase	n° of ind.	0–2,000
66	YOYn	number of young-of-the-year nase	yes or no	0–1
67	totbiomasssp	total biomass of spirlin	kg/ha	0–30
68	domin	dominance of any fish species	kg/ha	300–80
69	nonsite	number of non-site-specific species	n° of species	10–0
70	anom	percent fish with anomalies or injuries	%	50–0

### 3.2 Value functions

We established value functions for 69 of the 70 total attributes that were assessed to be essential. The experts provided value functions for 65 of them (S3 File), and we extracted another four from the literature (numbers 2; [52], 11 [53], 18 [26], and 28 [54, 55]; Table 3, S2 File, S2 Table). Neither the experts nor the literature could help us finding a value function for "mean suspended solid concentration at low discharge" (number 30, Fig 2). Since we explicitly excluded the entire branch "good water quality", we did not identify attributes and value functions for this objective.

For each of the eight attributes "flowamp", "floodbed", "refug", "shorelength", "tributar", "softw", "gravel", and "periph", two or three experts gave a value function resulting in a total of 80 value functions (see S1 Table for our suggestion of value functions for a generalized model). The Fish-expert also gave value functions and weights for the mid-reaches and headwaters of the reference river (S2 Table), additionally to the lower reaches presented here. This information is accessible in the Supporting Information, but we do not further discuss it.

**Fig 3:**
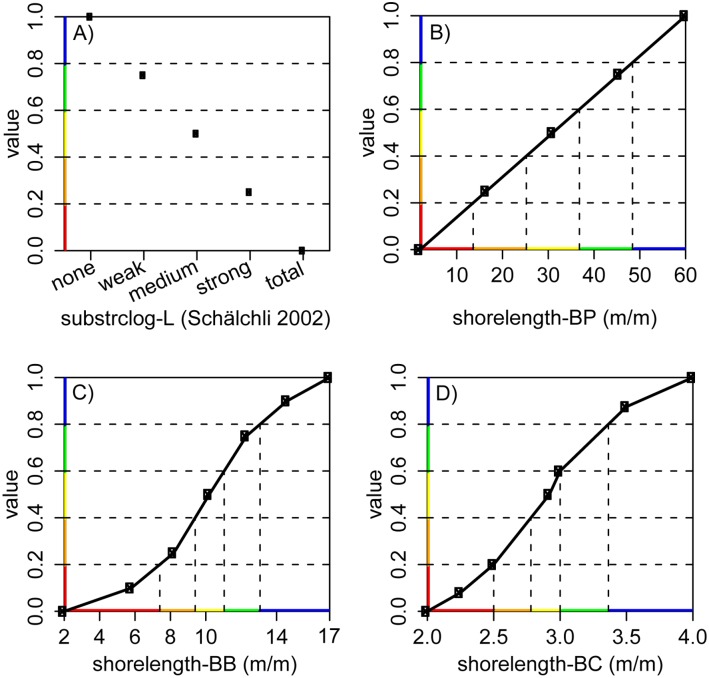
Sample value functions. We show different exemplary value functions for the attributes (A) substrate clogging (n° 18; discrete; Table 3) and (B—D) shoreline length per river length (n° 38; continuous). The BioPhys-expert assessed shoreline length as a linear value function (B), whereas the BioPhys- and the BioC-experts opted for a non-linear one (C and D). x-axis: attribute scale from worst-possible (left) to best-possible state (right). y-axis: value scale, where the attribute level is translated to a neutral value between 0 (worst-possible state) to 1 (best-possible state) with help of the value function. Color-coding according to Swiss Modular Concept of stream assessment in five quality classes: bad, unsatisfactory, moderate, good, and very good (http://www.modul-stufen-konzept.ch/index_EN).

Of the 76 individual value functions from the experts, 74 were continuous (two were discrete) and could therefore be analysed for their shape: 19% were linear and 81% were shaped differently (Fig 3 and S5 File).

### 3.3 Aggregation methods

In 76% of all aggregations (37 cases), the experts preferred multiplicative aggregation. For the remaining twelve cases, they choose additivity (Table 4 and S1 File). The Phys-expert preferred the additive model throughout the entire objectives hierarchy. On the highest-level of the hierarchy, two experts (Fish and Phys) averaged the objectives "physical", "chemical", and "biological state", while two others preferred multiplicative aggregation (BioB and BioPhys; Table 4). BioA did not require aggregation at the highest level, opting to base the entire assessment on the biological branch alone.

**Table 4:** Weights and aggregation. Weights for the objectives on level 1 "physical state", "chemical state", and "biological state", preferred aggregation method (mult = multiplicative, add = additive), and synergy factor elicited from five experts (Fish, BioA, BioB, BioPhys, and Phys). The summary shows the median, minimum, and maximum weight for all of the experts. Symbol Ø: no aggregation required, since the full weight of 1 was given to the "biological state".

Objective	Expert				
	Fish	BioA	BioB	BioPhys	Phys
Physical state	0.32	0	0.31	0.26	0.28
Chemical state	0.23	0	0.31	0.21	0.44
Biological state	0.45	1	0.39	0.53	0.28
Aggregation	add	Ø	mult	mult	add
Synergy effect	1	Ø	0.25	0.5	1

In the biological branch, only one out of 26 aggregations was defined to be additive (mean density of ground and rove beetles; BioA; Table C in S1 File). In the chemical branch, two aggregations were additive ("physico-chemical state" and "water quality"; "total suspended solids", "mean suspended solids concentration at low flow" and "suspended solids deposition in floodplain"; both by the Phys-expert; Table B in S1 File). In the physical branch, seven out of the 14 aggregations were additive, including five assigned by the Phys-expert (aggregation of the three lower-level objectives of "physical state"; the four lower-level objectives of "morphology and hydraulics"; the three lower-level objectives of "discharge"; "bed-moving flood" and "floodplain flooding"; and the three lower-level objectives of "connectivity") and our own assumption for aggregating "barrier height" and "no power stations" (Table A in S1 File).

### 3.4 Scaling constants: weights and synergy factor

We assessed the weights and the synergy factors from at least one, but mostly from more experts with two exceptions: Due to the lack of expert input, we assumed equal weights for the objectives "barrier height" and "no hydropower stations" (Table A in S1 File), and the three objectives "total suspended solids", "suspended solids deposition in floodplain", and "mean suspended solids concentration at low flow" (Table B in S1 File).

We found large variation in weights (min—max ≥ 0.4) elicited for the same objective from different experts in 12 of 85 cases (14%; Table 4 and S1 File). The highest-level weights of the physical, chemical, and biological state covered the whole range from 0 to 1 (Table 4). Four of five experts weighted the biological state (median: 0.45) more than the physical (median: 0.28) and the chemical state (median: 0.23). Contrarily, the Phys-expert weighted the "chemical state" the highest (Table 4). The BioA-expert discarded the chemical and the physical state from the objectives hierarchy, assigning weights of 0 to those objectives.

Weights of the second and lower-level objectives also varied between 0 and 1 with a median weight of 0.31 for the objectives in the physical branch, of 0.20 the chemical branch, and of 0.32 in the biological branch (S1 File).

Also in the lower branches, experts sometimes assigned 0 weights; namely to 34 objectives, therewith excluding them from the assessment: to seven in the physical, three in the chemical, and to 24 in the biological branch (S1 File). For instance in the physical branch, the BioPhys-expert assigned a weight of 0 to the objectives "deposition/erosion", "substrate diversity" and "channel geometry", and "substrate clogging" and "substrate armoring" (Table A in S1 File). Likewise in the chemical branch, "total suspended solids" and "mean suspended solids concentration" (BioPhys and Phys, respectively; Table B in S1 File), and in the biological branch "floodplain vegetation", "benthic organisms", and "shoreline fauna" (Fish; Table C in S1 File) received a weight of 0 from some experts. Hence, weights for these objectives (among some others) differed most (min—max ≥ 0.4). Additionally, there were relatively large weight differences between 0.2 and 0.4 in seven of 29 cases in the physical branch (24%; Table A in S1 File) and in ten of 39 cases (26%) in the biological branch (Table C in S1 File).

There were much larger variations between the experts concerning the additional scaling constant, the synergy factor, which is needed for multiplicative aggregation: In 11 of 27 cases (41%; Table 4 and S1 File), this variation was very large (min—max ≥ 0.4). For aggregating the highest-level objectives (physical, chemical, and biological state), experts chose synergy factors between 0.25 and 1 (median: 0.75; Table 4). For the second and lower-level synergy factors, for the physical branch, experts assigned the largest differences in the synergy effect to the objectives "morphology & hydraulics", "discharge", and "connectivity" at the second level, to the lower-level objectives of "discharge" and "connectivity", and to the lower-level objectives of "flood dynamics". For the biological branch, the largest differences arose from experts preferring small or large synergy effects when aggregating for instance "ecosystem function" and "biodiversity" on the second level, or the different benthic functional feeding groups, and "respiration" and "production" on level five (Table C in S1 File).

### 3.5 Risk attitudes

**Fig 4:**
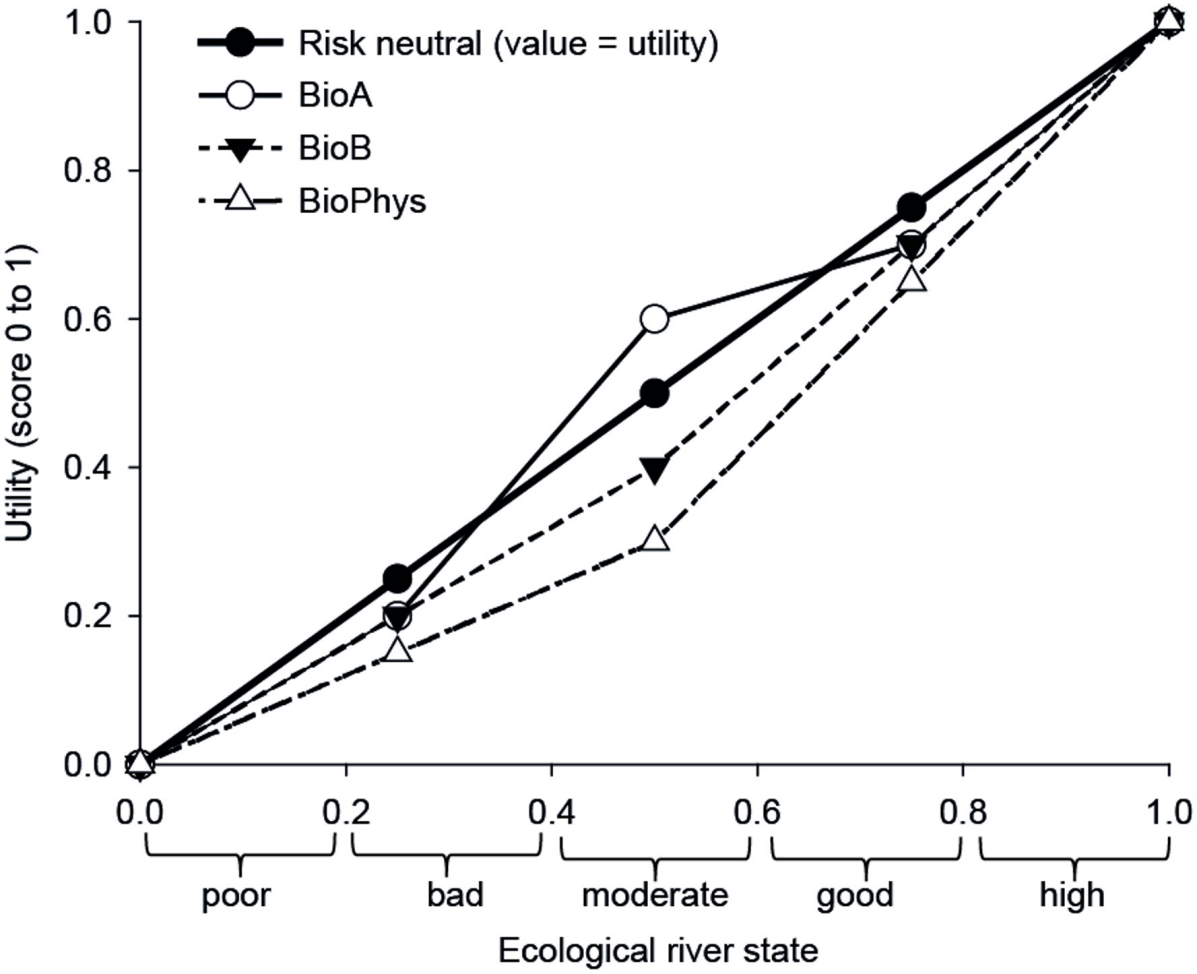
Utility functions elicited from the three experts BioA, BioB, and BioPhys. Utility functions are value functions that include the experts´ risk attitude. A concave utility function implies risk aversion of a decision maker, a convex shape a risk prone attitude. Risk neutrality implies value = utility.

The three experts BioA, BioB, and BioPhys showed a similar trend in their risk attitudes, i.e. slight risk aversion in the case of both a poor (value = 0.25) and a good ecological state of the river (value = 0.75) (Fig 4 and S4 Table). If the river was considered to be in a moderate state (value = 0.5), BioB and BioPhys were slightly risk averse, while BioA was slightly risk prone (utility = 0.6; slightly convex function; S4 Table).

## 4. Discussion

We showed that four commonly applied simplifications in the process of quantifying expert knowledge for MCDA-river rehabilitation did not reflect the opinion of most of our experienced experts, i.e. including only a small number of objectives and attributes and assuming linear value functions, additive aggregation, and risk neutrality. The experts recommended assessing a rehabilitated river with an extensive objectives hierarchy including between 54 and 93 essential objectives, which they decided to aggregate multiplicatively in 37 of the 49 cases (76%). They further proposed between 37 to 61 essential attributes for 81% of which they gave non-linear value functions. We also demonstrated that the experts were either risk averse or risk prone (but never risk neutral) depending on the ecological state of the river (for higher level objectives), and the experts’ personal importance of objectives (at lower levels). Considering these results, we conclude that the sensitivity of these four simplifications on the outcome should either be tested in each case, or that these simplifications should be avoided in preference elicitation if their effect on the assessment outcome is unknown.

### 4.1 Objectives and attributes

Despite the fact that the experts reduced the number of initially selected objectives and attributes (keeping only the essential ones), most of the individual objectives hierarchies remained large (S3 File). The smallest objectives hierarchy was defined by the BioA-expert, who included only biological objectives (Figure B in S3 File). Large objectives hierarchies have two main disadvantages. First, they are time-consuming to develop. However, such an effort may be legitimate if an objectives hierarchy is used repeatedly, for example within a national program. Second, a large hierarchy appears complex and the evaluation in an MCDA may be very time consuming. Therefore, practitioners may be discouraged from applying it. However, as already mentioned in the methods (Section 2.4), when assessing the success of rehabilitations complexity may be advantageous: Redundant attributes increase the statistical significance of assessment outcomes if more than one of them is measured, and they increase flexibility since different users can select their favorite ones to work with [6]. Additionally, complex objectives hierarchies allow identifying the link between an implemented rehabilitation action (i.e. the cause) and its effect(s) on several levels of objectives, therewith enabling learning for future rehabilitations.

The number of objectives does not necessarily need to be large in any MCDA for river rehabilitation. It may vary considerably depending on the complexity of the main objective that has to be quantified: A more complex main objective, such as for example reaching a good ecological river state will always call for more objectives than a simpler main objective such as increasing the rivers’ attractiveness to excursionists. In such cases, multiple interest groups and not only experts might need to be involved. With them, additional socio-economic objectives may need to be discussed (e.g. [5]).

### 4.2 Value functions

Despite linearity being commonly assumed in MCDA-applications [25], 81% of all the value functions that the experts identified were non-linear. Similarly, in a recent study on water infrastructure planning only 23 (14%) of 172 elicited value functions were linear [56]; and in a study on pharmaceuticals in hospital wastewater, 22.5% were linear (18 of 80 value functions elicited from 26 stakeholders; [18, 57]). Hence, non-linearity may be a characteristic of value functions in diverse application types. However, in both of these applications, we found that assuming linearity would not have substantially changed most rank-orders of alternatives.

**Fig 5:**
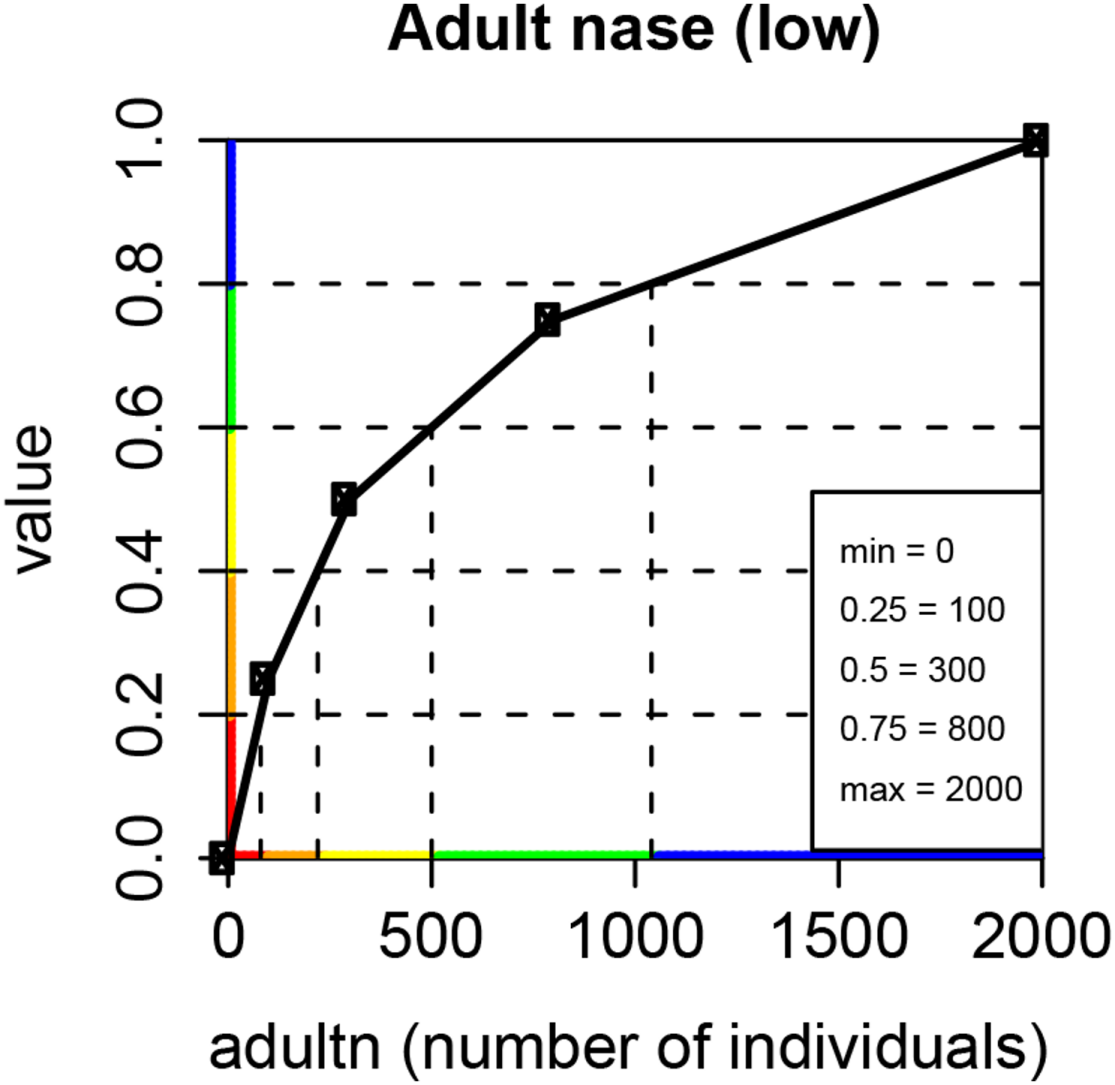
Value function for adult nase. Value function assessed from the fish-expert to describe a natural population of adult nase (*Chondrostoma nasus*). x-axis: attribute scale from left: worst-possible state (0 individuals) to right: best-possible state (2,000 individuals). y-axis: value scale, where the attribute level is translated to a neutral value between 0 and 1, with help of the value function.

In our study, differences in the shape of value functions evolved due to individual preferences among experts. For example, the BioPhys-expert preferred linear relationships arguing that this is the most conservative approach, least prone to errors and biases. One of the stakeholders in the hospital wastewater case study argued similarly [18, 57]. This view contrasted the opinion of the Fish-expert, who argued that it is more important to improve an impacted river than one that is already in quite a good state. Many of the value functions related to fish objectives are therefore asymptotic curves with a steeper value improvement from a bad to a moderate state than from a moderate to a good state. For example, an increase in the population of *Chondrostoma nasus* from 0 to 300 individuals is very steep, because according to our fish expert it is important to have at least some individuals present to sustain a population, while the improvement from 300 to 2,000 individuals (300 = x_0.5_) follows a much flatter curve (Fig 5). Hence, the first few individuals are extremely valuable.

Five of the six experts insisted on directly drawing the value function rather than answering the questions that led to the final curve, arguing that this was easier (exception: BioFish and in some cases BioA and BioB). However, applying a direct rating method is not recommended [12]. To ensure that the shape of the curves represented the preferences of the experts, we posed follow-up questions about the relative preferences using the bisection method ([12]; typical questions see S7 File). In many cases, the curve they had drawn reflected their preferences well. This may be due to the fact that scientists are familiar to the mathematical representation of values. However, since there were exceptions, we strongly recommend following textbook elicitation protocols or if not, carrying out careful consistency checks.

When answering our questions about the shape of value functions, the experts had problems to envisage a corresponding reference condition for some objectives. This is important because the reference defines the upper limit of the attribute (and the worst-possible state the lower limit; [12]). Switzerland is densely populated and a pristine situation (e.g. meandering river) can rarely be achieved with rehabilitation. Therefore, we defined the reference condition to be the best-possible state that can be achieved with an optimal rehabilitation measure, given existing restrictions such as towns, roads, and agriculture [58].

### 4.3 Aggregation methods

Due to the inter-dependencies between objectives, the experts chose multiplicative aggregation in 76% of the cases. On the first level of the objectives hierarchy, two experts (BioB and BioPhys) preferred multiplicative aggregation (Table 4) because the physical, chemical, and biological objectives are strongly connected. Contrarily, two experts (Fish and Phys) argued that these objectives depict largely independent parts of the ecological river system, and therefore favored additive aggregation. As an example, the Fish-expert argued that even in a chemically impaired river, one might not find effects on fish (e.g. changed vitellogenin levels; [59]). This contrasts the possibility of interactions among objectives on the lower levels of the objectives hierarchy, where the Fish-expert opted for multiplicative aggregation.

The Phys-expert preferred additive aggregation throughout all hierarchical levels and branches, even in cases with strong dependencies between the objectives (and preferential dependency). In principle, the additive mathematical model is incorrect to reflect this interdependency and a multiplicative one should be favored. The Phys-expert was aware of the mathematical background (see methods), however argued that for real-world applications a simple model like additive aggregation is preferable: It is more transparent, and thus less viable to manipulation, which is more important than using a model that better reflects interdependencies of objectives.

From a scientific point of view, the Phys-expert considered a mixed minimum-additive model to presumably be most promising [21]. Such an aggregation would account for the undesired properties of the purely additive and purely minimum methods to allow deficits in some objectives being compensated by good scores in others and to only reflect an improvement of the worst attribute in the aggregated value, respectively (see S4 File).

### 4.4 Scaling constants: weights and synergy factor

At the highest level of objectives, four of the five experts weighted the biological state higher than the physical or the chemical state (Table 4). They argued that any physical improvement is irrelevant, if it does not translate into an improvement of the biological condition of the river (see European Commission [44]). Along these lines, BioA suggested that the ecological state of a river could be assessed with biological indicators only (giving zero weight to the chemical and the physical objectives). This view mirrors the assessment scheme adopted by the Water Framework Directive [44], where a rehabilitation action is considered to be unsuccessful if the biological condition is not improved. Contrarily, the Phys-expert considered reaching a good water chemistry to be most important, arguing that a chemically intact river is the basis for the biological elements reaching a good quality.

Such varying views were not limited to the highest-level objectives. The Fish-expert, for example, gave a weight of one to the objective "fish", being the only expert who considered the three other objectives within this same branch to be unnecessary (Figure A in S3 File). He argued that fish are sensitive and can mirror the condition of other species. Similarly in the "morphology & hydraulics"-branch, the BioPhys-expert gave a weight of one to "flow diversity" therewith ignoring the remaining three objectives, while the Phys-expert evaluated all four objectives (i.e. "flow diversity", "deposition/erosion", "substrate diversity", and "channel geometry") almost equally important (Table A in S1 File).

Two camps of opinion are not uncommon in expert elicitation and may arise from ambiguity in framing the problem or because experts have different backgrounds [60, 61]. In our case, it could be attributed to different perspectives: The BioPhys-expert based his opinion on information from the scientific literature, whereas the Phys-expert, who is involved in the implementation of projects on-ground, emphasized the feasibility of the assessment.

Despite these varying views, in only 14% of all aggregations weights differed substantially among the experts (min—max ≥ 0.4), suggesting that the elicited weights are quite robust. However, this did not apply to the synergy factor, which is needed for multiplicative aggregation: In 41%, the synergy factor varied substantially when assessed by different experts. We speculate that this variation resulted from the fact that multiplicative aggregation was new to all experts and therefore they had no pre-established opinions. It may also indicate that we have to improve the protocol for identifying synergy factors from experts to reach better results. Contrary to the assessment of weights, which is routine in practical decision analysis, assessing the additional scaling constant for multiplicative aggregation is very rarely done in MCDA.

### 4.5 Risk attitudes

The three experts from whom we elicited the risk attitude at the highest level of the objectives hierarchy were generally moderately risk averse or risk prone, but never risk neutral (Fig 4). For example, the BioA-expert explained that if the river were in a poor state (value = 0.25) the main objective would be to protect the few existing resources and the functionality of the river, and he would not want to take risks on that (i.e. confirming risk aversion). If the river were in a good state (value = 0.75), he would not want to jeopardize this state (again confirming risk aversion). However, at a medium state of the river (value = 0.5), BioA would be willing to trade a higher risk for a possible (but uncertain) higher ecological state of the river, being slightly risk prone in this case. The other two experts expressed similar opinions.

With the BioPhys-expert, we discussed the assumption that the risk attitude can be different for different objectives [62]. Our expert confirmed this by stating that he would be more conservative for those objectives that are most important to him (i.e. that received higher weights) while for less-important ones he would be more risk prone. He thus confirmed that on the highest level of the objectives hierarchy, he would be rather risk averse, resulting in a concave shape of the utility function for "ecological river integrity" (Fig 4). Therefore, the chosen approach to elicit the risk attitude only at the highest level is justified. It is the most conservative approach, because a change of the risk attitude towards being more risk prone would only occur for some less important lower-level objectives.

Finally, we wish to point out that correctly eliciting preferences is always demanding, but eliciting the utility function with lotteries is an especially difficult task. Below, we recommend several ways to deal with this problem.

### 4.6 The uncertainty in expert knowledge

In our study, value functions, weights, synergy factors, and risk attitudes from different experts (for the same objective) differed from slightly to considerably. This may be due to true differences in the opinion of the experts (see section 4.4), but may also include some systematic bias, such as experts producing systematically optimistic or pessimistic responses (e.g. [36, 37]). Hence, quantifying the uncertainty of expert opinion is an important feature of any elicitation exercise that informs decision makers. It can either be done by quantifying the opinion of multiple experts and combining them mathematically, e.g. by taking a (weighted) group average [63, 64], or by using Bayesian approaches [38, 60, 65, 66]. If only one expert is available, one can ask about the level of precision or certainty about the given weights (best in a face-to-face interview with a feedback cycle; [56, 66]). Thus, we wish to emphasize that large differences in expert judgment, some of which we have observed in our study, are not necessarily bad news. Such differences can even be seen as an advantage of expert elicitation, since they may display different schools of thought in an expert community [60, 61, 67]. For decision making, such varying judgments may best *not* be combined, but rather used to separately analyze how they affect the assessments [68].

### 4.7 Reality check

Our study shows that the four commonly applied MCDA-simplifications do not reflect expert knowledge in the case of river rehabilitation. However, how much expert preferences influence MCDA-outcomes (compared to the simplifications) will be highly case-specific and depend on i) the selection of objectives and attributes, ii) how much the value functions of these objectives deviate from linearity, and iii) the attribute levels measured in the field. Additionally, MCDA-outcomes will be influenced by the choice of the aggregation method, particularly when objectives with significantly different qualities are aggregated [21]. Whether it is worthwhile to carry out an elaborate MCDA to support an application to river rehabilitation, or simply apply the assumptions we have tested, needs to be decided for each case individually. Such a decision may be supported by estimating the relative costs of developing an elaborate MCDA compared to the costs of the rehabilitation implementation. We showcase this idea in the following section for two rehabilitations in Switzerland that differ in river size and complexity regarding the considered objectives, i.e. for the Thur River (large, complex) and the Töss River (mid-size, less complex).

#### 4.7.1 A note on the costs

The objectives hierarchy and the value functions which we have identified in this study build the basis for developing MCDAs for river rehabilitation in other cases. If additional objectives and value functions need to be considered, information may be derived from the literature [6, 20, 69, 70] or from additional expert judgments where needed. We can reasonably assume that the data to predict the outcomes of different rehabilitation actions (i.e. alternatives), e.g. regarding the ecological state or the costs, are available. However, these data might not be in such a form suitable for MCDA and might thus need additional data collection, for example as expert interviews. Based on these assumptions, we estimate that a consultant with some MCDA-expertise can develop an MCDA for the Töss River within four months, which would cost approximately 101’623 CHF including 8% VAT (salary = 170 CHF/hr; 150 hrs/month; based on official Swiss government recommendations; [71]). The rehabilitation project along the Töss River, which included widening 200 m of the river course and revegetating the river banks, cost 0.5 Mio CHF [70]. Thus, the costs for developing the MCDA are 20% of the 0.5 Mio investment costs, which is considerable. However for the more complex case, i.e. the Thur River, the costs for developing an MCDA would only be approximately 3–6% of the total costs of the rehabilitation project of 6 Mio CHF (widening 1500 m of the river course plus rehabilitating the river banks), which probably makes the MCDA worthwhile. Hereby, we assumed that a skilled senior consultant (197 CHF/hour incl. 8% VAT; 150 hrs/month; [71]) works on the case full time for six months (176’707 CHF) to one year (353’415).

Costs of river rehabilitation vary strongly depending on the number of objectives that should be improved (ecological state, flood protection, recreational and aesthetic value) and the magnitude of improvement targeted (e.g. small or large improvement of ecological state), the length and the location of the stretch to be restored (e.g. rehabilitation within cities are more expensive than outside of populated areas due to higher land prices), the chosen measure (e.g. one-sided or two-sided bank rehabilitation etc.; see [69] for costs of different measures), the stakeholders involved and the magnitude of their (dis-)agreement, the size of the involved river [70], and the country of the implementation. Also the costs for developing an MCDA vary among countries due to different salary levels: It is therefore impossible to give general cost estimates. However, relative costs of developing an MCDA compared to the implementation costs are most likely in the same order of magnitude. As stated above, whether it is worthwhile to carry out an elaborate MCDA from scratch depends very much on the case itself. Hence we fully agree with [72]: “What exactly is done at each step of an SDM (“Structured Decision Making”) process, to what level of rigor and complexity, will depend on the nature of the decision, the stakes and the resources, and timeline available.” Finally, costs for developing MCDAs in future river rehabilitation projects will decrease considerably, since they will benefit from information gathered in previous applications.

#### 4.7.2 Minimizing costs

Despite the above remarks, we are aware that eliciting individual preferences from experts, instead of adopting simplifications, may considerably increase the efforts and costs of MCDA-applications. Hence to minimize these costs, we recommend the following:

1. Decide whether it is justified to develop a large, comprehensive objectives hierarchy, e.g. if it is repeatedly or extensively used. In other cases, rely on existing objectives hierarchies (e.g. this one for similar rivers as the Wigger) and adapt to the specific case. Ask experts to rank their objectives according to different categories (e.g. essential, very valuable, or desirable) and only keep essential ones to reduce the number of objectives.
2. Reduce the effort for determining the entire shape of the value function by asking for only the mid-value point x_0.5_ [18, 57]. The mid-value point will indicate whether the value function is concave, convex or linear. Use sensitivity analyses to determine whether more-detailed follow-up questions are required. Letting experts/scientists draw the value functions instead of answering questions to identify their preferences will also reduce the elicitation effort. If done so, ensure the accuracy of their shape with consistency checks.
3. To ease the difficult elicitation of utilities with lotteries, (a) elicit value functions and transform them to utilities at the highest level of the objectives hierarchy, (b) assume risk aversion as a conservative approach [62], (c) elicit only value functions but carry out extensive sensitivity analysis to determine whether the risk attitude affects the ranking of alternatives [56], (d) only roughly determine the risk attitude of the expert which should suffice to allow ordering the alternatives [56], or (e) elicit risk attitudes for different parts of the highest-level value function and for objectives at different hierarchical levels from only a few experts.

## 5. Conclusions

Optimizing river rehabilitation through the prioritization of rehabilitation measures has gained momentum and will become a common tool in river rehabilitation planning [73]. The here presented MCDA-approach is based on a solid, conceptual foundation, which is a crucial aspect of a decision support methodology for environmental management [6]. Practical implementation is another key element, but transferring insights from theory to practice is by no means trivial. In this paper, we document an extensive expert elicitation exercise, and provide ample material for discussion and use in applied river rehabilitation projects (documented in the Supporting Information). Eliciting preferences from experts instead of applying simplified assumptions will improve the representation of ecological expert knowledge and will contribute towards an informed optimization of holistic on-the-ground river rehabilitation. The optimal level of model complexity, however, will always be highly case-study specific depending on data and resource availability, the context, and the complexity of the decision problem.

## Supporting Information

S1 File Weights and aggregation. Information identified from the experts for the A) physical, B) chemical, and C) biological objectives.
(PDF)

S2 File Value functions translated from the literature. Information from the literature for the attributes A) “diversity of sediment patches”, B) “frequency of floodplain flooding”, C) “substrate clogging”, and D) for “total suspended solids”, translated into value functions (E).
(PDF)

S3 File Objectives hierarchies for the individual experts. Objectives hierarchies identified by the A) Fish-, B) BioA-, C) BioB-, D) BioC-, E) BioPhys-, and the F) Phys-expert.
(PDF)

S4 File Illustrations used to support the interviews. A) Aggregation methods for value functions, and B) synergy effect for multiplicative aggregation.
(PDF)

S5 File Value functions for the different objectives. (PDF)

S6 File Description of the reference river, the Wigger (Switzerland). (PDF)

S7 File Example of an interview to elicit preferences. (PDF)

S1 Table Selection of expert value functions for the generalized model. (PDF)

S2 Table Value functions elicited from six river experts. Identified attribute levels for the worst-possible state (value = 0), for the values 0.1, 0.25, 0.5, 0.75, and 0.9, and the best-possible state (value = 1) are shown.
(PDF)

S3 Table Comments value functions. Attribute name, abbreviation, and details concerning the attribute or shape of the value function.
(PDF)

S4 Table Risk attitudes. Certainty Equivalents (CE) elicited from the BioA-, BioB-, and BioPhys-expert.
(PDF)
