# Supplementary material for: Four Common Simplifications of Multi-Criteria Decision Analysis do not hold for River Rehabilitation
Source: PLoS One. 2016 Mar 8;11(3):e0150695. doi: 10.1371/journal.pone.0150695 (PMC4783037; doi:10.1371/journal.pone.0150695)
Supplement: S1 File — Information identified from the experts for the A) physical, B) chemical, and C) biological objectives. (PDF) [file pone.0150695.s001.pdf]

Selected aggregations and scaling factors (weights and synergy effect)

Good physical state

Of the three main physical sub-objectives, natural river morphology and hydraulics is more important, according to the Phys-expert, than the natural discharge regime and good connectivity. This is also how the Swiss Federal Office for the Environment approaches river rehabilitation projects: the morphology and hydraulics can be influenced relatively easily, while the two other objectives are more difficult to change. In contrast, the BioPhys-expert assigned nearly half of the total weight to good connectivity.

Natural river morphology and hydraulics

According to the Phys-expert, natural deposition/ erosion is most important, followed by natural channel geometry. However, Phys had some difficulty to assign the scaling constants, because the attributes are not fully independent. For instance, if the deposition/ erosion is in a really bad state, the other attributes will be in a bad state as well. Despite this, he preferred additive aggregation, because it is the simplest model and well understandable to practitioners. He rated this as more important than using the multiplication aggregation model, which might better reflect interdependencies of certain indicators (see Discussion, main text).

According to the BioPhys-expert, only one attribute is required to describe river morphology and hydraulics. He suggested using the Shannon Weaver diversity index of Froude numbers (see S2 Table).

Natural discharge regime

Both experts judged “no water abstraction” as least important. The Phys-expert had a clear preference for “no hydropeaking” being the most important attribute (0.44), while BioPhys rated this (0.39) as about equally important as “natural flood dynamics” (0.38).

The two attributes of “no hydropeaking” affect two different ecosystems, according to the Phys-expert. Therefore, he regarded the weight distribution as a personal preference. BioPhys argued that if a river is affected by hydropeaking, “no hydropeaking” and “natural flood dynamics” are most important. If it is not affected by hydropeaking, then the “frequency of floodplain flooding” is most important. This attribute was rated as most important descriptor of “natural flood dynamics” by BioPhys, and it automatically becomes more important, if “no hydropeaking” can be ignored.

For “natural flood dynamics”, the “frequency of bed-moving floods” affects the ecosystem river itself, which is why it received the highest weight from the Phys-expert. The “frequency of floodplain flooding” does not affect the ecosystem river itself, but rather the adjacent floodplains. In contrast, “frequency of floodplain flooding” was rated highest by BioPhys. This difference between the two experts was explained by the Phys-expert as follows: following the environmental protection agency, he took the stance of the river itself, rather than that of the adjacent areas, which is why attributes directly related to the river received higher weights. However, this can change according to ones’ ecosystem understanding.

Good connectivity

The Phys-expert judged vertical connectivity most important (0.40), because this defines whether there are any habitats at all. If there is very strong colmation (no vertical connectivity) – if everything sticks together – there will not be any habitats. In contrast, BioPhys rated the vertical connectivity as least important (0.11). The Phys-expert regarded the other two attributes as a bit less important, because they define the size of the habitats. With respect to fish, the longitudinal connectivity was

judged a bit more important than the vertical connectivity, but this might be different for other species. The reasoning of BioPhys was that the geomorphology, reflected in lateral and longitudinal connectivity is most important.

We discussed with BioPhys, whether "high longitudinal connectivity" should be dropped altogether. His reasoning was that it should and would be included in the network assessment, but is of less importance for a river stretch. However, because there can still be artificial drops etc. in a shorter river stretch, we agreed that this attribute can be important. Since BioPhys did not regard himself as competent to answer more-detailed questions concerning this attribute, we elicited the value function for the "barrier height" (height of artificial barriers) from the Fish-expert, together with "no hydropower stations". The latter attribute was included, because power stations can easily cause fish kills.

Table A. Weights and aggregation for the sub-objective "physical state". The value of the synergy effect needed for multiplicative aggregation (0.25 = large, 0.5 = medium, 0.75 = small) was elicited from the BioPhys- and the Phys-experts. A synergy effect of 1 implies additive aggregation. In two cases, we had to make assumptions (Assumpt), because we did not receive information from the experts. Hereby, we assumed equal weights for the respective sub-objectives and additive aggregation. The summary shows the median, and the difference between maximum (Max) and minimum (Min) weights/ synergy effect. (a): difference is between 0.2 and 0.4; (b) difference >0.4. Symbol Ø: no synergy effect possible, since the full weight of 1 was given to only one of the respective objectives; or not relevant in case of only 1 expert.

Level	Sub-objective	BioPhys	Phys	Assumpt	Median	Max–Min	
2	Morphology & hydraulics	0.27	0.46		0.37	0.19	
2	discharge	0.24	0.27		0.26	0.03	
2	connectivity	0.49	0.27		0.38	0.22	(a)
Synergy effect		0.25	1		0.63	0.75	(b)
3	deposition/erosion	0	0.33		0.17	0.33	(a)
3	substrate diversity	0	0.20		0.10	0.20	(a)
3	channel geometry	0	0.27		0.14	0.27	(a)
3	flow diversity	1	0.20		0.60	0.80	(b)
Synergy effect		Ø	1		1	Ø	
3	no hydropeaking	0.39	0.44		0.42	0.05	
3	no water abstraction	0.23	0.22		0.23	0.01	
3	flood dynamics	0.38	0.35		0.37	0.03	
Synergy effect		0.50	1		0.75	0.50	(b)
4	amplitude of flow change	0.43			0.43	Ø	
4	rate of flow decrease	0.57			0.57	Ø	
Synergy effect		0.50			0.50	Ø	
4	maximal discharge	0.50			0.50	Ø	
4	discharge distribution	0.50			0.50	Ø	
Synergy effect		0.50			0.50	Ø	
4	flood discharge	0.29	0		0.15	0.29	(a)
	bed-moving flood	0.29	0.54		0.42	0.25	(a)
4	floodplain flooding	0.41	0.46		0.44	0.05	
Synergy effect		0.50	1		0.75	0.50	(b)
3	longitudinal connectivity	0.44	0.32		0.38	0.12	
3	lateral connectivity	0.45	0.28		0.37	0.17	
3	vertical connectivity	0.11	0.40		0.26	0.29	(a)
Synergy effect		0.25	1		0.63	0.75	(b)

Level	Sub-objective	BioPhys	Phys	Assumpt	Median	Max–Min	
4	barrier height			0.50	0.50	Ø	
4	no hydropower stations			0.50	0.50	Ø	
Synergy effect				1	1	Ø	
4	natural river banks	0.25			0.25	Ø	
4	shoreline length	0.25			0.25	Ø	
4	distance to levees	0.25			0.25	Ø	
4	incision	0.25			0.25	Ø	
Synergy effect		0.50			0.50	Ø	
Level	Sub-objective	BioPhys	Phys	Assumpt	Median	Max–Min	
4	substrate clogging	0	1[1]		0.5	1	(b)
4	substrate armoring	0	1[1]		0.5	1	(b)
4	vertical hydrological exchange	1	0		0.5	1	(b)
Synergy effect		Ø		1	1	Ø	

[1] The Phys-expert suggested using either "substrate clogging" or "substrate armoring". However, he could not give a value function, and did not know which attribute to choose, but stated that one of the two would be sufficient – i.e. either "substrate clogging" or "substrate armoring" would receive a weight of 1, respectively.

Good chemical state

Table B. Weights and aggregation for the sub-objective "chemical state". The value of the synergy effect needed for multiplicative aggregation (0.25 = large, 0.5 = medium, 0.75 = small) was elicited from the BioPhys- and the Phys-experts. A synergy effect of 1 implies additive aggregation. The summary shows the median, and the difference between maximum (Max) and minimum (Min) weights/ synergy effect. (a): difference is between 0.2 and 0.4; (b) difference >0.4. Symbol Ø: no synergy effect possible, since the full weight of 1 was given to only one of the respective objectives; or not relevant in case of only 1 expert.

Level	Sub-objective	BioPhys	Phys	Median	Max–Min	
2	physico-chemical state	0.54		0.54	Ø	
2	water quality	0.46		0.46	Ø	
Synergy effect		0.50	1	0.75	0.5	(b)
3	temperature regime	0.50		0.50	Ø	
3	suspended solids	0.50		0.50	Ø	
Synergy effect		0.25		0.25	Ø	
4	maximal summer temperature	0		0	Ø	
4	seasonally averaged temperature	0.12		0.12	Ø	
4	absolute maximal temperature	0.12		0.12	Ø	
4	temperature distribution	0.21		0.21	Ø	
4	daily temperature amplitude	0.24		0.24	Ø	
4	water heating capacity	0.19		0.19	Ø	
4	water cooling capacity	0.12		0.12	Ø	
Synergy effect		0.50		0.50	Ø	
4	total suspended solids		0[1]	0	Ø	
4	mean suspended solids concentration		0[1]	0	Ø	
4	solids deposition in floodplain		1[1]	1	Ø	
Synergy effect			1	1	Ø	

[1] The BioPhys-expert required two attributes ("total suspended solids" and "mean suspended solids concentration at low discharge"), but could not give weights and a value function. We derived a value function for "total suspended solids" from the literature (but not for "mean suspended solids concentration at low discharge"). The Phys-expert

required one attribute and suggested "solids deposition in floodplain" as an occurrence/no occurrence (yes/no) attribute.

Good biological state

All four experts gave equal weights to "ecosystem function" and "biodiversity", but the synergy effect for aggregation ranged from 0.25 to 0.75.

Ecosystem function

According to the BioA-expert, the sub-objectives "organic cycles" of "ecosystem function" have some redundancies to "benthic organisms" (see below). For this reason, BioA would give them lower weights (0.33) compared to "ecosystem stability (0.67). He argued that there is some synergy between these two sub-objectives, but that they do depict something different. BioPhys had the same weights as BioA, while the ranking was the same for BioB, but the difference between weights was a bit less extreme.

Ecosystem function: organic cycles

Hierarchical aggregation was proposed by the BioPhys-expert, i.e. to first aggregate mean respiration and production for each season, followed by an aggregation of the values obtained for the three seasons spring, summer, and fall. This proposition was double-checked with BioB, who agreed that there can be different respiration rates and primary production in the different seasons. However, while BioPhys would give equal weights to all three seasons (0.33), BioB proposed to double the weights for spring and summer (0.4 each), compared to fall (0.2). Her rationale was that spring and summer are the more active seasons, which thus have more influence on processes and organisms.

Ecosystem function: ecosystem stability

According to the BioC-expert, the sub-objectives of ecosystem stability should receive equal weights, because he did not have a plausible explanation for different weightings. This contrasts the view of the other two experts, who did not distribute the weights equally. However, all experts recommended a relatively small synergistic effect to aggregate the sub-objectives of ecosystem stability, ranging from 0.5 to 0.75.

Biodiversity

All three experts, except the Fish-expert (who only required fish diversity) would aggregate fish diversity, shoreline fauna, benthic organisms, and floodplain vegetation with a multiplicative model, albeit with relatively low (0.60) over medium (0.50) to large (0.25) synergy effects. According to the BioA-expert, all four sub-objectives are necessary to describe different information concerning the functionality of the ecosystem; they thus received equal weights from him. However, statistically the four sub-objectives would be very strongly correlated, which is the reason that he suggested a medium synergy effect. The BioA-expert argued that it would be possible to select from these sub-objectives depending on the goal of the rehabilitation project, or possibly also to reduce the assessment of biodiversity to only one of the four sub-objectives, given sufficient experience. This supports the choice of only fish diversity by the Fish-expert. A similar line of reasoning was followed by BioB, who excluded the shoreline fauna from the assessment, since this would already be reflected by the floodplain vegetation. For instance, if gravel bars are in a good state, there is a high chance of also finding beetles.

Biodiversity: floodplain vegetation

The BioB-expert pointed out that the floodplain vegetation might not consist of much vegetation at all, depending on the floodplain (e.g. gravel dominated floodplains). BioB proposed to exclude pioneer vegetation, because they are reflected in the attribute concerning gravel bars, on which the pioneer vegetation grows. If there are no gravel bars, there will not be any pioneer vegetation. If there are gravel bars and there is a natural flood regime, there will also be pioneer vegetation. Both remaining attributes, softwood vegetation and gravel bars, were rated as equally important by the BioB-expert.

Biodiversity: benthic organisms

The biologist BioA gave detailed explanations concerning the weighting of benthic organisms. Using the Swing method, he first set the "relative proportion of scrapers" from the worst to the best-possible state. The reason was that once the condition of a benthic feeding group is improved, it is natural for the others to follow. Scrapers are at the beginning of this chain, and if scrapers are in a good state, it is likely that also the others will be in a good state. Scrapers are indicators of sufficient, but not too much light. Second, the BioA-expert set shredders to their best state (and scrapers to worst again), because they also stand at the beginning of the food chain. The other feeding groups are dependent on a certain amount of biomass input. Scrapers and shredders are the most integrative feeding groups. Third, predators were put to their best state (and shredders to worst again). They are indicators of the history of the river, i.e. that no massive disturbance or impairment occurred during the last two to three years. If these three indicators (feeding groups) are in a very good state, the others would also be in a good state. So they should receive at least half or more of the overall weight given to benthos (0.87).

A problem with the weighting of the different feeding groups is that they are dependent of each other. If there is a very good assessment for scrapers, it is probable that also shredders receive a good assessment. Or the other way around: if one feeding group is in a very good state, it is rather unlikely that the others would be in a very bad state. The BioA-expert suggested using multiplicative aggregation with a small correction for this synergy where the weighted sum of all benthic feeding groups amounts to 0.75.

The BioB-expert considered all benthic organisms as equally important, and proposed a large synergy effect of 0.25 for multiplicative aggregation. According to the river-continuum-concept, the constitution of the respective feeding groups depends on the river stretch. At higher altitudes, i.e. in forest zones, more filterers and periphyton are to be expected. Since our reference river, the Wigger is not fringed by riparian forest, all benthic feeding groups would be required.

As alternative to the benthic feeding groups, it is possible to use any suitable index, that reflects natural diversity of macroinvertebrates. This option was proposed by the BioB- and BioPhys-expert. This index does not have an optimum; the more diversity, the better (see S5 File). We did not include this additional diversity index in the objectives hierarchy.

Biodiversity: shoreline fauna

The BioA-expert found these attributes important because they depict something different to benthic organisms (see above). Beetles react very quickly to river restoration. Initially, we suggested including spiders as indicator organisms, but BioA would only require the mean density of rove beetles and ground beetles for this assessment. There is a relatively large redundancy between ground beetles and spiders; both are present on dry gravel banks, while rove beetles are more likely to be found on damper habitats. According to BioA, more information can be derived from ground beetles

than from spiders. Because ground beetles are more typical for our reference river (Wigger) with many gravel banks, they received somewhat more weight for this application (0.57).

Biodiversity: fish

The scaling constants for "biodiversity" were elicited very carefully from the Fish-expert. Separate interviews were carried out to elicit the fish diversity in the lower reaches of the Wigger, our reference river, but also the mid reaches and head waters (values presented in S2 Table; important insights are discussed below). We also used these consecutive interviews as consistency checks, since the elicited weights would sometimes differ. Some were revised, while for others, the Fish-expert would justify using different weights.

Fish biodiversity: importance (weights) of sub-objectives along the river gradient

If more and more species are present from the headwaters to the lower reaches, the weights of some attributes should change. Because there is less biomass upstream than downstream, weights attributed to total biomass of trout (*Salmo trutta*) were lower (only 0.3) in the head waters, but higher (0.4) in the mid and lower reaches. The same pattern applied to barbel (*Barbus barbus*), where "total adult biomass" was 0.33 in the mid reaches, but 0.60 in the lower reaches (barbel does not occur in head waters).

On the other hand, "young of the year" (YOY) are most important in headwaters, where they received the highest weights. Hence, "young of the year trout" received a weight of 0.3 in headwaters, but only 0.25 and 0.2 in mid and lower reaches, respectively. Similarly, the "young of the year barbel" received a weight of 0.33 in the mid reaches, but only 0.20 in the lower reaches.

According to the Fish-expert, a natural nase population (*Chondrostoma nasus*) in the lower reaches of the Wigger is something special.

The objective "no competitors" should receive a higher weight in mid reaches (0.20) than in lower reaches (0.13), according to the Fish-expert. The same applies to one of its attributes, the "dominant species (i.e. the dominance of any fish species). The weight of this attribute must be lower in the lower reaches (0.50) than in the mid-reaches (0.60). The reason is that one can expect other than the indicator fish species to enter the lower reaches of a river, and in some cases, they can even become the dominant species.

In mid-reaches, stone loach (*Albumoides bipunctatus*) should be used as an indicator, even if it received only a low weight (0.05). However, after re-consideration by the Fish-expert, this attribute was deleted from the assessment of the lower reaches, because it becomes practically irrelevant due to the low weight (0.01).

Fish biodiversity: aggregation of sub-objectives

To aggregate the lowest-level fish nodes to the next higher level, the Fish-expert usually preferred multiplicative aggregation with a large synergy effect (0.25). There were some discussions with the Fish-expert to determine, whether minimum aggregation would be appropriate for the objective "no anomalies or injuries". This would mean that any percentage of fish with anomalies below a certain threshold would result in an equally bad valuation, i.e. it would always receive the worst value of 0, regardless of the state of the other attributes. However, the Fish-expert then decided that multiplicative aggregation with a large synergy effect would be more appropriate. His rationale was that a bad state of anomalies or injuries in fish can always be compensated to a certain degree if the other attributes are in a good state. However, the Fish-expert also strongly rejected a fully compensatory model as reflected by additive aggregation. This topic was explored with trade-off

questions. For instance, a good state of the nase population in the lower reaches of the Wigger could never fully compensate for a large number of fish (e.g. 50%) with anomalies or injuries. Hence, the multiplicative model with a strong synergy effect was usually found appropriate by the Fish-expert.

Table C. Weights and aggregation for the sub-objective "biological state". The value of the synergy effect needed for multiplicative aggregation (0.25 = large, 0.5 = medium, 0.75 = small) was elicited from the Fish-, BioA-, BioB-, BioC-, and BioPhys-experts. A synergy effect of 1 implies additive aggregation. The summary shows the median, and the difference between maximum (Max) and minimum (Min) weights/ synergy effect. (a): difference is between 0.2 and 0.4; (b) difference >0.4. Symbol Ø: no synergy effect possible, since the full weight of 1 was given to only one of the respective objectives; or not relevant in case of only 1 expert.

Level	Sub-objective	Fish	BioA	BioB	BioC	BioPhys	Median	Max–Min	
2	Ecosystem function	0.50	0.50	0.50		0.50	0.50	0	
2	Biodiversity	0.50	0.50	0.50		0.50	0.50	0	
Synergy effect		0.25	0.75	0.25		0.60	0.43	0.5	(b)
3	Organic cycles		0.33	0.44		0.33	0.33	0.11	
3	Ecosystem stability		0.67	0.56		0.67	0.67	0.11	
Synergy effect			0.50	0.50		0.60	0.50	0.10	
4	Organic cycles spring			0.40		0.33	0.37	0.07	
4	Organic cycles summer			0.40		0.33	0.37	0.07	
4	Organic cycles fall			0.20		0.33	0.27	0.13	
Synergy effect				0.50		0.50	0.50	0	
5	Respiration, spring			0.50		0.57	0.54	0.07	
5	Production, spring			0.50		0.43	0.47	0.07	
Synergy effect				0.25		0.70	0.48	0.45	(b)
5	Respiration, summer			0.50		0.57	0.54	0.07	
5	Production, summer			0.50		0.43	0.47	0.07	
Synergy effect				0.25		0.70	0.48	0.45	(b)

Level	Sub-objective	Fish	BioA	BioB	BioC	BioPhys	Median	Max–Min	
5	Respiration, fall			0.50		0.57	0.54	0.07	
5	Production, fall			0.50		0.43	0.47	0.07	
Synergy effect				0.25		0.70	0.48	0.45	(b)
4	Thermal refugia			0.27	0	0.43	0.27	0.43	(b)
4	Shoreline length			0.18	0.25	0.43	0.25	0.25	(a)
4	Natural tributaries			0.24	0.25	0.14	0.24	0.11	
4	Structural diversity			0.30	0	0	0	0.30	(a)
4	Benthos in drift			0	0.25	0	0	0.25	(a)
4	Colmation			0	0.25	0	0	0.25	(a)
Synergy effect				0.50	0.75	0.70	0.70	0.25	(a)
3	Floodplain vegetation	0	0.25	0.26		0.33	0.26	0.33	(a)
3	Benthic organisms[1]	0	0.25	0.37		0.33	0.29	0.37	(a)
3	Shoreline fauna	0	0.25	0		0	0	0.25	(a)
3	Fish	1	0.25	0.37		0.33	0.35	0.75	(b)
Synergy effect		Ø	0.50	0.25		0.60	0.50	0.35	(a)
4	Softwood			0.5		0.17	0.34	0.33	(a)
4	Hardwood			0		0.33	0.17	0.33	(a)
4	Pioneer vegetation			0		0.17	0.09	0.17	
4	Gravel bars			0.50		0.33	0.42	0.17	
Synergy effect				0.25		0.60	0.43	0.35	(a)
4	Functional Feeding groups[1]		1	1		0	1	1	(b)
4	Macroinvertebrate diversity (Shannon Weaver index)[1]		0	1		1	1	1	(b)
4	Reti-index[1]		0	0		0	0	0	
4	F-13-Yoshimura-index[1]		0	0		0	0	0	
Synergy effect[2]			Ø	Ø		Ø	Ø		
5	Scrapers		0.43	0.17			0.30	0.26	(a)
5	Shredders		0.33	0.17			0.25	0.16	
5	Predators		0.11	0.17			0.14	0.06	
5	Collector-gatherers		0.04	0.17			0.11	0.13	
5	Filterers		0.04	0.17			0.11	0.13	
5	Periphyton		0.04	0.17			0.11	0.13	
Synergy effect			0.75	0.25			0.50	0.50	(b)
4	Ground beetles		0.57	0			0.29	0.57	(b)
4	Rove beetles		0.43	0			0.22	0.43	(b)
Synergy effect			1	Ø		Ø	1	0	

[1] To evaluate benthic organisms (on level 3), either all of the "functional feeding groups" (i.e. scrapers, shredders, predators etc. on level 5) should be used concurrently (BioA, BioB), or one (and only one) of the indices (on level 4) that quantify "benthic organisms" (BioPhys, BioB; receiving a weight of 1, respectively). Suitable indices (on level 4) are the "Shannon Weaver index to measure macroinvertebrate diversity", the "Reti-index for benthos", or the "F-13 Yoshimura-index for benthos". If the benthic functional feeding groups are assessed (level 5), they need to be aggregated to level 4. For the expert mean, we used the average of the weights assigned to the different benthos feeding groups by all three experts.

[2] Since only one of the indicators (or the "functional feeding groups") can be chosen, there is no synergy effect.

Level	Sub-objective	Fish	BioA	BioB	BioC	BioPhys	Median
4	*Salmo trutta*	0.31					0.31
4	*Barbus barbus*	0.05					0.05
4	*Chondrostoma nasus*	0.34					0.34
4	*Albumoides bipunctatus*	0.03					0.03
4	No competitors	0.13					0.13
4	No anomalies/injuries	0.15					0.15
Synergy effect		0.25					0.25
5	Total biomass (trout)	0.40					0.40
5	Young of the year (trout)	0.20					0.20
5	Number of juveniles (trout)	0.20					0.20
5	Adult biomass (trout)	0.20					0.20
Synergy effect		0.25					0.25
5	Adult biomass (barbel)	0.60					0.60
5	Young of the year (barbel)	0.20					0.20
5	Number of juveniles (barbel)	0.20					0.20
Synergy effect		0.25					0.25
5	Number of adults (nase)	0.60					0.60
5	Young of the year (nase)	0.40					0.40
Synergy effect		0.25					0.25
5	Dominant species	0.50					0.50
5	Non-specific species	0.50					0.50
Synergy effect		0.25					0.25
