# Supplementary material for: Four Common Simplifications of Multi-Criteria Decision Analysis do not hold for River Rehabilitation
Source: PLoS One. 2016 Mar 8;11(3):e0150695. doi: 10.1371/journal.pone.0150695 (PMC4783037; doi:10.1371/journal.pone.0150695)
Supplement: S2 File — Information from the literature for the attributes A) “diversity of sediment patches”, B) “frequency of floodplain flooding”, C) “substrate clogging”, and D) for “total suspended solids”, translated into value functions (E). (PDF) [file pone.0150695.s002.pdf]

Transforming quality classes from the literature to value functions

We were able to elicit value functions from the interviewed experts in all but five cases. For four of the missing cases, we derived value functions based on the literature: Diversity of patches of river bed for coarse substrate (LAWA, 2000), Frequency of floodplain flooding (LAWA, 2004), Substrate clogging (Schälchli, 2002), and Total suspended solids (Strager et al., 2000; USEPA, 2003). Langhans et al. (2013) propose to translate quality classes for attributes from existing river assessment programs into continuous value functions to comply with utility theory. The authors distinguish between three cases: discrete attributes (i.e. that can only be assessed in classes), not imperatively discrete attributes (i.e. that can be assessed on a continuous scale, but were classified as discrete), and continuous assessments (details see (Langhans et al., 2013; Langhans and Reichert, 2011; Langhans et al., 2014)). For all three cases, one has to first define how to depict the quality classes on the value scale. Depending on the case, different "translations" are proposed. The Swiss assessment procedure (SMC, Swiss Modular Concept of stream assessment (Bundi et al., 2000); http://www.modul-stufen-konzept.ch) uses five quality classes, which (Langhans et al., 2014) interpret as five equidistant intervals on the value scale. Hence, each class (bad, unsatisfactory, moderate, good, and very good) corresponds to a value of 0.2. We used a color-coding to depict all of our value functions following these classes (red, orange, yellow, green, blue). The SMC additionally defines that a sufficiently good state is achieved only in the two upper classes, which corresponds to a minimal value of 0.6. We classified two of our missing attributes as discrete, and two as continuous. The details of the "translations" are given below.

Diversity of patches of river bed (patchdiv)

The attribute "diversity of patches in quality classes" was taken from LAWA (2000). As a first step, the river type needs to be defined. LAWA (2000) allows choosing from 12 different types. The Wigger can be classified as "lowland stream with coarse sediment ("Kiesgewaesser"). Of seven LAWA classes, only five are effectively used for this specific case. Since it was difficult to translate the LAWA-classes into a continuous value function without further information, we used a discrete approach. The discrete approach uses equidistant intervals. We interpreted this in such a way that equal improvements in value (plus 1/ 7 = 0.167) are assigned to each of the seven quality classes. However, only five classes are effectively used and thus "translated" to the value scale (S2 File).

Table A. Value function of diversity of patches of river bed. The translation is based on LAWA (2000) for lowland streams with coarse sediment. The substrate types are: mud/ slurry, clay/ silt (< 0.02 mm), sand (< 2 mm), gravel (2 – 50 mm), cobbles (10 – 30 cm), blocs (> 30 cm), rock, and organic substrate. For explanations, see text.

Diversity of substrate	Description diversity of substrate for a charted river section (LAWA, 2000)	7 LAWA classes (x-axis)	Corresponding values (y-axis)
Very large	Channel bed is characterized by various and strongly changing substrate types. At least three different substrate types occur for at least 2 m^2 (small rivers) or 4 m^2 (medium rivers). Three of these substrate types occur extensively.	1	1
Large	Channel bed is characterized by several distinct changes of the substrate type. At least three different substrate types occur for at least 2 m^2 (small rivers) or 4 m^2 (medium rivers). Two of these substrate types occur extensively.	2	0.833
		(3)	(0.667)

Moderate	Channel bed is characterized by several changes of the substrate type, but the differences between the substrates are mostly small. Three different substrate types occur for at least 2 m^2 (small rivers) or 4 m^2 (medium rivers), but two of these substrate types occur only sporadically and with a low coverage.	4	0.5
Low	Channel bed shows some obvious, but scattered local differences in the substrate type, but overall there are only minor changes.<br><br>Two different substrate types occur for at least 2 m^2 (small rivers) or 4 m^2 (medium rivers), but one of these substrate types occurs only sporadically and with a low coverage.	5<br><br>(6)	0.333<br><br>(0.167)
No diversity	The substrate of the channel bed is totally uniform. Practically only one substrate type occurs over the charted river section.	7	0

Frequency of floodplain flooding (floodplain)

According to LAWA (2004) the frequency of floodplain inundations that are at least two times as large as the river width are assessed. LAWA assigns seven classes, but only three are effectively used for this specific case. This attribute can be considered as continuous. For the translation, we divided the range of the value scale (0 – 1) by the number of classes (1/ 7 = 0.143). For the points on the value scale, we used the borders of the classes, and these points were connected with linear interpolation to create a piecewise linear value function (S2 File).

Table B. Value function of frequency of floodplain flooding. The translation is based on LAWA (2004).

Frequency of floodplain floodings	Classification of river	Original LAWA classes (7)	Values covered	Frequency (floodings/year) (x-axis)	Corresponding values (y-axis)
Every 1 – 2 years	Natural	1	0.857 – 1	1/1 = 1	1
		(2)	0.714 – 0.857	1/2 = 0.5	0.857
Every 3 – 5 years	Impaired	3	0.571 – 0.714	1/3 = 0.333	0.714
		3		1/5 = 0.20	0.571
		(4)	0.429 – 0.571		
		(5)	0.286 – 0.429		
		(6)	0.143 – 0.286		
Fewer than every 5 years	Strongly reduced	7	0 – 0.143	1/6 = 0.167	0.143
				0.000001	0

Substrate clogging (substrclog)

According to Schälchli (2002), the substrate clogging ("innere Kolmation" in German, "colmation") is assessed in five classes. For an English reference also see Woolsey et al. (2007). For the translation to the value scale, the attribute was treated as "discrete" (S2 File).

Table C. Value function of substrate clogging. The translation is based on Schälchli (2002); "Innere Kolmation", Tab. 3, p. 9; also see Woolsey et al., 2007).

Clogging (class)	Description substrate	Description interstitial space	Classes (x-axis)	Corresponding values (y-axis)
None	Substrate very loose and coarsely granular. Only little sand deposits and no cohesive deposits.	Dominantly coarsely porous.	1	1
Weak	Substrate loose and broadly tiered (cobbles, gravel, sand). No cohesive deposits visible (silt, clay).	Coarse to fine porous.	2	0.75
Medium	Substrate slightly cemented. Contact surface filled to ca. ¼ with cohesive fine-particles, rest of the contact surface is mainly sand, but also cobbles and gravel.	To ¾ fine porous, if cohesive deposits, no pores visible.	3	0.5
Strong	Substrate is clearly cemented. Contact surface filled to about one half with fine-particles, rest of contact surface is mainly sand.	Locally still some fine porous spaces visible.	4	0.25
Total	Substrate is strongly cemented. Contact surface is practically continuously filled with cohesive fine-particles.	No interstitial space visible.	5	0

Total suspended solids (sussolidtot)

The value function for total suspended solids was derived from Strager et al. (2000), USEPA (2003), and own assumptions. Since the values are already continuous, they could be directly translated into a value function (S2 File).

Table D. Value function of total suspended solids. The values are based on different literature sources and own estimates.

Total suspended solids (mg/l) as a 30 day average (x-axis)	Corresponding values (y-axis)	Comment to our assumptions and interpretation of the literature data
0	1	
15	0.8	Mid-value between 0 and 30.
30	0.6	Based on different values from USEPA (2003) for cold water streams: Utah, North Dakota, and South Dakota have similar criteria for their cold water streams (p. 20); 35 mg/ l, 30 mg/ l, and 30 mg/ l as a 30 day average or 58 mg/ l daily maximum, respectively. For Canada, clear flow: induced suspended sediment concentrations should not exceed background levels by more than 25 mg/ l during any 24-hour period (p. 26).
115	0.4	Mid-value between 30 and 200.
200	0.2	Based on values from Strager et al. (2000) for „agriculture“ (for “woodland” the value is similar to the US EPA (2003) values of 30 mg/ l above).
500	0	Own estimate.

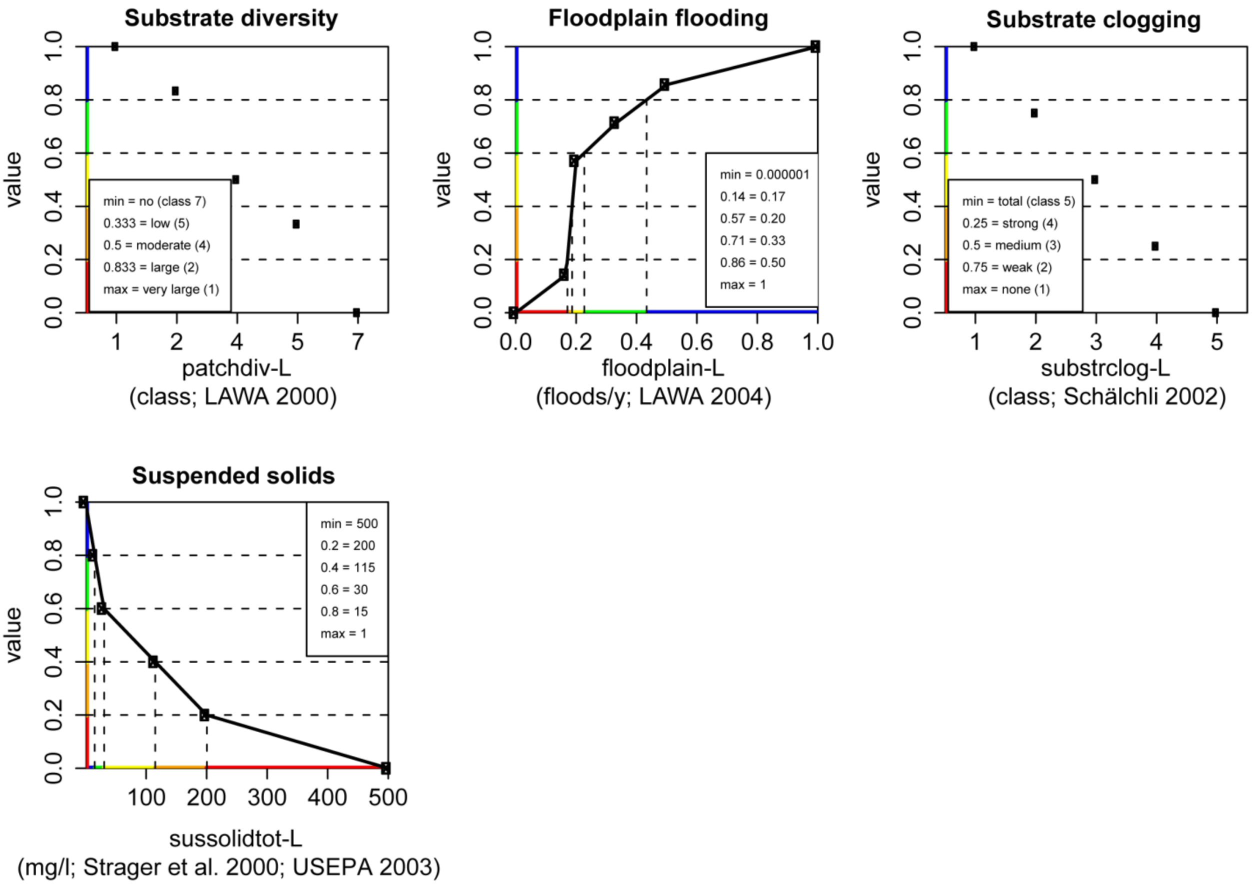

Figure E. Value functions from the literature. For four attributes we derived the value functions from the literature and "translated" them according to Langhans et al. (2013). Explanations see text.

References

Bundi, U., Peter, A., Frutiger, A., Hutte, M., Liechti, P., Sieber, U., 2000. Scientific base and modular concept for comprehensive assessment of streams in Switzerland. Hydrobiologia 422, 477-487.

Langhans, S.D., Reichert, P., 2011. Einbettung von Verfahren zur Fliessgewässerbewertung in ein übergeordnetes Gewässermanagementkonzept. Vorschläge am Beispiel des Modulstufenkonzepts. Wasser Energie Luft 103, 139-148.

Langhans, S.D., Lienert, J., Schuwirth, N., Reichert, P., 2013. How to make river assessments comparable: A demonstration for hydromorphology. Ecological Indicators 32, 264-275.

Langhans, S.D., Schuwirth, N., Reichert, P., 2014. The method matters: guide to indicator aggregation in ecological assessments. Ecological Indicators 45, 494-507.

LAWA, 2000. Gewässerstrukturgütekartierung in der Bundesrepublik Deutschland – Verfahren für kleine und mittelgroße Fließgewässer, Empfehlung. Länderarbeitsgemeinschaft Wasser. http://www.lawa.de/, accessed 22.12.2014

LAWA, 2004. "Gewässerstrukturkartierung in der Bundesrepublik Deutschland - Übersichtsverfahren"; in German (Mapping the quality of water body structures in Germany - Overview procedures), Schwerin, Germany. ISBN 987-3-88961-249-6, http://www.lawa.de/, order number 300823, accessed 22.12.2014.

Strager, M.P., Fletcher, J.J., Yuill, C.B., Strager, J.M., 2000. Not in My Watershed! An Interactive Tool to Evaluate Land Use Changes on Stream Water Quality, Prodeedings of the ESRI International User Conference.

http://proceedings.esri.com/library/userconf/proc00/professional/papers/PAP663/p663.htm, accessed 22.12.2014.

USEPA, 2003. Developing water quality criteria for suspended and bedded sediments (SABS), Potential approaches, US EPA Office of Water, Office of Science and Technology, Draft, August 2003. http://water.epa.gov/scitech/swguidance/standards/criteria/aqlife/pollutants/sediment/upload/2004_08_17_criteria_sediment_sab-discussion-paper.pdf, accessed 22.12.2014.

Woolsey, S., Capelli, F., Gonser, T., Hoehn, E., Hostmann, M., Junker, B., Paetzold, A., Roulier, C., Schweizer, S., Tiegs, S.D., Tockner, K., Weber, C., Peter, A., 2007. A strategy to assess river restoration success. Freshwater Biology 52, 752-769.
