# Supplementary material for: Four Common Simplifications of Multi-Criteria Decision Analysis do not hold for River Rehabilitation
Source: PLoS One. 2016 Mar 8;11(3):e0150695. doi: 10.1371/journal.pone.0150695 (PMC4783037; doi:10.1371/journal.pone.0150695)
Supplement: S3 File — Objectives hierarchies identified by the A) Fish-, B) BioA-, C) BioB-, D) BioC-, E) BioPhys-, and the F) Phys-expert. (PDF) [file pone.0150695.s003.pdf]

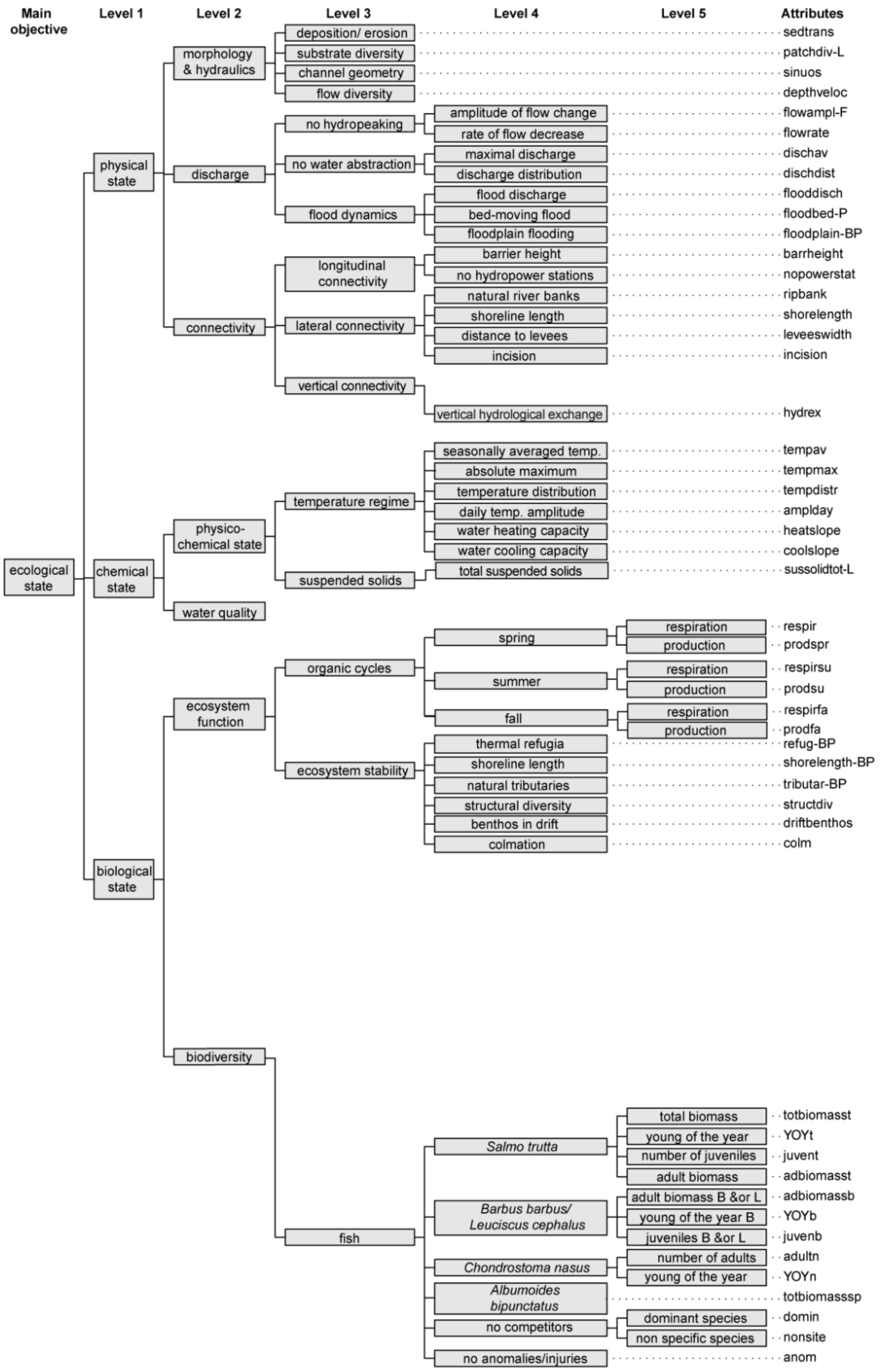

Figure A. Objectives hierarchy showing all sub-objectives defined as essential by the Fish-expert.

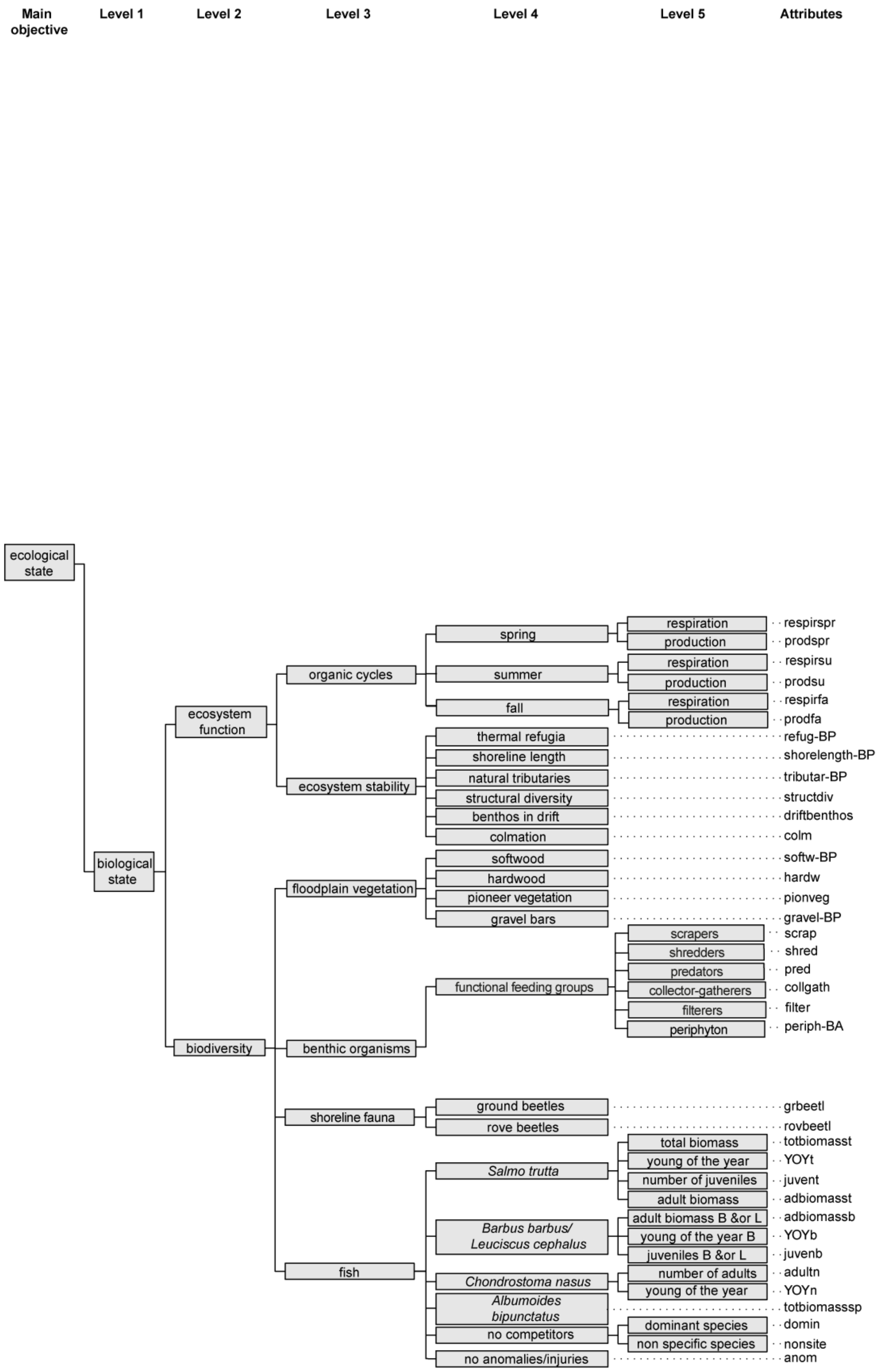

Figure B. Objectives hierarchy showing all sub-objectives defined as essential by the BioA-expert.

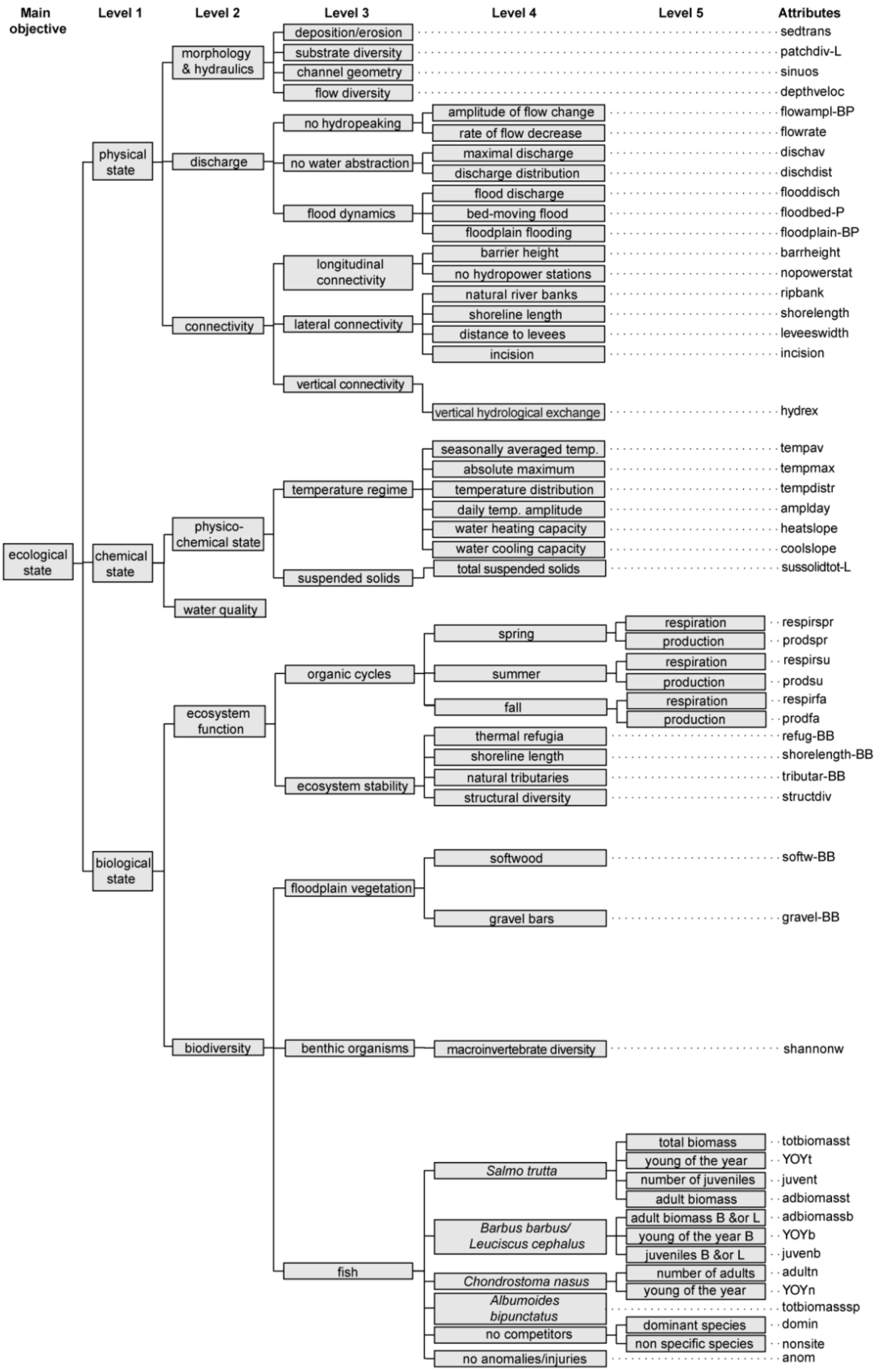

Figure C. Objectives hierarchy showing all sub-objectives defined as essential by the BioB-expert.

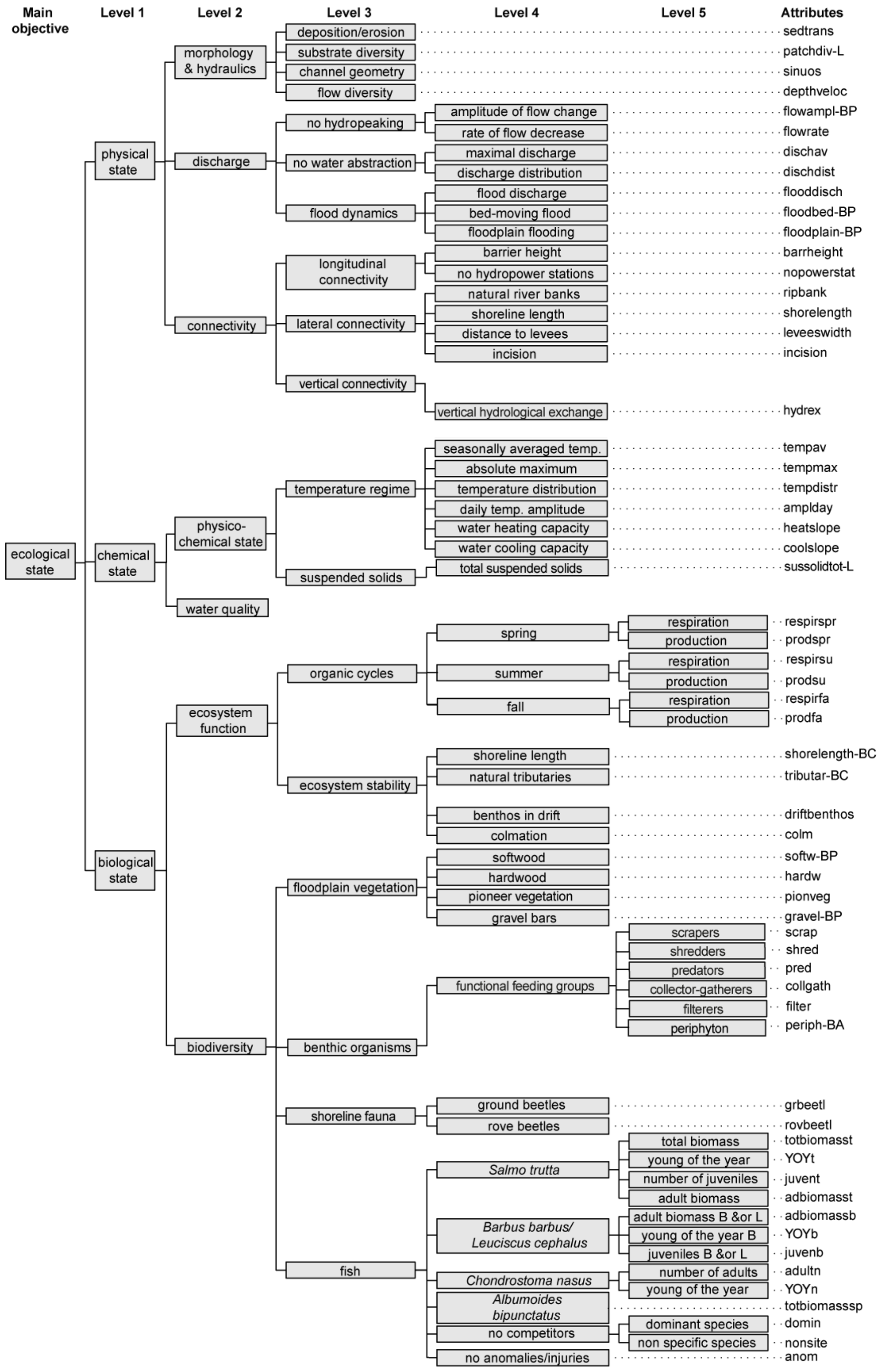

Figure D. Objectives hierarchy showing all sub-objectives defined as essential by the BioC-expert.

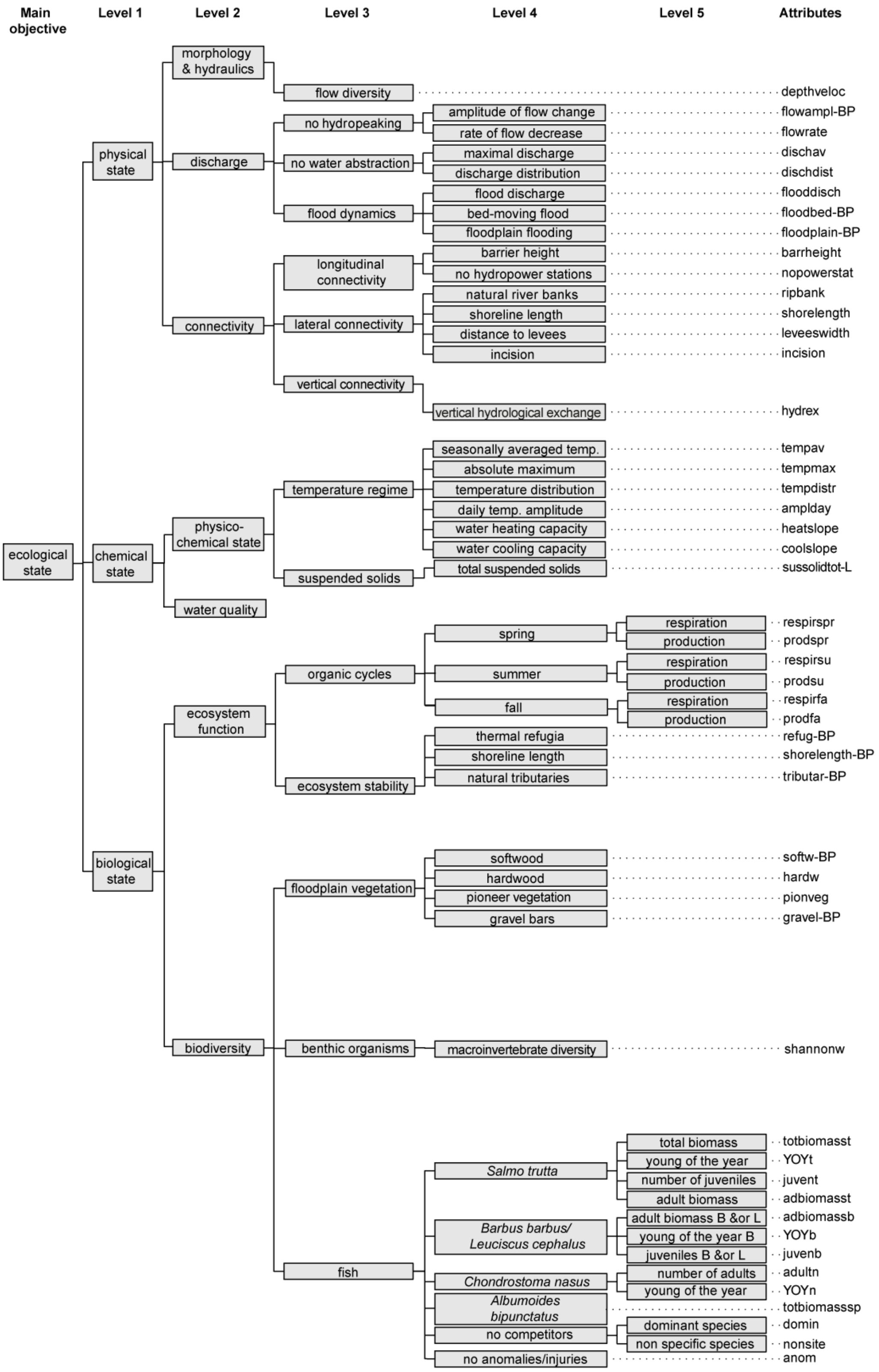

Figure E. Objectives hierarchy showing all sub-objectives defined as essential by the BioPhys-expert.

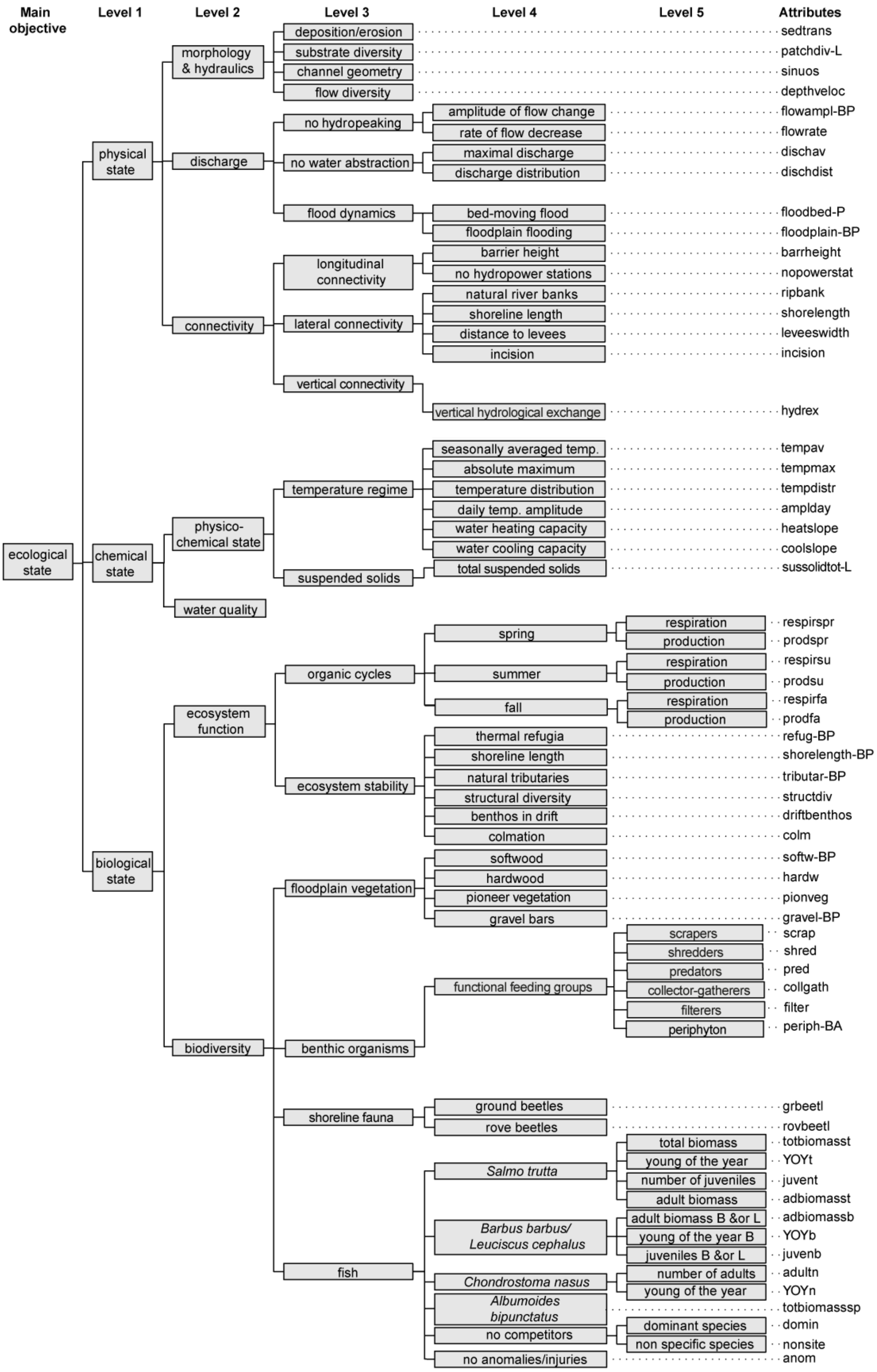

Figure F. Objectives hierarchy showing all sub-objectives defined as essential by the Phys-expert.
