# Supplementary material for: Four Common Simplifications of Multi-Criteria Decision Analysis do not hold for River Rehabilitation
Source: PLoS One. 2016 Mar 8;11(3):e0150695. doi: 10.1371/journal.pone.0150695 (PMC4783037; doi:10.1371/journal.pone.0150695)
Supplement: S4 File — A) Aggregation methods for value functions, and B) synergy effect for multiplicative aggregation. (PDF) [file pone.0150695.s004.pdf]

Discussion of aggregation in first interviews

We hypothesize that an additive value function might not be appropriate for ecological objectives. To prepare the second interview, we discussed this already in the first interview. First, we explained that we are dealing with a multi-dimensional space consisting of 20 or more attributes and that we have to come up with an aggregated judgment to assess the overall ecological state of a river. We asked, what kind of indices or other aggregations of individual assessments were known to the expert and what his/ her opinion was concerning these.

We then proposed a hierarchical aggregation procedure, moving upwards in the objectives hierarchy: First, the attributes within each sub-objective are aggregated. The sub-objectives are then aggregated to the next-higher objectives. This is repeated for each branch and level of the hierarchy until the uppermost objective “high level of ecological integrity” is reached. We discussed the advantages of this procedure (see main text) and asked whether the experts agreed or had other ideas.

Third, we discussed mathematical aggregations and their implications. We also used two diagrams to visualize different aggregation methods. Since the interviewees were scientists, they well understood this. We discussed the implications of the additive, multiplicative, and minimum aggregation, but not of the cobb-douglas and mixed forms. After having discussed the different procedures in detail, we asked whether the weighted mean (= additive aggregation) is a reasonable assumption and in which cases it might be problematic. If it was regarded as problematic, we asked whether the minimal requirement or a multiplicative aggregation might be more appropriate, and if yes, systematically moving through the objectives hierarchy, for which objectives/ attributes.

The general explanation for the different aggregation methods in words was as follows:

(1) Additive aggregation: “One possibility to aggregate information is to calculate the weighted mean of the values associated with each attribute. This implies that deficits in some sub-objectives can be compensated by good scores in other sub-objectives. As example, in the Swiss Modular Concept for stream assessment (Bundi et al., 2000), seven objectives are required to assess the “natural diversity and abundance of fish”, including “natural species composition”, “natural density of brown trout” and “no deformations or anomalies”. Imagine that you find each of these goals equally important, so they receive equal weights (1/ 7 = 0.14). Further imagine that you are assessing a river stretch, where goals 1 – 6 are fully achieved (value = 1), but you find deformations in nearly every fish (value = 0). This results in a weighted mean of 0.86 (= 0.14 * 6). On a scale of 0 – 1, achieving 0.86 is a high value, meaning that in this river stretch you have reached 86% of your goals with respect to fish, even though each fish has deformations. Hence, using a weighted sum allows for trade-offs (a very poor fulfilment of one goal can be compensated by a very high fulfilment of another). Additionally, if many attributes or sub-objectives are integrated, a single one will have only a small influence on the overall evaluation, as in our example. This could be partially compensated by giving “anomalies” a higher weight than the others. However, this would be problematic if the same effect occurs for another goal (e.g. no brown trout in the river).”

“As second example with two attributes (with equal weights), additivity would mean that you would judge the following three states of the river as equivalent: (a) attribute A has ecological state of 0 and attribute B has ecological state of 1; (b) attribute B has ecological state of 0 and attribute A of 1; and (c) both attributes have ecological state of 0.5.”

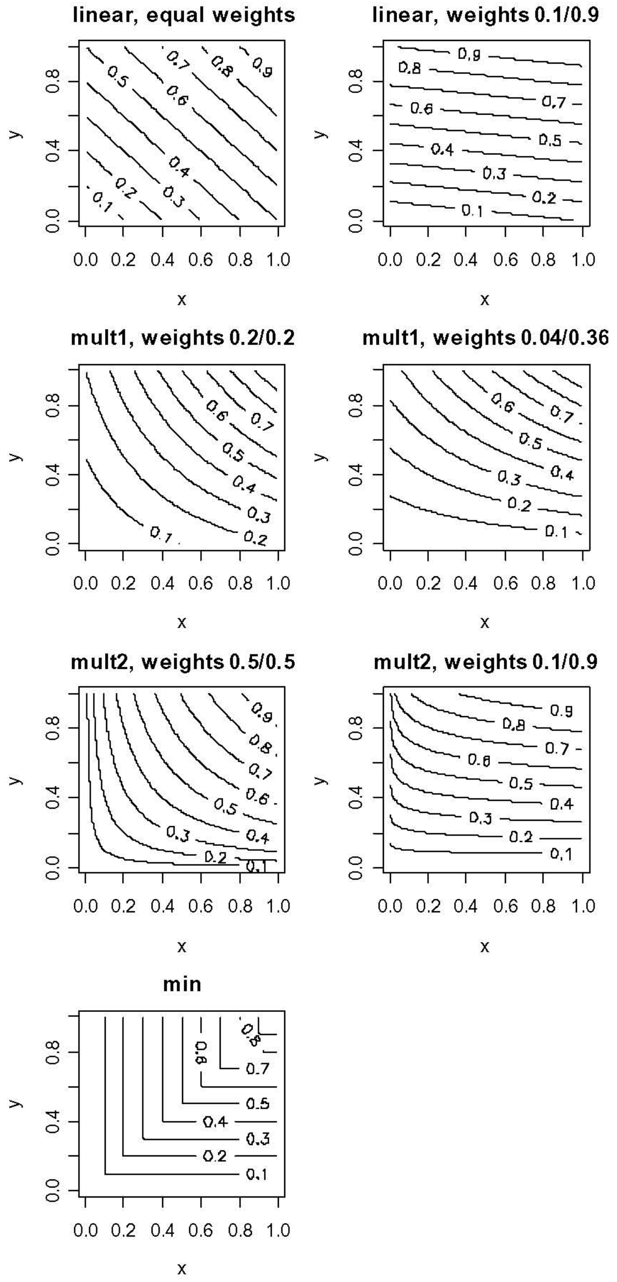

Figure A. Aggregation methods for value functions. Diagram used in the first interviews to discuss the implications of different aggregation methods. We used the example of two attributes x, y with the single-attribute values on a scale of 0 (worst possible state) to 1 (best possible state). The isolines show the aggregated value of the higher-level objective for different aggregation methods and weights assigned to x and y: linear (equal weights/ weights of 0.1 and 0.9), multiplicative (different weights that need not sum up to 1), and minimal requirement.

(2) Minimal requirement: "We might define as conditional requirement that all sub-objectives should be fulfilled to at least a minimum level for achieving a good score for a certain overall state. As example, a minimal level of "no deformations or anomalies" is required to achieve a good level of "natural diversity and abundance of fish". So in this case the overall valuation can never be better than the valuation of the worst attribute."

(3) Multiplicative aggregation: "Compare the above example for two attributes (with equal weights) with this outcome: You would judge the ecological state of a river of higher value if both attributes have a medium value of 0.5. The ecological state of a river would be judged as lower, if one attribute

is on its best level (value = 1), while the other is on its worst (value = 0). This means that the multiplicative aggregation entails a preference of having e.g. all functional groups in an ecosystem to be present and in a relatively good state, i.e. you do not want to have one missing or in a bad state, even if the other functional groups are present in a good state. The valuation is not independent for each attribute/ objective; rather there is an added value or synergy of an integral consideration of all of them together."

Methods: aggregation schemes

To aggregate lower-level values to the next-higher node, and culminating in the overall value v(a) for the objective "high level of ecological integrity", we used the following aggregation schemes:

The additive model

For the additive model (Dyer and Sarin, 1979; Eisenführ et al., 2010; Keeney and Raiffa, 1976), one has to know the single-attribute value functions v_i (elicited in first interview) and the weights w_i (elicited in second interviews) that sum up to one. The additive model requires measurable/ cardinal value functions and strong independence conditions (mutual preference independence, difference independence), which are not necessarily fulfilled. Implications are that the decision makers' preferences for certain outcomes (entailing certain attribute levels) do not depend on the levels of other attributes. Another important implication of the additive model is that a low value in one attribute can be compensated by a high value in a second attribute. The formula is:

$$v(a)_{add} = \sum_{i=1}^{m} w_i v_i(a_i) \tag{1}$$

$v(a)_{add}$	=	total value of alternative *a* using additive aggregation
a_i	=	attribute level of alternative *a* for attribute I
$v_i(a_i)$	=	value for attribute *i* of alternative *a*
w_i	=	scaling constant (weighting factor) of attribute *i*; $\sum w_i = 1$

Minimum aggregation

In this model, the overall value of an alternative *v(a)* equals the worst value of a single attribute; i.e. the value of the remaining (better-performing) attributes do not enter the overall assessment:

$$v(a)_{\min} = \min(v_i(a_i)) \tag{2}$$

The multiplicative model

The multiplicative aggregation model requires weaker preference independence conditions than the additive model, which is why it is the popular (but rarely applied) alternative to the additive model in the decision analysis literature ((Dyer and Sarin, 1979; Keeney and Raiffa, 1976) and textbooks e.g. (Eisenführ et al., 2010)). Exceptions using multiplicative models are e.g. Delforce and Hardaker (1985), Duckstein et al. (1994), Keeney and Wood (1977), Raju and Pillai (1999), Raju and Vasan (2007), and Torrance et al. (1996). However, it requires elicitation of an additional parameter. It is thus based on the single-attribute value functions v_i (elicited in the first interview), and two scaling constants (elicited in the second interviews), the weights w_i, and an additional synergy factor k, which

accounts for the added value of having all attributes on a similarly good level. If k = 1, we are back to the additive model. The formula is:

$$v(a)_{mult} = \frac{\prod_{i=1}^{m}\left[kw_i v_i(a_i) + 1\right] - 1}{k} \tag{3}$$

The advantage of weaker preference conditions may be counter balanced by the complexity of the model and the necessity to elicit the additional scaling constant k. Both may be very difficult to convey to decision makers (especially if they are not natural scientists). Additionally, the multiplicative model has the strange property that in the special case where all attributes have the same value $v(a_i)$, the overall (aggregated) value $v(a)_{mult}$ is lower than each single-attribute value. How much lower depends on the convexity of the curve (see the indifference curves for two attributes in Figure A). The scaling constant (also termed interaction constant) k only has a normalizing role that guarantees that the function $v(a)_{mult}$ remains between 0 and 1. k is calculated as:

$$1 + k = \prod_{i=1}^{m}(1 + kw_i) \tag{4}$$

Synergy effect

In the second interview series, we discussed in detail the synergy effect when using multiplicative aggregation with our interview partners, along the following lines: When aggregating objectives (or attributes) there are two degrees of freedom. The first allows assigning different weights for the objectives. As second degree of freedom, it is possible to define how much importance is assigned to synergistic effects, when increasing several objectives simultaneously.

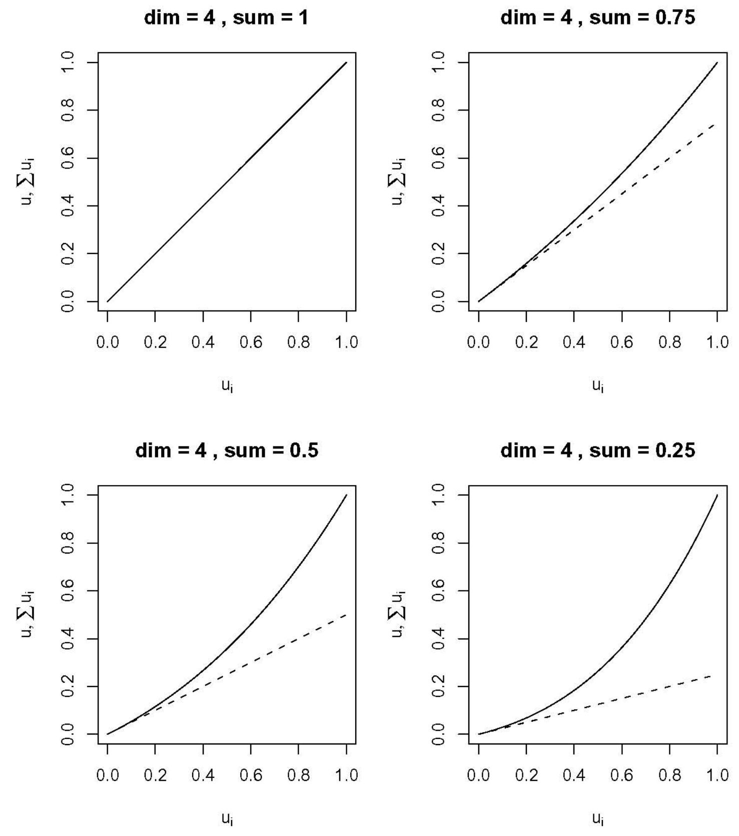

Figure B. Synergy effect for multiplicative aggregation. Diagram used in the second interviews to discuss the implications of multiplicative aggregation. We used the example of four objectives (dim = 4) with equal weights. These objectives can achieve different values between 0 (worst-possible state) and 1 (best-possible state; μ on x-axis). On the y-axis, the aggregated value is shown, i.e. after aggregating the single values of each objective (x-axis) to the next-higher level. Solid line: all objectives are increased from a value of 0 (worst-possible state on x-axis) to a value of 1 (best-possible state) together, using multiplicative aggregation. Dashed line: the value of only one objective at a time is increased from 0 to 1, i.e. this is done for each objective on after the other, and the resulting values are then summed up.

- Top-left: Additive = multiplicative case, sum of weights equals 1 (i.e. 0.25 for each objective)
- Top-right: Multiplicative with small synergy effect of having all objectives on a similarly good level, where sum of weights = 0.75 (i.e. 0.1875 for each objective)
- Bottom-left: Multiplicative with medium synergy effect, sum of weights = 0.5 (i.e. 0.125 for each objective)
- Bottom-right: Multiplicative with large synergy effect, sum of weights = 0.25 (i.e. 0.0625 for each objective)

In the case of additive aggregation, the objectives are independent. The improvement of one objective can compensate for a bad state of another attribute (top-left in Figure B). However, if multiplicative aggregation is used, a better overall value is achieved if all objectives are increased together (solid lines top-right and bottom in Figure B) than if only one individual objective at a time is improved, but not the others (dashed lines Figure B). In other words: “improving all objectives together” (solid line) is better than the sums of individual improvements (dashed lines). Additionally, it is possible to define how important this synergistic effect of having all attributes increased together is (large, medium, or small, Figure B).

As example, Figure B top right shows multiplicative aggregation with a small synergistic effect (sum of weights = 0.75; solid line). The difference between the dashed line (improvement of one objective to value of 1; all others remain at value of 0; repeated for the other three objectives and summed up) and the solid line (improvement of all objectives together) is small: There is not much difference between the overall values (solid and the dashed line).

The cobb-douglas model

For the sake of completeness, we also include the properties of the cobb-douglas and mixed models, here, even though they were not used in this analysis.

This model was developed as a measure for the productivity of labour (Cobb and Douglas, 1928) and has been applied to many other contexts. It is also used as a utility (or value) function for preferences ((Varian, 2010); in the economics literature, "utilities" are used as synonym for the term "values" as used here, following the decision analysis literature). Again, the single-attribute value functions $v_i(a_i)$ and the scaling constants (weights) w_i are elicited from the decision maker. As convention, the sum of the weights equals 1. The cobb-douglas function has similarly convex indifference curves as the multiplicative function, and the property, that the total value $v(a)_{cd}$ equals zero if any value for a single attribute $v_i(a_i)$ is zero. The formula is:

$$v(a)_{cd} = \prod_{i=1}^{m} v_i(a_i)^{w_i} \tag{5}$$

It is equivalent to the weighted geometric mean, which is well known in AHP (Saaty, 1977), where it is the most common method for aggregating group opinions (e.g., (Xu, 2000)).

The mixed model

To sum it up, all above aggregation methods have at least one undesired property: The additive method allows deficits in some sub-objectives being compensated by good scores in other sub-objectives. Minimum aggregation is prone to the pessimism bias (Cunningham, 2012), i.e. reporting a too pessimistic ecological status. In the multiplicative aggregation function, the total value $v(a)$ equals zero, if any value for a single attribute $v_i(a_i)$ is zero. Additionally, it aggregates attributes with the same value $v(a_i)$ into a lower value $v(a)$ than each single-attribute value.

To overcome these deficiencies, a mixed model has been proposed that combines the additive and minimum aggregation with the cobb-douglas model. It requires elicitation of the same parameters from the decision maker as the additive model, namely the single-attribute value functions $v_i(a_i)$ and the scaling constants (or weights) w_i. To specify the relative contributions each aggregation function to the overall model, the factors α (for the contribution of the additive model), β (minimum) and γ (cobb-douglas) are introduced. As example, if β and γ are zero, we are back to the additive model. α, β, and γ should sum up to unity. The resulting indifference curves of the mixed model are also convex as in the multiplicative and cobb-douglas function, but without the overall value $v(a)_{mix}$ approaching zero if the value of a single attribute is zero (i.e. this is the contribution of the additive model and overcomes the problem of the cobb-douglas aggregation); and without the disadvantage of the overall value $v(a)_{mix}$ being lower than $v_i(a_i)$ in the special case of equal single-attribute values (i.e. the disadvantage of the multiplicative model). The function is:

$$v(a)_{mix} = \alpha \sum_{i=1}^{m} w_i v_i(a_i) + \beta \min(v_i(a_i)) + \gamma \prod_{i=1}^{m} v_i(a_i)^{w_i} \qquad (6)$$

Another proposition is to mix the additive model with minimum (or maximum) aggregation to compensate for the undesired properties of the additive model alone. The advantages of this approach are discussed in detail by Langhans et al. (2014).

References

Bundi, U., Peter, A., Frutiger, A., Hutte, M., Liechti, P., Sieber, U., 2000. Scientific base and modular concept for comprehensive assessment of streams in Switzerland. Hydrobiologia 422, 477-487.

Cobb, C.W., Douglas, C.H., 1928. A theory of production. American Economic Review 18, 139-165.

Cunningham, R., 2012. Glass half full or half empty? Why 2009 Water Framework Directive classification results are over-optimistic about the state of rivers despite the One-out, All-out rule. Royal Society for the Protection of Birds. p. 11.

Delforce, R.J., Hardaker, J.B., 1985. An experiment in multiattribute utility-theory. Australian Journal of Agricultural Economics 29, 179-198.

Duckstein, L., Treichel, W., Elmagnouni, S., 1994. Ranking groundwater-management alternatives by multicriterion analysis. Journal of Water Resources Planning and Management-Asce 120, 546-565.

Dyer, J.S., Sarin, R.K., 1979. Measurable multiattribute value functions. Operations Research 27, 810-822.

Eisenführ, F., Weber, M., Langer, T., 2010. Rational Decision Making. Springer-Verlag, Berlin, Heidelberg.

Keeney, R.L., Raiffa, H., 1976. Decisions with multiple objectives: preferences and value tradeoffs. Cambridge University Press, Cambridge, United Kingdom.

Keeney, R.L., Wood, E.F., 1977. Illustrative example of use of multiattribute utility theory for water-resource planning. Water Resources Research 13, 705-712.

Raju, K.S., Pillai, C.R.S., 1999. Multicriterion decision making in performance evaluation of an irrigation system. European Journal of Operational Research 112, 479-488.

Raju, K.S., Vasan, A., 2007. Multi attribute utility theory for irrigation system evaluation. Water Resources Management 21, 717-728.

Saaty, T.L., 1977. A scaling method for priorities in hierarchical structures. Journal of Mathematical Psychology 15, 234-281.

Torrance, G.W., Feeny, D.H., Furlong, W.J., Barr, R.D., Zhang, Y.M., Wang, Q.N., 1996. Multiattribute utility function for a comprehensive health status classification system - Health Utilities Index Mark 2. Medical Care 34, 702-722.

Varian, H.R., 2010. Intermediate microeconomics: a modern approach. W.W. Norton and Company, New York.

Xu, Z.S., 2000. On consistency of the weighted geometric mean complex judgement matrix in AHP. European Journal of Operational Research 126, 683-687.
