# Supplementary material for: Four Common Simplifications of Multi-Criteria Decision Analysis do not hold for River Rehabilitation
Source: PLoS One. 2016 Mar 8;11(3):e0150695. doi: 10.1371/journal.pone.0150695 (PMC4783037; doi:10.1371/journal.pone.0150695)
Supplement: S5 File — (PDF) [file pone.0150695.s005.pdf]

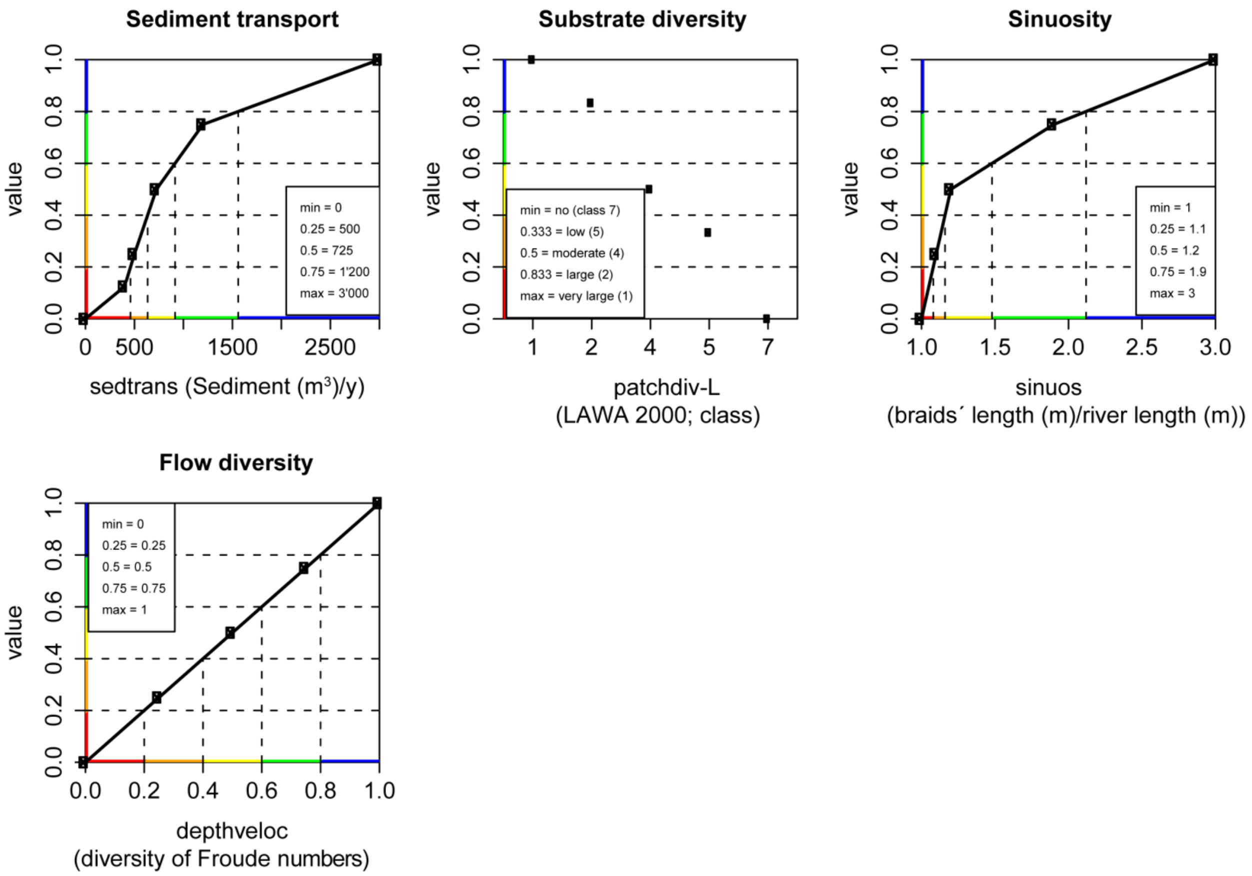

Figure A. Value functions natural river morphology and hydraulics. Value functions for the attributes of a braiding river of “natural river morphology and hydraulics”/ “natural deposition and erosion”, “high diversity of substrate of river bed”, “natural channel geometry” and “natural flow diversity”, elicited from the experts Phys, BioPhys, and the literature. Details see S2 and S3 Tables and text.

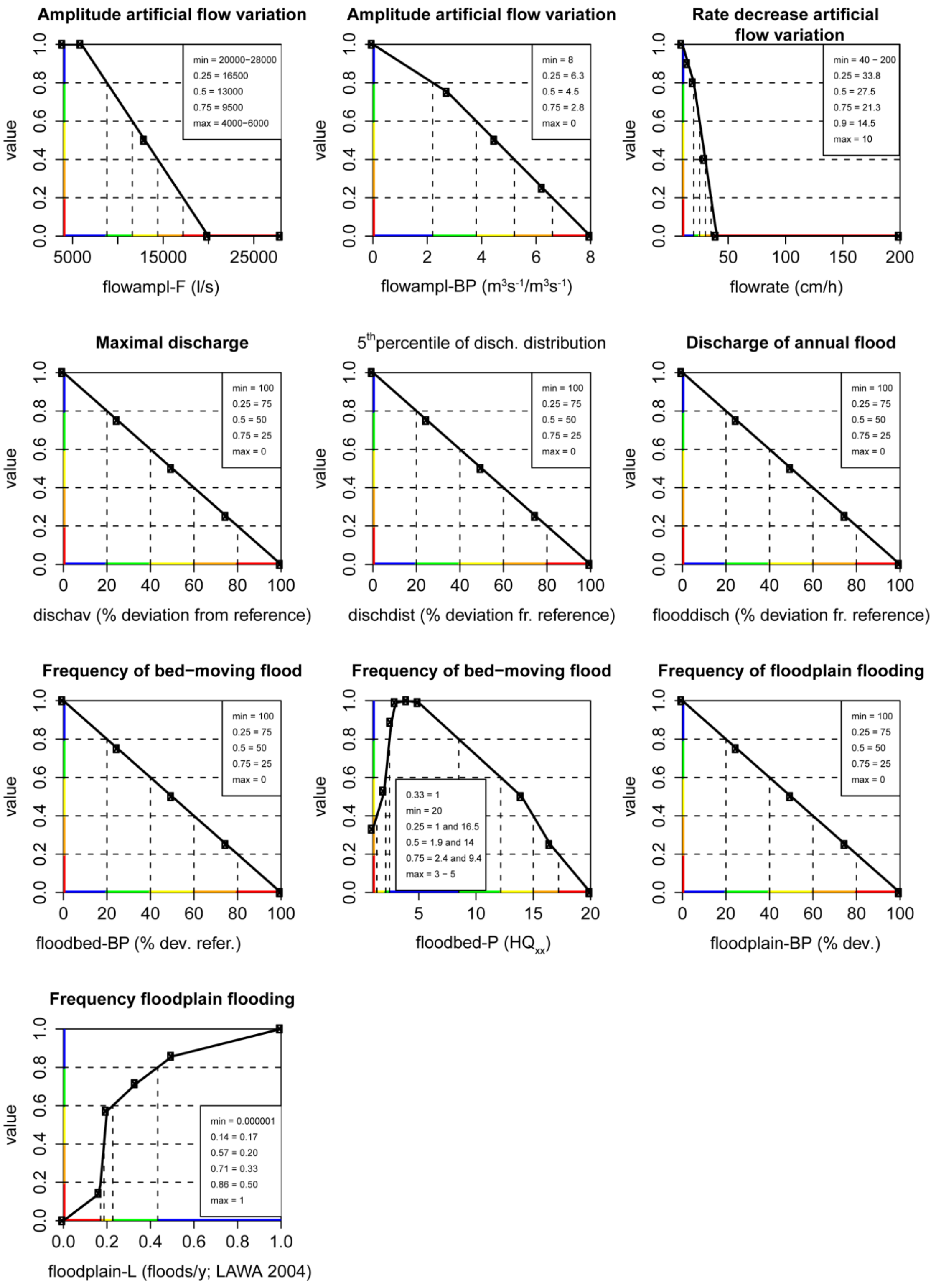

Figure B. Value functions natural discharge regime. Value functions for the attributes of "natural discharge regime"/ "no hydropeaking", "no water abstraction" and "natural flood dynamics", elicited from the experts Fish, BioPhys, Phys, and the literature. Details see S2 and S3 Tables and text.

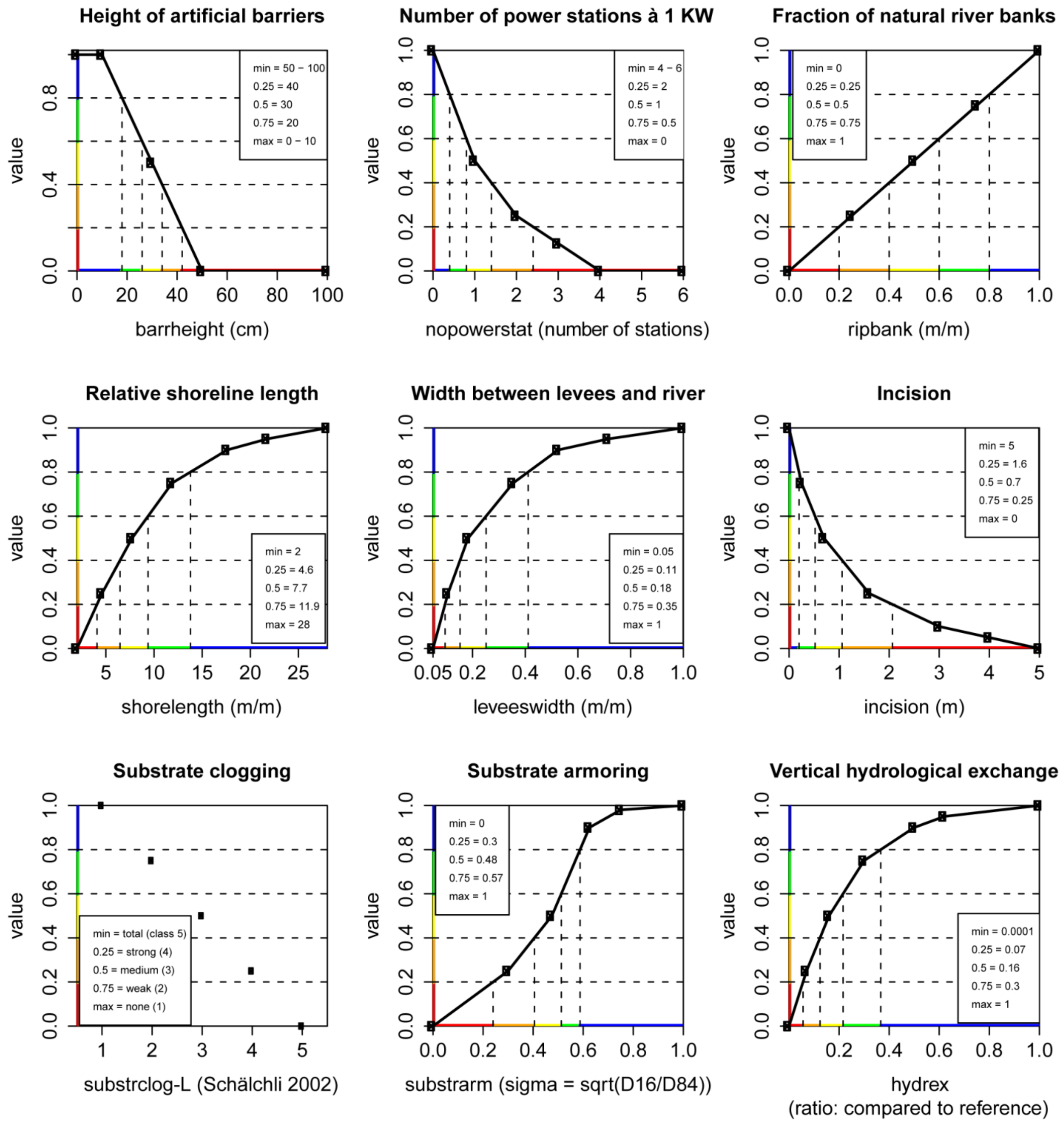

Figure C. Value functions good connectivity. Value functions for the attributes of "good connectivity"/ "high longitudinal connectivity", "high lateral connectivity" and "high vertical connectivity", elicited from Fish, BioPhys, Phys, and the literature. Details see S2 and S3 Tables and text.

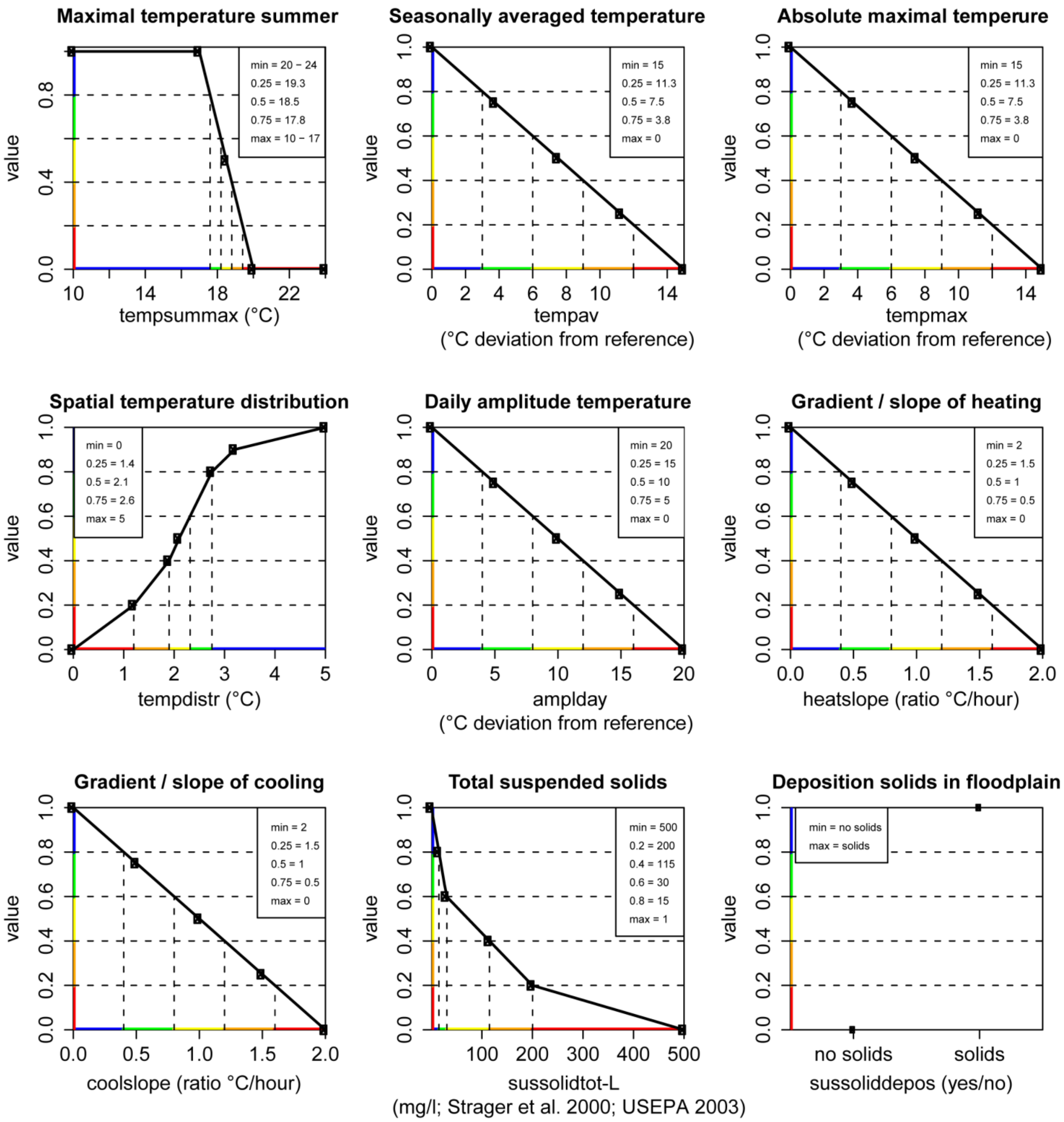

Figure D. Value functions physico-chemical quality. Value functions for the attributes of "high physico-chemical quality"/ "natural temperature regime" and "natural level of suspended solids, elicited from the experts Fish, and BioPhys, BioC, and the Literature. Details see S2 and S3 Tables and text.

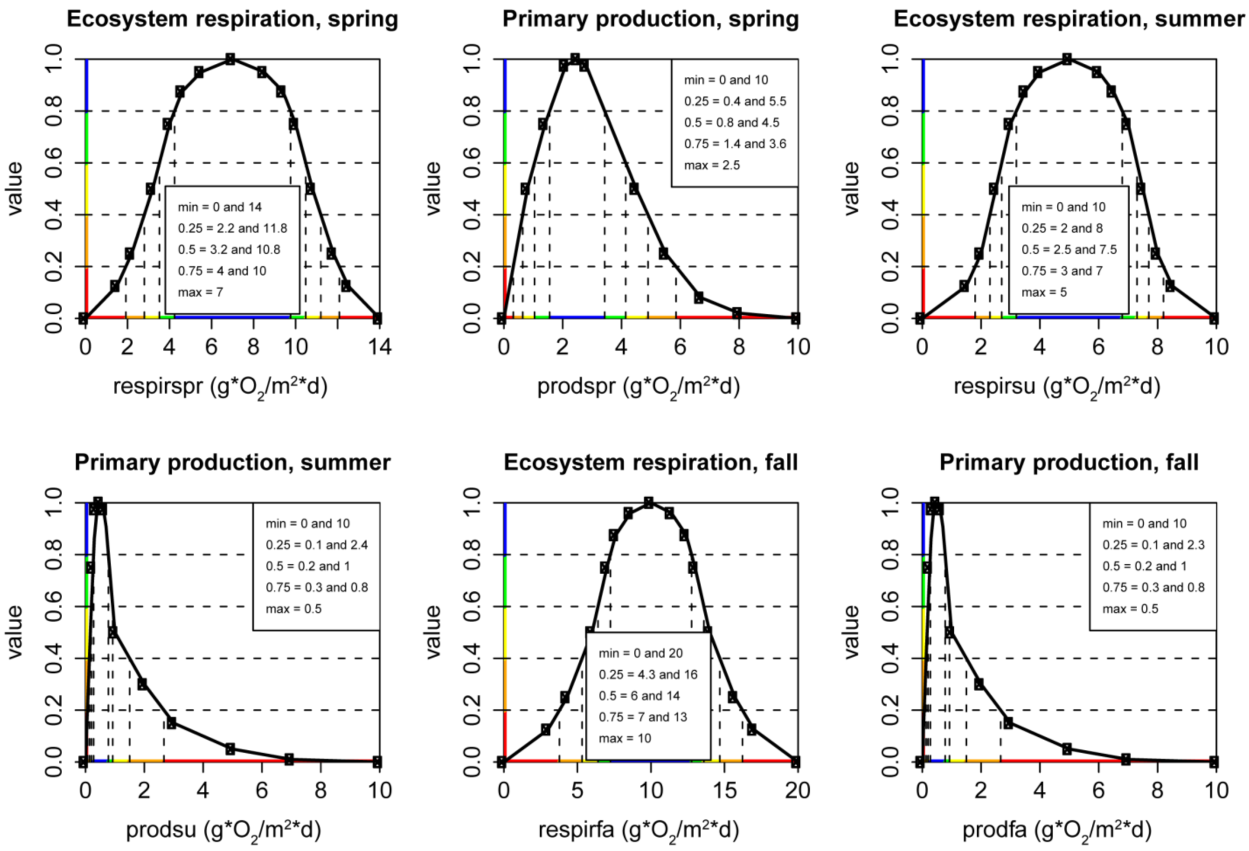

Figure E. Value functions functioning organic cycles. Value functions for the attributes of "natural ecosystem function"/ "functioning organic cycles", elicited from the BioPhys-expert. Details see S2 and S3 Tables and text.

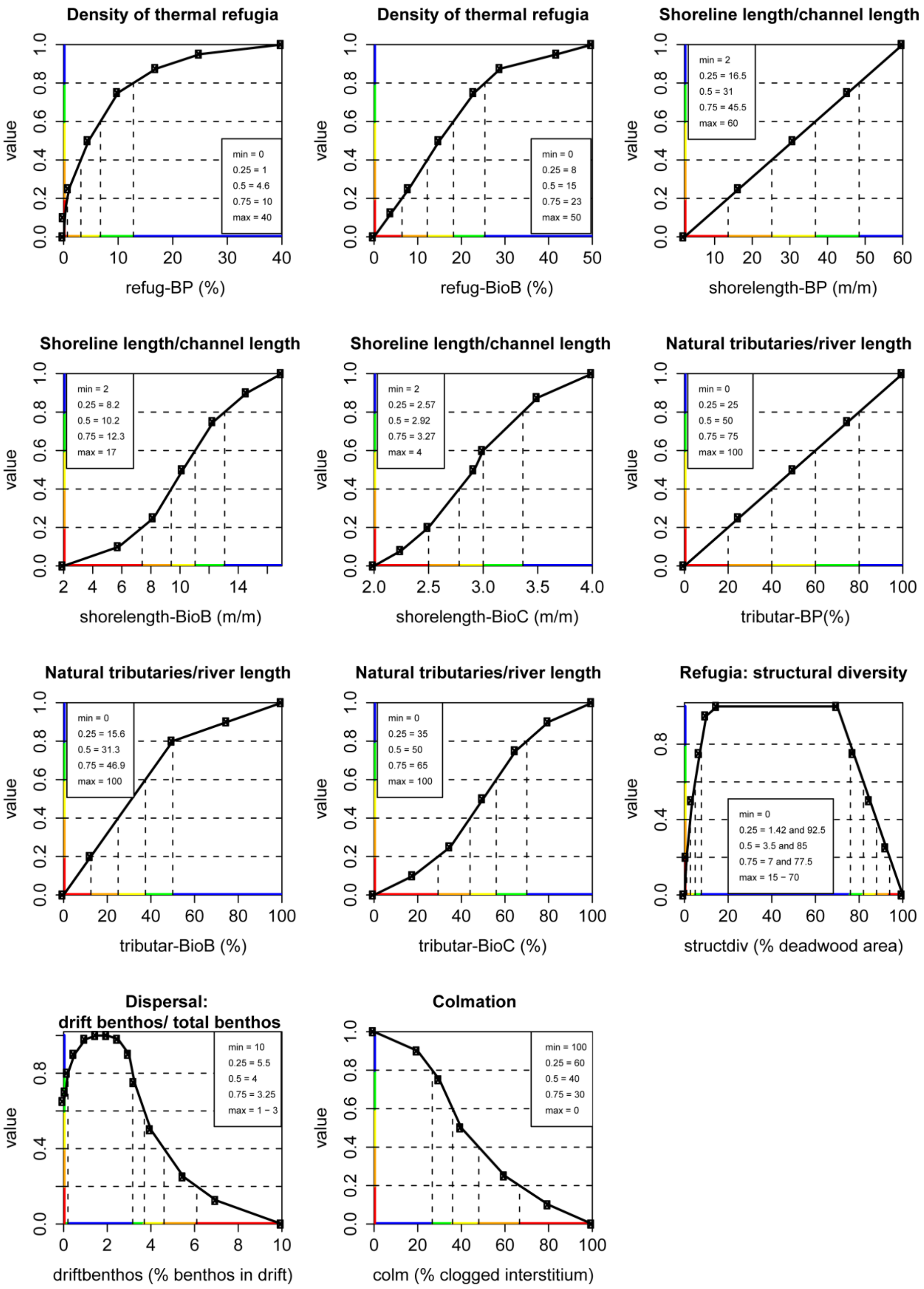

Figure F. Value functions ecosystem stability. Value functions for the attributes of "natural ecosystem function"/ "ecosystem stability", elicited from the experts BioB, BioC, and BioPhys. Details see S2 and S3 Tables and text.

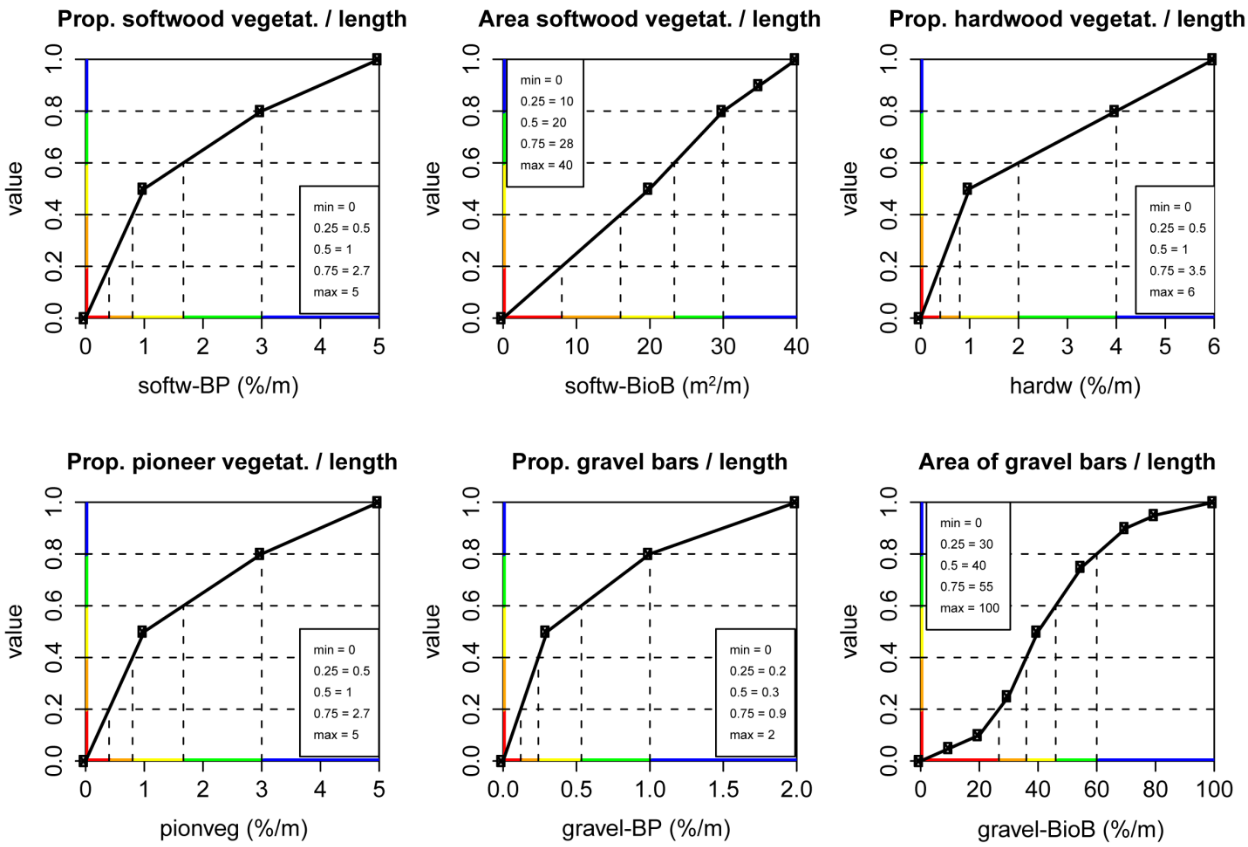

Figure G. Value functions floodplain vegetation. Value functions for the attributes of “natural diversity”/ “natural floodplain vegetation”, elicited from the experts BioPhys and BioB. Details see S2 and S3 Tables and text.

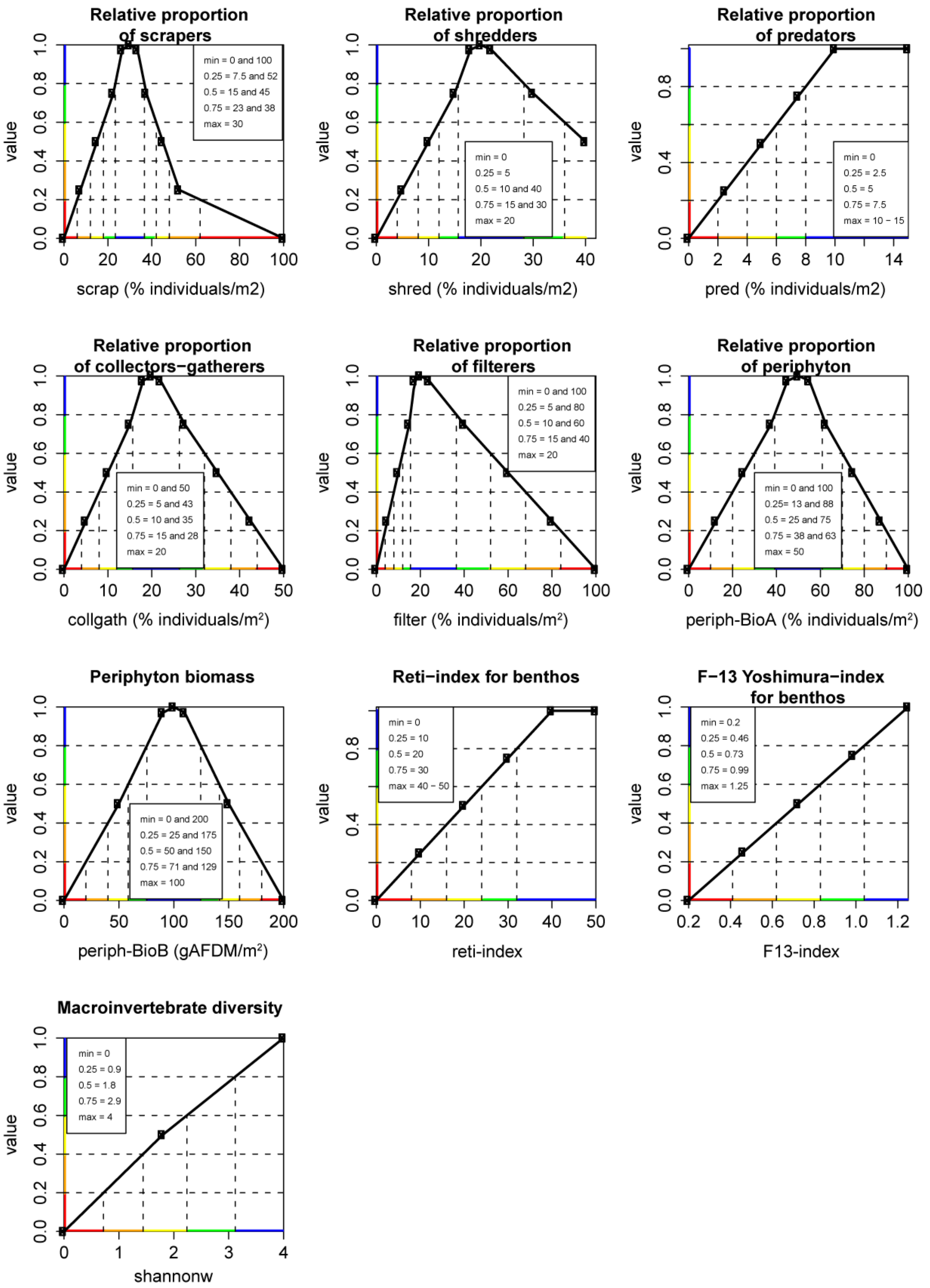

Figure H. Value functions benthos. Value functions for the attributes of “natural diversity”/ “natural benthic organisms”, elicited from the experts BioA, BioB, and BioPhys. Details see Tabs. S9 and S10 and text.

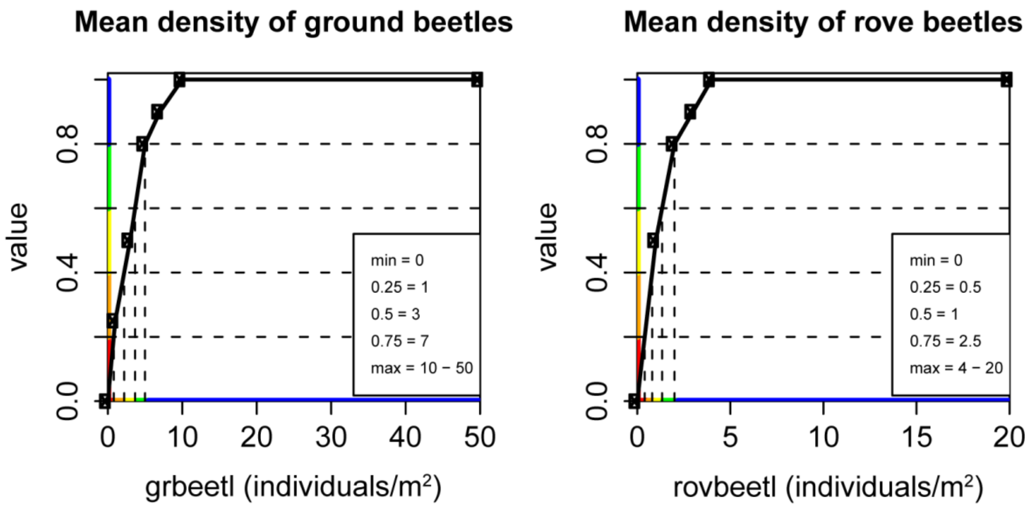

Figure I. Value functions shoreline fauna. Value functions for the attributes of “natural diversity”/ “natural shoreline fauna”, elicited from the BioA-expert. Details see S2 and S3 Tables and text.

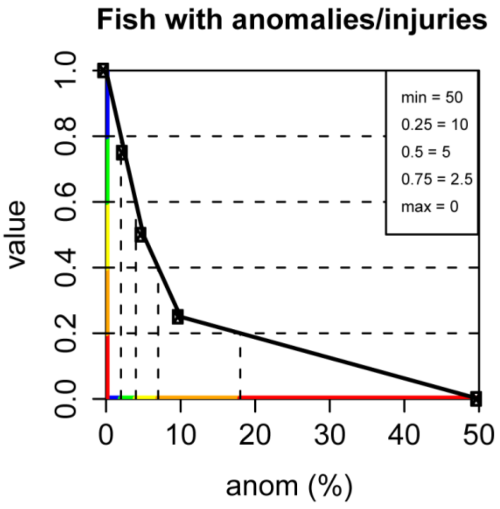

Fig. J. Value functions fish. Value function for the attribute of “natural fish diversity” elicited from the Fish-expert. Details see S2 and S3 Tables and text.

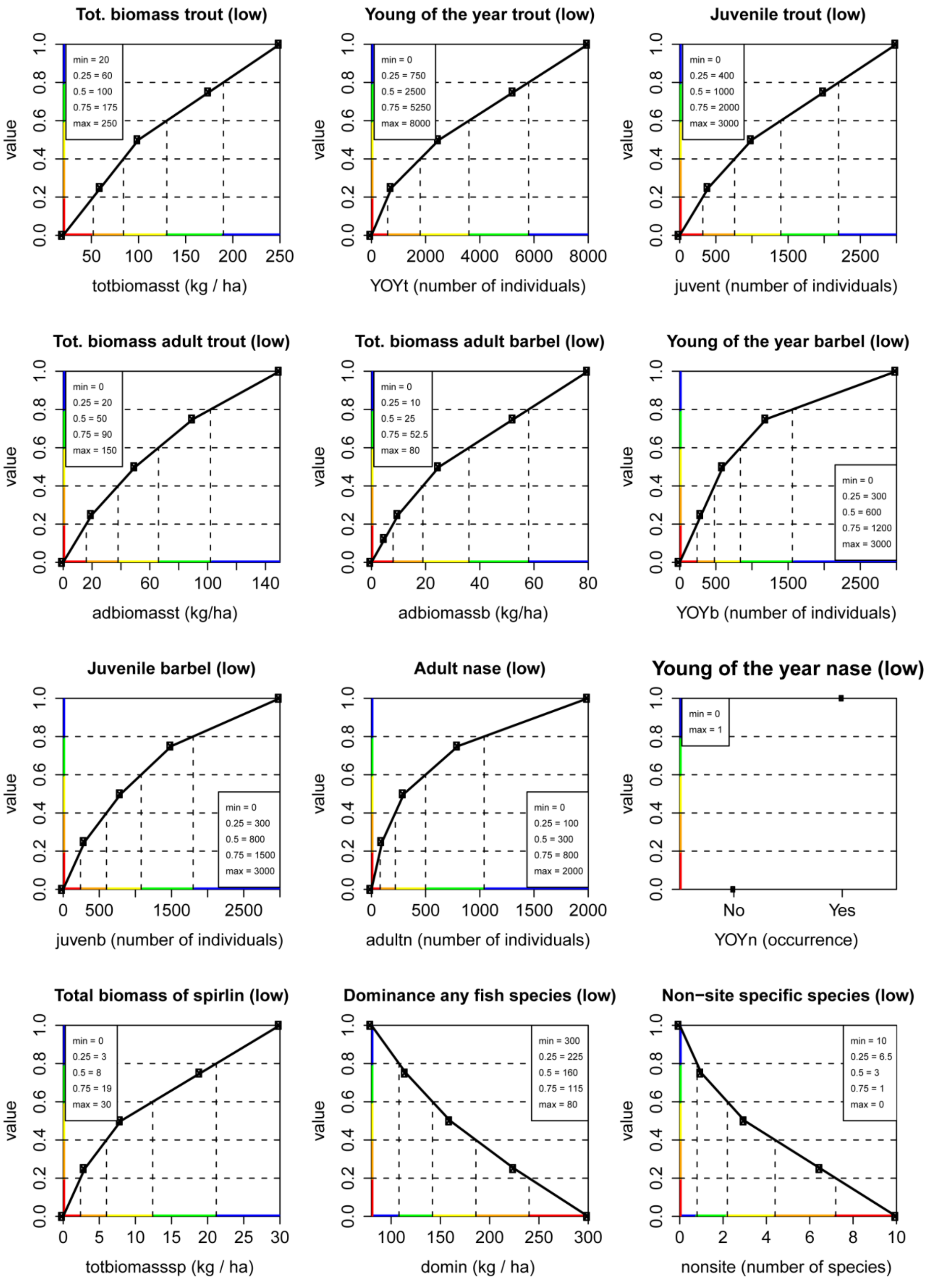

Figure K. Value functions fish. Value functions for the attributes of "natural fish diversity" for the lower reaches of a Wigger-type river, elicited from the Fish-expert. Details see Tabs. S9 and S10 and text.
