# Supplementary material for: Four Common Simplifications of Multi-Criteria Decision Analysis do not hold for River Rehabilitation
Source: PLoS One. 2016 Mar 8;11(3):e0150695. doi: 10.1371/journal.pone.0150695 (PMC4783037; doi:10.1371/journal.pone.0150695)
Supplement: S6 File — (PDF) [file pone.0150695.s006.pdf]

## Reference river: Wigger (Switzerland)

As a reference river, we used the lower (braiding) reaches of the Wigger. The Wigger is a typical smaller river of 41 km length in the Swiss Midlands (cantons Lucerne and Aargau). Its source is at 1300 m asl and it first flows through a steep and narrow valley towards the town of Hergiswil. From there on, and especially near the town of Willisau, the valley becomes much broader and opens up into a flat plain. The Wigger is channelized in large parts of its lower reaches. The Wigger is an important influent stream of the Aare (395 m asl; between Rothrist and Aarburg) with a North-South flow direction throughout. The catchment has a surface area of 368 $km^2$. The flood risk probability (peak water level for year; 1980 – 2010) is: 74 $m^3$ /s discharge (2 yr return period), 100 $m^3$ /s (5 yr), 119 $m^3$ /s (10 yr), 165 $m^3$ /s (50 yr), and 186 $m^3$ /s (100 yr return period). For more details please consult the hydrological data website of the Swiss Federal Office for the Environment (http://www.hydrodaten.admin.ch/en/2450.html).
