# Supplementary material for: Four Common Simplifications of Multi-Criteria Decision Analysis do not hold for River Rehabilitation
Source: PLoS One. 2016 Mar 8;11(3):e0150695. doi: 10.1371/journal.pone.0150695 (PMC4783037; doi:10.1371/journal.pone.0150695)
Supplement: S7 File — (PDF) [file pone.0150695.s007.pdf]

Example of an interview to elicit preferences

Below we provide an example of typical questions of the decision analyst (DA) in an interview to ask for an objective/attribute, a value function, an aggregation method, weights, and the risk attitude. E: Possible answers by the expert.

Elicitation of lower-level objectives/attributes for "ecosystem function"

Decision analyst (DA): *Let's look at "ecosystem function". What functions characterize this higher-level objective? Expert (E): ...*

DA: *In a previous project, two objectives were used to quantify "ecosystem function: "population/ ecosystem resilience" and "functioning organic cycles". Are these two adequate?"* E: ...

DA: *Which attributes would you use to measure "population/ecosystem resilience"? What are the units, which measurement method is appropriate, and what are possible ranges from worst to best?* E: ...

DA: *How would you classify the attribute: absolutely essential which means that it would be very difficult to assess "ecosystem function" without this attribute, very valuable, or desirable? The latter means that the assessment can be carried out without this attribute.* E: ...

DA compiles and harmonizes all interview data.

DA: *We have two objectives that characterize "ecosystem function": "ecosystem resilience" and "functioning organic cycles". Do you need both to quantify the state of "ecosystem function"?* E: ...

Elicitation of value function for the attribute "density of thermal refugia"

DA: *What is the worst- and best-possible state that you can imagine for this attribute? Is there an optimum, if yes, where?* E: *The worst state would be 0%, and the best state 50% of the instream area featuring significantly lower temperature than the surroundings.*

DA: *Let's picture a river reach, which is in a bad state. Your attribute is at the minimum possible, i.e. 0%. You have not achieved your goal of good "ecosystem stability" at all. Let's imagine that you could rehabilitate this river in such a way that it reaches an optimal state. Your attribute would reach its maximum, i.e. you have thus achieved your goal which is the good state in "density of thermal refugia" (i.e. 50%). Now imagine that you could improve the currently bad state in such a way that half of your goal is achieved. What would your attribute have to be so that you agree that the improvement of the worst state which is 0% to the new number is exactly as good as the improvement of this number to the maximum which is 50%?* E: *This would be 15%.*

DA: *This means that improving from 0 to 15% density of thermal refugia is exactly as good as improving from 15% to 50%. Is this correct?* E: *Yes.*

DA continues as above for $x_{0.25}$ and $x_{0.75}$. E chooses $x_{0.25}$ to be 8% and $x_{0.75}$ to be 23%.

DA: *Now I check whether you agree with the results: Imagine an improvement from the point where one quarter of your goal is achieved, which you have chosen to be 8% of the instream area featuring significant lower temperature than the surroundings. The improvement from 8% to "xy" is as good as an improvement from "xy" to the point where 75% of your goal is achieved (=23%). How high is "xy"? (This should be again be half of the goal achieved, i.e. 15%).* E: ...

Elicitation of aggregation method for "ecosystem stability"

DA: *We would like to know, how you aggregate the six essential objectives (Fig 2) which you have selected to characterize the higher-level objective "ecosystem stability". Let´s first check whether you favor an* ***"additive aggregation"****. Imagine that "density of thermal refugia" is in a bad state. To improve thermal refugia, would you accept that another objective is worsened, e.g. the "proportion of shoreline length to channel length"?* E: ...

DA: *So, do you agree or disagree that a bad performance of "density of thermal refugia" can be compensated with a good performance of "proportion of shoreline length to channel length"?* E: ...

DA: *Formulated differently: Imagine that in one rehabilitation project (A), some objectives are reached well but others poorly. In another project (B), all objectives reach a mid-value, there are no extremes. If you compare these two projects, both achieve about an equally good (medium) value in the total valuation. Does this correspond to your judgment?* E: ...

DA: *Another assumption for the additive model is so-called "preferential independence". This means that you can formulate your preferences, e.g. about "ecosystem stability" without knowing the state of the other objectives. So you can give a judgment without knowing whether e.g. "biodiversity" is in a good or bad state. Is this independence condition okay for you?* E: ...

DA: *Let's consider the implication of a potential* ***"minimum aggregation"****. If "density of thermal refugia" is in a poor state, this valuation is passed on to the next-higher level of the objectives hierarchy. This implies that the objective "ecosystem stability" receives an equally poor valuation, even if one or several of the five other objectives are in a good state. Does this correspond to your judgment?* E: ...

DA: *Let's look at* ***"multiplicative aggregation"*** *using the above example: Imagine that in rehabilitation project (A), some objectives are reached well, but others are reached poorly. In another project (B), all objectives reach approximately a mid-value, there are no extremes. If you use multiplicative aggregation, you "penalize" the extreme values in object (A) so that the total value of "ecosystem stability" is lower than in project (B), where both attributes are on a similar medium level (see Figure A in S4 File). Does this correspond better or worse to your judgment than the additive case?* E: ...

DA (if better): *This means in other words that the multiplicative aggregation entails a preference for having for example all six objectives in a relatively good state. You do not want to have one missing or in a bad state, even if the other ones are in a good state. The valuation is not independent for each objective; rather there is an added value of an integral consideration of all of them together which is called synergy effect. Would you want to give a large (sum of weights =0.25), medium (0.5), or a small (0.75) synergy effect? Please look at this diagram (see Figure B in S4 File), so that we can discuss the implications of the synergy effect.* E: ...

Elicitation of weights for the objectives of "ecosystem stability"

DA: *Imagine all six objectives are in their worst state. They receive a value of 0. Please choose one objective which you put into its best state which means you assign a value of 1. All other objectives remain in their worst state. This implies that you find it most important to improve this objective into its best state. Which one would you choose?* E: ...

DA: *You can now choose the second most important objective and put it into its best state (value =1). All other objectives are again in their worst state. Which one would you now choose?* E: ...

DA repeats the questions for the remaining four objectives of this node.

DA: *Your preferred outcome receives 100 points, which means that your most important objective is in its best state and all other objectives are in their worst state. The outcome where all objectives are on their worst level receives 0 points. How many points would you give to the other outcomes so that it correctly reflects how much better or worse they are compared to the other outcomes?* E: ...

DA: *We found that the "density of thermal refugia" being on its best level and all other objectives on their worst receives 100 points, and the "proportion of channel length/ shoreline length" 50 points. This means that having thermal refugia in the best state is twice as important to you as having "proportion of channel length/ shoreline length" in its best state and all others in their worst. Is this correct?* E: ...

DA: Continues with the same questions for the remaining four objectives.

Elicitation of risk attitude

DA: *Imagine that after a rehabilitation measure (A) there is a 50% chance that your river is in its best state, which means it gets a value of 1, but also a probability of 50% that it is in its worst state, which means it gets a value of 0. Hence, the outcome of measure (A) is highly uncertain. You could choose between this uncertain measure (A) and a certain measure (B). This certain measure (B) improves the river to a medium state, which means it gets a value of 0.5 with a 100% chance. Would you prefer measure (A) or (B)?* E: ...

DA repeats similar closed questions until decision maker is indifferent between lottery and certain situation. Hereby, the certainty equivalent (CE) for $x_{0.5}$ is elicited (see Fig. 4).

DA: *Generally asking: if you compare the lottery with a 50% probability that your river is in its best state and a 50% probability that it is in its worst state after measure (A) is implemented with a certain situation expected after the implementation of measure B: how high would the value of the certain situation have to be so that you would be indifferent between the certain and the uncertain situation?* E: ...

DA repeats similar questions to elicit the certainty equivalent (CE) for $x_{0.25}$ (and in the same manner for $x_{0.75}$, or any other points of the utility function; see Fig. 4):

DA: *Imagine that you have an uncertain situation, where there is a 50% probability that your river is in its medium state, which means the value is 0.5 – but you also have a probability of 50% that it is in its worst state, which means the value is 0. You could choose between this uncertain situation and a certain situation, where you have a 100% probability that your ecosystem is in a relatively bad state; which means the value is 0.25. What would you choose?* E: ...

DA repeats similar closed questions until decision maker is indifferent between lottery and certain situation.

DA: *So, an uncertain situation with a 50% probability that the river is in a bad state with a value of 0.25 and a 50% probability that it is in a good state with a value of 0.75 is just as good to you as if the river is in a nearly-medium state with a value of 0.4. Do you agree?* E: ...

DA: *Would you give the same answer if we consider a lower-level objective of the hierarchy, e.g. biodiversity?* E: ....
