# Supplementary material for: Four Common Simplifications of Multi-Criteria Decision Analysis do not hold for River Rehabilitation
Source: PLoS One. 2016 Mar 8;11(3):e0150695. doi: 10.1371/journal.pone.0150695 (PMC4783037; doi:10.1371/journal.pone.0150695)
Supplement: S1 Table — (PDF) [file pone.0150695.s008.pdf]

S1 Table. Selection of expert value functions for the generalized model. For nine attributes we elicited more than one value function from different experts; here we show which ones we recommend to choose for follow-up analyses (for abbreviations see Tab 3; main article).

Attribute	Abbreviation	Belonging to objective (level 3)	Selected expert
Amplitude of artificial flow variation	flowampl	No hydropeaking	BioPhys
Frequency of bed-moving flood	floodbed	Natural flood dynamics	Phys
Frequency of floodplain flooding	floodplain	Natural flood dynamics	BioPhys
Density of thermal refugia	refug	Ecosystem stability	BioPhys
Proportion shoreline length/ channel length	shorelength	Ecosystem stability	BioPhys
Proportion of natural tributaries / river length	tributar	Ecosystem stability	BioPhys
Proportion of softwood vegetation / river length	softw	Natural floodplain vegetation	BioPhys
Proportion of gravel bars / length	gravel	Natural floodplain	BioPhys
Relative proportion of periphyton	periph	Benthos natural feeding groups	BioA
