# Supplementary material for: Four Common Simplifications of Multi-Criteria Decision Analysis do not hold for River Rehabilitation
Source: PLoS One. 2016 Mar 8;11(3):e0150695. doi: 10.1371/journal.pone.0150695 (PMC4783037; doi:10.1371/journal.pone.0150695)
Supplement: S2 Table — Identified attribute levels for the worst-possible state (value = 0), for the values 0.1, 0.25, 0.5, 0.75, and 0.9, and the best-possible state (value = 1) are shown. (PDF) [file pone.0150695.s009.pdf]

S2 Table. Value functions elicited from six river experts. For each attribute (attr) we also give the higher-level objectives (level 1 = highest; compare Fig. 1; main text). The abbreviations are used in the following analyses. We give the attribute levels for the worst-possible state (value = 0), the levels for values of 0.1, 0.25, 0.5, 0.75, and 0.9, and the best-possible state (value = 1). Experts from whom we elicited these values, see Tab 2 (main text). Comments concerning the attribute and the shape of value functions are given in S3 Table.

Level	Objective / attribute	Abbreviation	Attribute description, units	v = 0	0.10	0.25	0.50	0.75	0.90	v = 1	Expert	
1	Good quality of hydromorphology	phys										
	Morphological river type: braided	braided										[illegible]
2	Natural river morphology and hydraulics	morph										
3	Natural deposition/ erosion	erosion										
attr	Sediment transport	sedtrans	m^3 sediment/ year	0	200	500	725	1'200	2'280	3'000	Phys	[illegible]
3	High diversity of substrate of river bed	substr										
attr	Diversity of patches of river bed (coarse substrate)	patchdiv-L	diversity of patches in LAWA quality classes (none, few, some, large, very large diversity of substrates)	7		(0.333 =class 5)	4	(0.833 =class 2)		1	Literature	[illegible]
3	Natural channel geometry	geom										
attr	Total sinuosity (braiding rivers)	sinuos	length of braids/ river length (m/ m)	1	1.0	1.1	1.2	1.9	2.6	3	Phys	
3	Natural flow diversity	flow										
attr	Relation between depth and velocity distribution	depthveloc	Shannon Weaver diversity index of Froude numbers	0	0.1	0.25	0.5	0.75	0.9	1	BioPhys	[illegible]
2	Natural discharge regime	disch										
3	No hydropeaking	hydrpeak										
attr	Amplitude of artificial flow variation	flowampl-F	maximal discharge (l/ s)	20'000 – 28'000	18'600	16'500	13'000	9'500	7'400	4'000 – 6'000	Fish	
attr	Amplitude of artificial flow variation	flowampl-BP	ratio between high and low discharge/ day [(m^3/ s) / (m^3/ s)]	8	7	6.3	4.5	2.8	1.1	0	BioPhys	
attr	Rate of decrease of artificial flow variation	flowrate	decrease of water level (cm/ h)	40 – 200	38	33.8	27.5	21.3	14.5	10	Fish	
3	No water abstraction	abstr										
attr	Maximal discharge	dischav	% deviation of maximal discharge from reference river	100	90	75	50	25	10	0	BioPhys	
attr	5th percentile of discharge distribution	dischdist	% deviation of 5th percentile of discharge distribution from reference river	100	90	75	50	25	10	0	BioPhys	

Level	Objective / attribute	Abbreviation	Attribute description, units	v = 0	0.10	0.25	0.50	0.75	0.90	v = 1	Expert	
3	Natural flood dynamics	flood										
attr	Discharge of annual flood	flooddisch	% deviation of discharge of annual flood from reference river	100	90	75	50	25	10	0	BioPhys	
attr	Frequency of bed-moving flood	floodbed-BP	% deviation of frequency of bed-moving flood from reference river	100	90	75	50	25	10	0	BioPhys	
attr	Frequency of bed-moving flood	floodbed-P	HQ_{xx}: years between riverbed-forming discharges								Phys	
	Frequency of bed-moving flood: increase					1	1.9	2.4	2.7	3		[illegible]
	Frequency of bed-moving flood: decrease			20	18.6	16.5	14	9.4	6.8	5		[illegible]
attr	Frequency of floodplain flooding	floodplain-BP	% deviation of frequency of floodplain flooding from reference river	100	90	75	50	25	10	0	BioPhys	
attr	Frequency of floodplain flooding	floodplain-L	number of floodings/ year	0.00	0.12	0.18	0.19	0.38	0.65	1	Literature	[illegible]
2	Good connectivity	conn										
3	High longitudinal connectivity	longcon										
attr	Height of artificial barriers (barriers for mix of fish species)	barrheight	height of barrier (cm)	50 – 100	46	40	30	20	14	0 – 10	Fish	
attr	No power stations	nopowerstat	number of power stations à 1 KW	4 – 6	3.2	2	1	0.5	0.2	0	Fish	
3	High lateral connectivity	latcon										
attr	Fraction of natural river banks	ripbank	length of both banks that are natural/ total length of both banks (m/ m)	0	0.1	0.25	0.5	0.75	0.9	1	BioPhys	
attr	Relative shoreline length only straight and braided river	shorelength	length of river (thalweg)/ total length of the river marigins of the two banks (m/ m)	2	3.0	4.6	7.7	11.9	17.6	28	BioPhys	
attr	Total width between levees and river	leveeswidth	distance between levees/ total "natural" floodplain (m/ m)	0.05	0.07	0.11	0.18	0.35	0.53	1	BioPhys	
attr	Incision	incision	incision depth (m)	5	3	1.6	0.7	0.25	0.1	0	BioPhys	
3	High vertical connectivity	vertcon										
attr	Substrate clogging	substrclog-L	class for clogging	5		4	3	2		1	Literature	[illegible]
attr	Substrate armoring (äussere Kolmation)	substrarmor	relative values of sigma = sqrt(D16/ D84)	0	0.12	0.30	0.48	0.57	0.63	1	Phys	
attr	Vertical hydrological exchange	hydrex	exchange in the river / exchange in a reference river	0.0001	0.03	0.07	0.16	0.30	0.50	1	BioPhys	
1	High level of chemical integrity	chem										
2	High physico-chemical quality	physicochem										
3	Natural temperature regime	temp										
attr	Maximal temperature in summer	tempsummax	maximum temperature in summer (° C)	20 – 24	19.7	19.3	18.5	17.8	17.3	10 – 17	Fish	

Level	Objective / attribute	Abbreviation	Attribute description, units	v = 0	0.10	0.25	0.50	0.75	0.90	v = 1	Expert	
attr	Seasonally averaged temperature	tempav	maximum deviation of average temperat. compared to reference river (° C)	15	13.5	11.3	7.5	3.8	1.5	0	BioPhys	
attr	Absolute maximal temperature small river	tempmax	maximal temperature of river (over years) compared to reference river (° C)	15	13.5	11.3	7.5	3.8	1.5	0	BioPhys	
attr	Spatial temperature distribution small river	tempdistr	maximal temperature difference between different plots in river during warmest part of day (° C)	0	0.6	1.4	2.1	2.6	3.2	5	BioPhys	
attr	Daily amplitude small river	amplday	deviation of daily amplitude of temp. of river (° C) from reference river (° C)	20	18	15	10	5	2	0	BioPhys	
attr	Gradient / slope of heating	heatslope	gradient of heating of river (° C)/ hr – grad. of heating of reference river (°C)/ hr	2	1.8	1.5	1	0.5	0.2	0	BioPhys	
attr	Gradient / slope of cooling	coolslope	gradient of cooling of river (° C)/ hr – grad. of cooling of reference river (°C)/ hr	2	1.8	1.5	1	0.5	0.2	0	BioPhys	
3	Natural level of suspended solids	sussolid										
	Total suspended solids	sussolidtot-L	total suspended solids (mg/ l)	500	350	178.75	72.5	18.75	7.5	0	Literature	i
	Mean suspended solids concentration at low discharge	sussolidlow	suspended solids concentration (grams/ m³)								No value function	
	Deposition of solids in floodplain	sussoliddepos	occurrence (yes/ no)	0						1	BioC	j
2	Good water quality	watqual										
1	High level of biological integrity	bio										
2	Natural ecosystem function	ecosys										
3	Functioning organic cycles	orgcycl										
4	Organic cycles spring	orgcyclspr										
attr	Mean ecosystem respiration, R (spring)	respirspr	g O_2/ (m^2d)								BioPhys	f
	Respiration spring: increase			0	0.9	2.2	3.2	4	5.8	7		
	Respiration spring: decrease			14	13.1	11.8	10.8	10	8.2	7		

Level	Objective / attribute	Abbreviation	Attribute description, units	v = 0	0.10	0.25	0.50	0.75	0.90	v = 1	Expert	
attr	Mean primary production, P (spring)	prodspr	g O_2/ (m^2d)								BioPhys	[illegible]
	Production spring: increase			0	0.2	0.4	0.8	1.4	2.1	2.5		
	Production spring: decrease			10	8.2	5.5	4.5	3.6	2.9	2.5		
4	Organic cycles summer	orgcyclsu										
attr	Mean ecosystem respiration, R (summer)	respirsu	g O_2/ (m^2d)								BioPhys	[illegible]
	Respiration summer: increase			0	0.8	2	2.5	3	4.2	5		
	Respiration summer: decrease			10	9.2	8	7.5	7	5.8	5		
attr	Mean primary production, P (summer / fall)	prodsu	g O_2/ (m^2d)								BioPhys	[illegible]
	Production summer: increase			0	0	0.1	0.2	0.3	0.4	0.5		
	Production summer: decrease			10	7	2.4	1	0.8	0.6	0.5		
4	Organic cycles fall	orgcyclfa										
attr	Mean ecosystem respiration, R (fall)	respirfa	g O_2/ (m^2d)								BioPhys	[illegible]
	Respiration fall: increase			0	1.7	4.3	6	7	8.8	10		
	Respiration fall: decrease			20	18.3	15.7	14	13	11.2	10		
attr	Mean primary production, P (summer / fall)	prodfa	g O_2/ (m^2d)								BioPhys	[illegible]
	Production fall: increase			0	0	0.1	0.2	0.3	0.4	0.5		
	Production fall: decrease			10	6.9	2.3	1	0.8	0.6	0.5		
3	Ecosystem stability	ecostabil										
attr	Density of thermal refugia	refug-BP	% area (m^2) with significant drop of temperature (max temp – temp in certain spot in ° C)	0	0.1	1	4.6	10	28	40	BioPhys	
attr	Density of thermal refugia	refug-BB	% area (m^2) w. sign. drop of temperature (max temp – temp in certain spot in ° C)	0	3.2	8	15	23	39.2	50	BioB	
attr	Proportion shoreline length/ channel length	shorelength-BP	shoreline length/ channel length (m/ m)	2	7.8	16.5	31	45.5	54.2	60	BioPhys	
attr	Proportion shoreline length/ channel length	shorelength-BB	shoreline length/ channel length (km/ km)	2	5.8	8.2	10.2	12.3	14.6	17	BioB	[illegible]
attr	Proportion shoreline length/ channel length	shorelength-BC	shoreline length/ channel length (m/ m)	2	2.2	2.6	2.9	3.3	3.7	4	BioC	
attr	Proportion natural tributaries per river length	tributar-BP	% of tributaries in a natural state/ all theoretically possibly natural tributaries	0	10	25	50	75	90	100	BioPhys	

Level	Objective / attribute	Abbreviation	Attribute description, units	v = 0	0.10	0.25	0.50	0.75	0.90	v = 1	Expert	
attr	Proportion natural tributaries per river length	tributar-BB	% of tributaries in a natural state/ all theoretically possibly natural tributaries	0	6.3	15.6	31.3	46.9	75	100	BioB	
attr	Proportion natural tributaries per river length	tributar-BC	% of tributaries in a natural state/ all theoretically possibly natural tributaries	0	18	35	50	65	80	100	BioC	
attr	Refugia: structural diversity	structdiv	% of deadwood area per total area of river section								BioB	[illegible]
	Structural diversity increase			0	0.6	1.4	3.5	7	11.8	15		
	Structural diversity decrease			100	97	92.5	85	77.5	73	70		
attr	Dispersal: drift / benthos ratio	driftbenthos	% of benthos that is in drift (of all benthos) at medium discharge of river								BioC	[illegible]
	Dispersal drift/ benthos: increase			10	8.2	5.5	4	3.3	3	2		
	Dispersal drift/ benthos: decrease						0	0.2	0.5	1.5		[illegible]
attr	Substrate armoring (colmation) for vertical refugia	colm	% of the total interstitial space clogged with fine sediments	100	80	60	40	30	20	0	BioC	
2	Natural diversity	divers										
3	Natural floodplain vegetation	veget										
attr	Proportion of softwood vegetation/ length	softw-BP	[(area of softwood vegetation (m^2)/ area of wetted channel (m^2))/ 1 m river length] = [proportion/ m]	0	0.2	0.5	1	2.7	4.1	5	BioPhys	
attr	Area of softwood vegetation/ length	softw-BB	m^2 softwood forest/ m river length	0	4	10	20	28.3	35.3	40	BioB	
attr	Proportion of hardwood vegetation/ length	hardw	[(area of hardwood vegetation (m^2)/ area of wetted channel (m^2))/ 1 m river length] = [proportion/ m]	0	0.2	0.5	1	3.5	5	6	BioPhys	
attr	Proportion of pioneer vegetation/ length	pionveg	[(area of pioneer vegetation (m^2)/ area of wetted channel (m^2))/ 1 m river length] = [proportion/ m]	0	0.2	0.5	1	2.7	4.1	5	BioPhys	
attr	Proportion of gravel bars/ length	gravel-BP	[(area of gravel bars (m^2)/ area of wetted channel (m^2))/ 1 m river length] = [proportion/ m]	0	0.1	0.2	0.3	0.9	1.6	2	BioPhys	
attr	Area of gravel bars/ length	gravel-BB	% gravel bars/ m river length	0	20	30	40	55	70	100	BioB	
3	Natural benthic organisms	benthos										[illegible]

Level	Objective / attribute	Abbreviation	Attribute description, units	v = 0	0.10	0.25	0.50	0.75	0.90	v = 1	Expert	
4	Benthos natural feeding groups	feedgr										
attr	Relative proportion of scrapers	scrap	% (individuals/ m^2)								BioA	[illegible]
	Scrapers: increase			0	3	7.5	15	22.5	27	30		
	Scrapers: decrease			100	81	52.2	45	37.5	33	30		
attr	Relative proportion of shredders	shred	% (individuals/ m^2)								BioA	[illegible]
	Shredders: increase			0	2	5	10	15	18	20		
	Shredders: decrease						40	30	24	20		[illegible]
attr	Relative proportion of predators	pred	% (individuals/ m^2)	0	1	2.5	5	7.5	9	10 - 15	BioA	
attr	Relative proportion of collectors-gatherers	collgath	% (individuals/ m^2)								BioA	[illegible]
	Collectors-gatherers: increase			0	2	5	10	15	18	20		
	Collectors-gatherers: decrease			50	47	42.5	35	27.5	23	20		
attr	Relative proportion of filterers	filter	% (individuals/ m^2)								BioA	[illegible]
	Filterers: increase			0	2	5	10	15	18	20		
	Filterers: decrease			100	92	80	60	40	28	20		
attr	Relative proportion of periphyton	periph-BA	% (individuals/ m^2)								BioA	[illegible]
	Periphyton: increase			0	5	12.5	25	37.5	45	50		
	Periphyton: decrease			100	95	87.5	75	62.5	55	50		
attr	Seasonally averaged density of periphyton	periph-BB	g ash free dry biomass (AFDM)/ m^2								BioB	[illegible]
	Periphyton: increase			0	10	25	50	71	89	100		
	Periphyton: decrease			200	190	175	150	129	111	100		
4	Natural diversity of macroinvertebrates	macroinvert										
attr	Natural diversity of macroinvertebrates	shannonw	Shannon Weaver Index	0	0.4	0.9	1.8	2.9	3.6	4	BioPhys	[illegible]
attr	Natural diversity of macroinvertebrates	macrinv-BioB	% (use any index)	0	19	47	60	70	88	100	BioB	
4	Reti-index for benthos	reti-index										
attr	Reti-index: (scrapers + wood-eaters + shredders) / all feeding types	reti-index	Reti-index = (scrapers + wood-eaters + shredders) / (all feeding types) (%)	0	4	10	20	30	36	40 - 50	BioA	[illegible]
4	F-13-Yoshimura-index for benthos	F13-index										
attr	F13 Yoshimura-index: (scrapers + filterers)/ (shredders + gatherer-collectors)	F13-index	F13 Yoshimura-index = (scrapers + filterers)/ (shredders + gatherer-collect.)	0.20	0.31	0.46	0.73	0.99	1.15	1.25	BioA	[illegible]

Level	Objective / attribute	Abbreviation	Attribute description, units	v = 0	0.10	0.25	0.50	0.75	0.90	v = 1	Expert	
3	Natural shoreline fauna	fauna										
attr	Mean density of ground beetles	grbeetl	individuals/ m^2	0	0.4	1	3	6.5	7	10 – 50	BioA	
attr	Mean density of rove beetles	rovbeetl	individuals/ m^2	0	0.2	0.5	1	2.5	3	4 – 20	BioA	
3	Natural fish diversity, lower reaches Wigger	fish_low										c
4	Natural trout population (*Salmo trutta*)	trout										
attr	Total biomass of trout	totbiomasst	kg/ ha	20	36	60	100	175	220	250	Fish	
attr	Number of young of the year trout	YOYt	number of individuals	0	300	750	2'500	5'250	6'900	8'000	Fish	
attr	Number of juvenile trout	juvent	number of individuals	0	160	400	1'000	2'000	2'600	3'000	Fish	
attr	Total biomass of adult trout	adbiomasst	kg/ ha	0	8	20	50	90	126	150	Fish	
4	Natural barbel population (*Barbus barbus*)	barbus										
attr	Total biomass of adult barbel and/ or chub (*Leuciscus cephalus*)	adbiomassb	kg/ ha	0	4	10	25	52.5	69	80	Fish	
attr	Number of young of the year barbel	YOYb	number of individuals	0	120	300	600	1'200	2'280	3'000	Fish	
attr	Number of juvenile barbel and/ or chub (*Leuciscus cephalus*)	juvenb	number of individuals	0	120	300	800	1'500	2'400	3'000	Fish	
4	Natural nase population (*Chondrostoma nasus*)	nase										
attr	Number of adult nase	adultn	number of individuals	0	40	100	300	800	1'520	2'000	Fish	
attr	Occurrence of young of the year nase	YOYn	yes/ no	0						1	Fish	j
4	High biomass of spirlin	spirlin	(Albumoides bipunctatus)									
attr	Total biomass of spirlin	totbiomasssp	kg/ ha	0	1	3	8	19	25.6	30	Fish	
4	No competitors	compet										
attr	Dominance of any fish species	domin	kg/ ha	300	270	225	160	115	94	80	Fish	
attr	Number of non-site-specific species	nonsite	number of species	10	9	6.5	3	1	0.4	0	Fish	
4	No fish with anomalies or injuries	anom										
attr	Percent fish with anomalies or injuries	anom	% fish w. anomalies or injuries (of all fish)	50	34	10	5	2.5	1	0	Fish	
3	Natural fish diversity, mid reaches Wigger	fish_mid										r
4	Natural trout population (*Salmo trutta*)	troutm										
attr	Total biomass of trout	totbiomasstm	kg/ ha	20	32	50	100	170	248	300	Fish	
attr	Number of young of the year trout	YOYtm	number of individuals	0	200	500	2'000	6'000	8'400	10'000	Fish	
attr	Number of juvenile trout	juventm	number of individuals	0	140	350	800	2'000	3'200	4'000	Fish	
attr	Total biomass of adult trout	adbiomasstm	kg / ha	0	12	30	75	125	170	200	Fish	
4	Natural barbel population (*Barbus barbus*)	barbusm										
attr	Total biomass of barbel	totbiomassbm	kg/ ha	0	3	7.5	15	25	40	50	Fish	
attr	Number of young of the year barbel	YOYbm	number of individuals	0	80	200	400	800	1'520	2'000	Fish	

Level	Objective / attribute	Abbreviation	Attribute description, units	v = 0	0.10	0.25	0.50	0.75	0.90	v = 1	Expert	
attr	Number of juvenile barbel	juvenbm	number of individuals	0	48	120	360	680	872	1'000	Fish	
4	Optimal stone loach population	loachm	(Barbatula barbatula)									
attr	Biomass of stone loach	totbiomasslm	kg/ ha								Fish	[illegible]
	Stone loach: increase			0	1	1.5	3	6.5	9	10		
	Stone loach: decrease			60	60	60	26	19	14	10		
4	No competitors	competm										
attr	Dominance of any fish species	dominm	kg/ ha	200	178	145	90	70	58	50	Fish	
attr	Number of non-site-specific species	nonsitem	number of species	10	9	6.5	3	1	0.4	0	Fish	
4	No fish with anomalies or injuries	anomm										
attr	Percent fish with anomalies or injuries	anomm	% fish w. anomalies or injuries (of all fish)	50	34	10	5	2.5	1	0	Fish	
3	Natural fish diversity, head water Wigger	fish_head										[illegible]
4	Natural trout population (Salmo trutta)	trouth										
attr	Total biomass of trout	totbiomassth	kg/ ha	20	32	50	100	170	248	300	Fish	
attr	Number of young of the year trout	YOYth	number of individuals	0	200	500	2'000	6'000	8'400	10'000	Fish	
attr	Number of juvenile trout	juventh	number of individuals	0	140	350	800	2'000	3'200	4'000	Fish	
attr	Total biomass of adult trout	adbiomassth	kg/ ha	0	12	30	75	125	170	200	Fish	
4	No fish with anomalies or injuries	anomh										
attr	Percent fish with anomalies or injuries	anomh	% fish w. anomalies or injuries (of all fish)	50	34	10	5	2.5	1	0	Fish	

Footnotes to Table S9

a) Choose natural river type first: either straight/ alternate, or braiding, or meandering (slightly different objectives).

b) Value function was elicited for river Sihl (Einsiedeln – Zürich), not for Wigger.

c) Classes from the literature: LAWA (2000) "translated" into discrete values according to Langhans et al. (2013).

d) Shannon Weaver Index: Shannon and Weaver (1963).

e) Minimum at increasing branch is 1 and corresponds to a value of v = 0.33.

f) Value function with an optimum, i.e., an increasing and decreasing branch.

g) Classes from LAWA (2004) "translated" into continuous value function according to Langhans et al. (2013).

h) Classes from Schälchli (2002) "translated" into discrete values according to Langhans et al. (2013).

i) Values from Strager et al. (2000), US EPA (2003), and own assumptions “translated” to a continuous value function (Langhans and Reichert, 2011; Langhans et al., 2013)

j) Yes (value = 1) or No (value = 0) attribute.

k) Maximal value based on literature: van der Nat et al. (2002).

l) The minimum at the decreasing branch equals zero and has a value of v = 0.625.

m) Choose either "natural feeding groups", or an index, or "macroinvertebrates".

n) The minimum at the descending branch equals 40 and has a value of v = 0.5.

o) expbioA suggests to use indices 1 or 2 of the „Reti-Ernährungssystem“; “Rhithron-Ernährungstypen-Index”; in German (reti-feeding-system; rhitron-feeding-types-index), from Schweder (1992):

$$Reti - index = \frac{(\sum scrapers + \sum wood - eaters + \sum shredders)}{\sum all\ feeding\ types}$$

p) Yoshimura et al. (2006)

$$F13 - index = \frac{(proportion\ scrapers + proportion\ filterers)}{(proportion\ shredders + proportion\ gatherer - collectors)}$$

q) Different attributes and value functions for headwaters, mid-reaches, and lower reaches Wigger; "l" for low. We only analyzed the lower reaches since these are most likely strongly impaired and thus affected by rehabilitation measures, but give all value functions here.

r) We did not use fish-attributes in the mid-reaches for this analysis, but give the elicited value functions here, "m" for mid.

s) We did not use fish-attributes in the head waters for this analysis, but give the elicited value functions here, "h" for head.

References

Langhans, S.D., Reichert, P., 2011. Einbettung von Verfahren zur Fliessgewässerbewertung in ein übergeordnetes Gewässermanagementkonzept. Vorschläge am Beispiel des Modulstufenkonzepts. Wasser Energie Luft 103, 139-148.

Langhans, S.D., Lienert, J., Schuwirth, N., Reichert, P., 2013. How to make river assessments comparable: A demonstration for hydromorphology. Ecological Indicators 32, 264-275.

LAWA, 2000. Gewässerstrukturgütekartierung in der Bundesrepublik Deutschland – Verfahren für kleine und mittelgroße Fließgewässer, Empfehlung. Länderarbeitsgemeinschaft Wasser. http://www.lawa.de/, accessed 22.12.2014

LAWA, 2004. "Gewässerstrukturkartierung in der Bundesrepublik Deutschland - Übersichtsverfahren“; in German (Mapping the quality of water body structures in Germany - Overview procedures), Schwerin, Germany. ISBN 987-3-88961-249-6, http://www.lawa.de/, order number 300823, accessed 22.12.2014.

Schälchli, U., 2002. Innere Kolmation - Methoden zur Erkennung und Bewertung; in German (Inner substrate clogging - Methods for detection and assessment), Fischnetz-Publication (Project 01/11), Eawag, Dübendorf, Switzerland. http://www.fischnetz.ch/content_d/publ/tp.htm, accessed 22.12.2014.

Strager, M.P., Fletcher, J.J., Yuill, C.B., Strager, J.M., 2000. Not in My Watershed! An Interactive Tool to Evaluate Land Use Changes on Stream Water Quality, Prodeedings of the ESRI International User Conference. http://proceedings.esri.com/library/userconf/proc00/professional/papers/PAP663/p663.htm, accessed 22.12.2014.

USEPA, 2003. Developing water quality criteria for suspended and bedded sediments (SABS), Potential approaches, US EPA Office of Water, Office of Science and Technology, Draft, August 2003.

http://water.epa.gov/scitech/swguidance/standards/criteria/aqlife/pollutants/sediment/upload/2004_08_17_criteria_sediment_sab-discussion-paper.pdf, accessed 22.12.2014.

Weaver, W., Shannon, E., 1963. The Mathematical Theory of Communication. University of Illinois Press.

Yoshimura, C., Tockner, K., Omura, T., Moog, O., 2006. Species diversity and functional assessment of macroinvertebrate communities in Austrian rivers. Limnology 7, 63-74.
