# Supplementary material for: Four Common Simplifications of Multi-Criteria Decision Analysis do not hold for River Rehabilitation
Source: PLoS One. 2016 Mar 8;11(3):e0150695. doi: 10.1371/journal.pone.0150695 (PMC4783037; doi:10.1371/journal.pone.0150695)
Supplement: S3 Table — Attribute name, abbreviation, and details concerning the attribute or shape of the value function. (PDF) [file pone.0150695.s010.pdf]

S3 Table. Comments value functions. For each value function that was elicited from the river experts, we give the attribute name and abbreviation (used in the later analyses), and details concerning the attribute or shape of the value function. Also see S2 Table.

Attribute	Abbreviation	Comments
Sediment transport	sedtrans	Sediment transport is easier to measure in meandering than in braided rivers. It is measured with topographical surveys of the river profile, and is an important monitoring measure in practice. It is usually measured all five years. The volume balance is calculated from the river profile and distributed in the measurement period (i.e. five yrs.). The measures are not exact; one can easily have deviations up to ± 100%. A problem is the definition of the natural state (reference). Values depend on the river; our expert was only familiar with the river Sihl and gave a value function for this river (origin near Einsiedeln and flows into lake Zürich).
Diversity of patches of river bed (coarse substrate)	patchdiv-L	Phys was not able to give the shape of the value function; he would probably use Sigma (= root of (D16/ D84), where D16 = 16% of granules are smaller than xy cm diameter, and D84 = 84% of granules are smaller than xz cm diameter). One has a composition of the substrate from 0 to 20 cm diameter and determines the relative weight of each component. This number gets larger, the wider the substrate distribution. It is therefore a function of the slope: a steep river has a much higher number than a level river. In a worst-possible case, sigma would be 1, a good natural case, sigma = 6.<br>Another very good attribute, according to Phys, is to use LAWA classes. For this, one first needs to know the river type and whether it has fine or coarse substrate (coarse substrate for the Wigger). One assigns the river to one of seven classes (class 1: very large diversity of substrate, class 2: large, class 4: moderate, class 5: low, class 7: no diversity of substrate). These classes were treated as discrete and translated to values according to Langhans et al. (2013). Details see separate section in Supplementary Data.
Total sinuosity (braided rivers)	sinuos	One has to distinguish between braided and meandering rivers. The attribute is: "sum of the length of all river arms of river section (following all braids)/ (straight) length of the river section". The value function must be steep for the lower values, because an improvement when the river is in a bad state is very valuable, while the gain of additional braids, once there are some braids, is not so large.<br>For meandering rivers one has the same shape of the curve, but the range is from 1 (= minimum) to 1.8 (= maximum) for a Wigger-type river.
Relation between depth and velocity distribution	depthveloc	One measures the depth and the velocity at several (e.g. 100) cross-sections of the river. For each cross section, the Froude number is calculated: Fr = v / sqrt (g x h), where v = velocity, g = acceleration, h = water depth. The diversity of the Froude numbers is then measured with a diversity index such as the Shannon Weaver index and compared to a reference river. The higher the diversity, the better the value; BioPhys gave relative values and postulated that this is a linear relationship for the Shannon Weaver index H', where S is the total number of species and p_i is the frequency of the ith species (the probability that any given individual belongs to the species, hence p).<br>$H' = -\sum_{i=1}^{S}(p_i \ln p_i)$
Amplitude of artificial flow variation	flowampl-F	The minimal natural discharge of the Wigger is around 4,000 l/ s (4 m^3/ s). The maximum value possible (in a Wigger-type river) is around five to seven times the natural discharge, i.e. ca. 20,000 to 28,000 l/ s. Anything larger than five times the natural discharge is not acceptable. There is a linear relationship in between. In larger rivers, the values can be quite different, and in extreme cases, it can be 20 to 30 times the natural discharge.
Amplitude of artificial	flowampl-BP	If there is a hydropower plant in the river, it creates daily amplitude of the discharge. The most common indicator in Switzerland is the ratio between the high and low discharge during one day, where factor eight is an extreme worst case, and one the natural best case. In the EU this

Attribute	Abbreviation	Comments
flow variation		indicator is called the maintenance flow regime (and not the natural flow regime) with the idea to restrict the range of small hydro-electrical plants. Amplitude of artificial flow variation = (high flow during the day in m^3/ s) / (low flow during the day in m^3 / s).
Rate of decrease of artificial flow variation	flowrate	This is an indicator of daily storage of water due to a hydropower plant and hydropeaking. It measures whether the water level falls very steeply. The increase is not important, but for the decrease, speed can be a problem. The values given are for the river Saane, which is about the worst artificial flow variation one can have in Switzerland. The minimum (worst-possible case) is around 40 cm/ h to 200 cm/ h, the best case around 10 cm/ h.
Maximal discharge	dischav	For each of the four seasons the discharge is measured; the maximal value from the four seasons is taken and compared with a reference river: % deviation of discharge from reference river = 100 – ((max. discharge of river) x 100/ (natural discharge of reference river)). 100% deviation means that there is no flow, 0% that the river is in its natural state. One could also calculate % deviation of discharge separately for each season and then average over all 4 seasons.
5th percentile of discharge distribution	dischdist	The 5th percentile of discharge distribution is the discharge which is exceeded in 95% of the days. It is an indicator for seasonal storage; i.e. if there are dams in the river which are operated on a yearly regime, where water is kept back in the summer. It is compared to a reference river: % deviation of 5th percentile of discharge distribution from reference river = 100 – ((5th percentile of discharge distribution of river) x 100/ (natural 5th percentile of discharge distribution of reference river)). 0% means that the 5th percentile discharge distribution is the same as in the (natural) reference river.
Discharge of annual flood	flooddisch	The annual flood is the highest flood which occurs on average once a year (higher floods are possible, but less frequent). One compares the discharge value of the annual flood with a reference river: % deviation of discharge of annual flood from reference river = 100 – ((discharge of annual flood of river) x 100/ (natural annual flood discharge of reference river)). 100% deviation means that there is no annual flood, 0% that the river is in its natural state. For a general case, a linear relationship is a good approximation, but for more-detailed cases, one would have to introduce two to three thresholds that specify the start of different process and model the relationship between geomorphological and ecological processes.
Frequency of bed-moving flood	floodbed-BP	Compare the average size of the substrate with the size of the substrate of the reference system: % deviation of frequency of bed-moving flood from reference river = 100 – ((frequency of bed-moving flood) x 100/ (natural frequency of bed-moving flood of reference river)). 0% deviation means that the substrate size of the river is the same as in the (natural) reference river.
Frequency of bed-moving flood	floodbed-P	The minimum is HQ_{20} = statistically a river-bed forming discharge (i.e. large flood) every 20 years. There is an optimum at around one flood every three to five years.
Frequency of floodplain flooding	floodplain-BP	Compare the frequency of floodplain floodings with those in a reference system: % deviation of frequency of floodplain flooding from reference river = 100 – ((frequency of floodplain flooding) x 100/ (natural frequency of floodplain flooding of reference river)). 0% deviation means that the floodplain flooding of the river is the same as for the (natural) reference river.
Frequency of floodplain flooding	floodplain-L	According to LAWA (2004), the frequency of floodplain inundations that are at least two times as large as the river width are assessed in seven classes (class 1: natural = flooding every one – two years, class 3: impaired = every three – five yrs., class 7: strongly reduced = floodings fewer than every five yrs.). These classes were transformed into a continuous value function according to Langhans et al. (2013). Further details see separate section in Supporting Information.

Attribute	Abbreviation	Comments
Height of artificial barriers (barriers for mix of fish species)	barrheight	This value function describes barriers for all fish species, including small fish. Fish are the most important taxa for which barriers should be assessed; other species can often pass them, e.g. insects can fly. The barriers can be any artificial obstacles (weirs, but also missing or inadequate fish ladders if there are power stations). The value function is linear, because the Fish-expert does not have any good argument for another shape.
No power stations	nopowerstat	For this value function the Fish-expert assumed that the power stations have fish passes (this is the case for newer power stations). So fishes can pass the obstacle upstream. However, when they wander down the river, they often get killed, e.g. in turbines. The mortality can be anywhere between 10 – 90%. As example, assuming a mortality rate of 10%, with three power stations, the rate would be 10% x 3 = 30%. The value function certainly has an exponential decrease, but the exact numbers are very dependent on the length of the fish, the power station, the size of the turbine etc. So the given value function is just a best guess. Note, the Wigger has a small power station without a fish pass.
Fraction of natural river banks	ripbank	
Relative shoreline length only straight and braided river	shorelength	This attribute is required for braided and straight rivers, but not for meandering rivers. The shape of the curve must be asymptotic, but the maximum can depend on the measurement method. The minimum (worst value) is two because there are two banks.
Total width between levees and river	leveeswidth	If there are levees, the functioning of the river system can be impaired compared to the river being able to flow freely over the total width of the "natural" floodplain. The relationship must be an asymptotic curve so that an improvement when the river is in a bad state is of higher value than improvements if the river is already in a good state. BioPhys was only able to give relative values between 0 and 1.
Incision	incision	The depth of incision is measured from the old stream bed, which can be seen on the margins of the river, to the bottom of today's riverbed. If there is a strong incision, the water during a flood cannot leave the channel. The curve is not linear. If incision starts, this is an indication that something is going really wrong. Once incision has started, it can go on "forever". So the value decreases strongly at the beginning. In Switzerland, in a normal river, the worst case is ca. four to five meters.
Substrate clogging	substrclog-L	According to Schälchli (2002), the colmation is assessed in five classes (no colmation: substrate very loose and coarsely granular / weak colmation: substrate loose and broadly tiered / medium colmation: substrate slightly cemented / strong colmation: substrate is clearly cemented / total colmation: substrate is strongly cemented). These classes were transformed into discrete values according to Langhans et al. (2013). For details see separate section in Supplementary Data.
Substrate armoring (äussere Kolmation)	substrarm	To describe substrate armoring one uses a relationship such as sigma = square-root of the relationship D16/ D84 (of the cover layer). The shape of the curve has to be sigmoid; the Phys-expert gave relative values of sigma. D16 means that 16% of the granulas are smaller than xy cm in diameter, and D84 that 84% are smaller than xz diameter. Near the maximum, the curve has to be very flat. The worst-possible case is 1 = everything consists of the same material.

Attribute	Abbreviation	Comments
Vertical hydrological exchange	hydrex	This is a direct measure; there are various ways to measure it: tracers, piezo-meter, mapping of the hydraulic head, or sampling the radon concentration in the sub-surface compartment. The easiest way is to sample radon, but a lab is required that can do these analyses (available at Eawag). BioPhys suggests to compare the exchange in the river with a reference river that is still "natural" and that is similar with respect to geomorphology and substrate. The worst case (minimum) cannot be zero, because there are always some hydrological exchange processes going on. The shape of the curve has to be asymptotic with a steep increase in the beginning, because some exchange, if the river is in a poor state, is very important.
Maximal temperature in summer	tempsummax	According to the Fish-expert it is sufficient to take the maximal temperature in summer as attribute; no others are required for "natural temperature regime" (apart from maybe daily amplitude if there is hydropeaking). One has to distinguish between head waters and mid-/ lower reaches, but the values are similar. The maximum the Fish-expert ever measured in the Wigger was 23° C and it is highly unlikely that it would ever become warmer than 24° C (for other rivers, such as the Thur, warmer temperatures are possible). In summer, in the mid- and lower reaches, the minimum would be around 12 – 13° C (= best-possible case). The value function was derived based on the growth-optimum for trout, which is 7 – 17° C (juveniles: max. 14° C). Hence, good values (1) are from 10 – 17° C in summer, then there is a linear decrease, and from temperatures higher than 20° C the value is 0 (worst-possible state), because trout growth is impaired.
Seasonally averaged temperature	tempav	BioPhys suggests comparing the average temperature of the river (which can be measured in one spot) with the average temperature of a reference river. As reference river, choose a river that is still braided (or meandering). Even under climate change, this best-possible system will have cooler temperatures than a strongly channelized river, or one that is used to cool the water from a nuclear power plant. Maximum deviation of temperature from reference river = (max. temperature of river)/ (average temperature of river). The worst-possible case is 15° C per season, i.e. that the average temperature of the impacted river is e.g. 25 °C, while that of the reference river is 10° C. The Fish-expert states that this attribute is not at all important; the average temperature does not give much information.
Absolute maximal temperature small river	tempmax	This is an indicator of extreme events and of heat waves. The absolute maximum temperature that was ever measured in the river is compared to a reference river. The worst case for a small river such as the Wigger is 11.25°C deviation from a reference river.
Spatial temperature distribution small river	tempdistr	The spatial temperature distribution is the maximal degree of difference in the temperature between different plots in the river. It is measured during the warmest part of the day and is an indicator of whether there are cool water refugia. The relationship is a sigmoid curve. the maximal (best) value for a small river such as the Wigger is 5° C, for a medium-sized river 7° C, and for a large river (e.g. river Thur in Switzerland) 10° C).
Daily amplitude small river	amplday	Deviation of daily amplitude = (maximal temperature of river per day - minimal temperature of river per day) – (maximal temperature of reference river per day – minimal temperature of reference river per day). There is a linear relationship. In small rivers, the normal amplitude can be around 10° C, in large rivers it is less; ca. 2 – 3 ° C (in summer). Worst case for small river (e.g. Wigger): 20° C, medium-sized river: 15° C, large river (e.g. river Thur in Switzerland): 5° C. Best case is always 0 deviation from reference.
Gradient / slope of heating	heatslope	The gradient (slope) of heating = is the increase in temperature in °C per hour. It is compared to a reference river. In a natural reference river, one would have 5° C as gradient of heating, while an impacted river can have in an extreme case 7° C. The maximal difference that BioPhys ever measured was 2° C heating per hour more than in a natural river. The temperature should be measured in different spots (i.e. also over space, not only time).

Attribute	Abbreviation	Comments
Gradient / slope of cooling	coolslope	As for the gradient (slope) of heating. The values are similar: the worst case is 2° C difference to reference river under normal conditions, i.e. excluding rain events. The temperature should be measured in different spots (i.e. also over space, not only time).
Total suspended solids	sussolidtot-L	The value function for total suspended solids was derived from the literature: Strager et al. (2000), USEPA (2003), and own assumptions. See separate section.
Mean suspended solids concentration at low discharge	sussolidlow	Suspended solids concentration is measured at low discharge because of hydropower. The suspended solids are measured with a cone-like device, into which 3 – 4 liters of water are filled. The weight of the sediment is measured. BioPhys cannot give numbers for this attribute.
Deposition of solids in floodplain	sussoliddep	
Water quality	watqual	Since we had explicitly excluded the entire branch "good water quality", but included this objective in our analyses, we assumed linearity for the value function of the corresponding "attribute" (see text).
Mean ecosystem respiration, R (spring)	respirspr (respirsu, respirfa)	Respiration is measured as g O_2/ (m^2 d). The value function is a Gaussian curve, but with different values for spring, summer, and fall. It is measured together with respiration (same comment for summer and fall).
Mean primary production, P (spring)	prodspr (prodsu, prodfa)	Production is measured as g O_2/ (m^2 d), the minimum is 0 and the maximum for a river-type river 10 g O_2/ (m^2 d); both are indicative of a bad state of the river. It is an optimum curve, with different values for spring (before leaf emergence) than for summer and fall. Production can change a lot, even every 100 m in the river. It cannot be used alone, but must be combined with respiration to evaluate how the ecosystem is changing. Usually one uses the ratio of production and respiration. The values depend on the problem in the river. As example, if you have the problem of organic pollution and install a wastewater treatment plant, you will see a decrease – but not necessarily if the river is suffering from additional impacts. If the river is shaded by trees along the shore, and maybe islands and has a longer channel length, production could be lower in a Wigger-type river than it is today. If the production/ respiration values are known before the restoration, the values after restoration can be compared to measure the success of the measure (same comment for summer and fall).
Density of thermal refugia	refug-BP	To define the thermal refugia, the system is mapped with a thermal camera (infrared camera). Refugia is a place with a significant drop in the temperature, e.g. 2 – 3° C lower than the maximal temperature. If the river is really homogenous, the river is in the worst-possible state (= 0% thermal refugia). The maximal density of thermal refugia in a natural system would be 30 to 35%; and up to 40% in extremely good conditions. The curve is asymptotic.

Attribute	Abbreviation	Comments
Density of thermal refugia	refug-BB	Thermal refugia are important, as example for fish, additionally to the structural diversity which also offers refugia. BioB gave a value function, but felt unsure about it and suggested that we use the one from BioPhys for our further analyses.
Proportion shoreline length/ channel length	shorelength-BP	The maximum depends on the scale that you are measuring. Islands and backwaters in the river can add a lot. It is also important to note that this changes with water depth. To compare the river after restoration with before, you have to compare it under the same conditions, i.e. same water depth. The minimum is 2 (for the two river margins), the maximum around 60 for a Wigger-type river. Note that BioB and BioC gave totally different values for the maximum.
Proportion shoreline length/ channel length	shorelength-BB	BioB postulates that the shape of the value function is sigmoid. After some discussion, we decided to take a literature value for the Tagliamento river as best-possible case: "Shoreline lengths ranged from 28 m ha^{-1} (2.1 km km^{-1}) to 214 m ha^{-1} (16.3 km km^{-1}) in the bar-braided reach. (...) Shoreline length in the island-braided reach showed a similar range. The minimum value of 37 m ha^{-1} (3.0 km km^{-1}) occurred at complete active floodplain inundation. The maximum of 212 m ha^{-1} (17.3 km km^{-1}) occurred at water levels of between 1.0 and 1.65 m" (van der Nat et al., 2002). Hence, BioB chose 17 km km^{-1} as maximal (best-possible) state. Note that BioPhys and BioC gave totally different values for the maximum.
Proportion shoreline length/ channel length	shorelength-BC	The worst-possible case, i.e. if the river is totally straight, is two (because there are two margins of the shores), the best-possible case for this type of river around four. BioC likes this attribute, because it is easy to assess, there are good numbers available, and it is a good indicator. The shape of the value function is sigmoid, but fairly flat. Note that BioPhys and BioB gave totally different values for the maximum.
Proportion natural tributaries per river length	tributar-BP	This is very case-specific and also depends on tributaries for which species. If tributaries are mainly for fish, they have to have a certain size (example Wigger: at least 1.5 m wide and flow during three months/ yr). BioPhys cannot give concrete numbers and postulates that it is a linear relationship.
Proportion natural tributaries per river length	tributar-BB	One has to define how many tributaries one would expect per river length to be there in a "best-possible", natural state of the river; then one has to define, how many of the existing tributaries are in a natural state. BioB postulates that the value function is linear, but steeper until the value of 0.8 is reached (50% natural tributaries) and then it flattens out.
Proportion natural tributaries per river length	tributar-BC	One classifies the tributaries according to their morphology and calculates the proportion tributaries that are in a natural state. The shape of the value function depends on the river. Because rehabilitation measures are usually more downstream, the value function was given for lower reaches. It is a sigmoid relationship, and it is OK to put the turning point into the middle, i.e. with 50% of the tributaries being in a natural state, 50% of the objective is achieved. The shape of the curve is arguable; BioC would not make it too steep.

Attribute	Abbreviation	Comments
Refugia: structural diversity	structdiv	Shading and refugia are important aspects of structural diversity, additionally to thermal refugia. This is not covered by the hydromorphological attributes such as the relationship between depth and velocity distribution. 100% structural diversity is achieved if each point in a river section is different from all other points within one m^2. Structural elements would mainly be deadwood. In the best case, around 15% of the area would be covered by deadwood. If there is more deadwood, the situation deteriorates, so the value function is an optimum curve. The worst-possible case of 100% deadwood coverage is a theoretical value, which could occasionally occur after storm events. Optimal values are achieved for up to 70% of the area being covered with deadwood.
Dispersal: drift / benthos ratio	driftbenthos	There are quite good data for this in the literature. The relationship is certainly not linear, and there is an optimum at ca. 1 – 3% benthos being in drift. Around the optimum, the curve should be fairly flat. If there are around 4 – 5% of benthos in drift, the river is already in a fairly bad state, the worst-possible state is if 10% of benthos (or more) are in drift. For the increasing branch of the curve, if no benthos are in drift, the value is around 0.65.
Substrate armoring (colmation) for vertical refugia	colm	The worst-possible case is that the entire interstitial space is clogged with fine sediments (value = 0). Although the simplest relationship would be linear, BioC thinks that it is better to place the mid-point at around 60%. If there is already a good ecological level, it is ecologically more valuable to improve this to a really good state. In contrast, if the river is in a bad state, the overall value of improving the river to a medium state is not so important. However, this is also a philosophical question: does one want to improve a heavily modified (urban) river? For BioC it is more important to improve a river towards a near-natural state. The shape is sigmoid, with the steepest slope around the middle. On a logarithmic scale it would be a linear relationship.
Proportion of softwood vegetation/ length	softw-BP	It is important to consider the dimensions of the system. A small creek will not have more than 5 – 10 m^2 of softwood per meter of river length, but a large river might have hundreds. Therefore, BioPhys proposes to use the ratio between area of softwood vegetation and area of wetted channel (per meter of river length). It is difficult to define the natural river for a Wigger-type river in Switzerland, because one does not have a natural state anymore. In the interview it was defined that "best-possible state" equals what can actually be achieved in Switzerland today with best-possible restoration measures.
Area of softwood vegetation/ length	softw-BB	The calculation for the maximum value is based on 40 m from the river edge into the land-area (at a right angle to the river) and ca. 1 km length along the river: 40 m x 1,000 m = 40,000 m^2/ km = 40 m^2/ m.
Proportion of hardwood vegetation/ length	hardw	Same comment as for softwood vegetation above.
Proportion of pioneer vegetation/ length	pionveg	Same comment as for softwood vegetation above; the numbers are the same as for softwood vegetation.

Attribute	Abbreviation	Comments
Proportion of gravel bars/ length	gravel-BP	Same comment as for softwood vegetation above.
Area of gravel bars/ length	gravel-BB	Because BioB does not know the numbers, the value function was elicited as %; it was agreed to use the value function of BioPhys in the case that one value function should be chosen for further analyses.
Natural benthic organisms	benthos	To assess the diversity of benthos, the proportion of the different feeding groups can be assessed individually, or an index can be used. Our expert for benthos, BioA, prefers to use the individual feeding groups. He demonstrated why he prefers individual feeding groups using the F13-index from Yoshimura et al. (2006). In this index, the relative proportion of scrapers plus filterers is divided by the relative proportion of shredders plus gatherer-collectors. In an optimal, near-natural river, the index is calculated as: (30% shredders + 20% filterers)/ (20% shredders + 20% collectors-gatherers) = 1.25. In one type of worst-case river, the index is: (5% scrapers + 10% filterers)/ (40% shredders + 30% gatherer-collectors) = 0.214. Another bad example with no riparian vegetation or woods, and lots of fine sediments is: (5% scrapers + 10% filterers)/ (0% shredders + 70% gatherer-collectors) = 0.214. As the calculations demonstrate, to get a low value of the F13-index, different causes are possible, which are masked by the index. Therefore, BioA prefers an individual assessment of the different feeding groups (which is required anyway to be able to calculate the index). This increases transparency and helps to evaluate the causes of a problem.
Relative proportion of scrapers	scrap	Usually one uses abundance (individuals/ m^2), but it can be substituted by classes (see: http://fliessgewaesserbewertung.de/download/handbuch/). It is an optimum curve (optimum with around 30% scrapers of total benthos), with a "kink" in the descending branch at around 50% of scrapers, which equals a rather bad state of the river. For very small rivers (smaller than the Wigger), the optimum is around 10%, and for very large rivers around 2 – 3% scrapers.
Relative proportion of shredders	shred	See comment to scrapers; optimum at around 20% shredders. Shredders are deadwood, leaf etc. devourers. In water bodies of 4th order, lots of leaves enter the water bodies. If there are less than 20% shredders (of all benthos), this is not very good, it is an indication that the riparian vegetation and woods are missing. If there are more, this is indicative of shading due to riparian vegetation and woods. It is also an indicator of straightening of the river and lots of leaves entering the system. The maximal value can never be 100% because half of the benthos will be the next-following member in the succession chain, which are filterers and collectors-gatherers. If the maximum of around 40% shredders is reached, the river is not really in a bad state, so it receives a value of 0.5.
Relative proportion of predators	pred	See comment to scrapers. Predators are an interesting case. The other benthos change along the length of the river, but predators always have a maximum of ca. 10 – 15%; more is not possible. The reason is that from one trophic level to the next there is a reduction of 90%. If there are less than 10 – 15% predators, this means that there are fewer long-lived organisms, which is indicative of instable conditions. This can be caused by the load of fine sediments, by pollution, or by hydropeaking due to power stations. Theoretically, there can be zero predators, for instance if the oxygen conditions are really bad.

Attribute	Abbreviation	Comments
Relative proportion of collectors-gatherers	collgath	See comment to scrapers; optimum at around 20% collectors-gatherers (of all benthos), the maximum is 50% collectors-gatherers. They can never reach 100%. They are an indicator that a lot of detritus is introduced into the system. If there is substantially less than 20% this is also a bad sign and means that there is hardly any nutrition for this group, i.e. crushed or shredded material. The decreasing branch from 20 to 50% is rather typical and indicates that there is a diffuse input of material, while the increasing branch is untypical.
Relative proportion of filterers	filter	See comment to scrapers; optimum at around 20% filterers. This corresponds to the fourth order in the "river-continuum concept". Theoretically the proportion of filterers can reach 90 – 100%. In contrast to collectors and gatherers, the filterers are capable of feeding on diffuse input material. Nevertheless, high values of 90 – 100% are rather theoretical. Zero filterers are a special case if there is acidification and no fine material, and thus no nutrition. This is indicative of a diffuse load.
Relative proportion of periphyton	periph-BA	Theoretically, the relative proportion of periphyton concerns the production, but it is difficult to measure in practice. One would have to also measure the production of macrophytes and phytoplankton, which is difficult. The average value of periphyton is difficult to determine, and it strongly depends on the river type. However, there are a lot of good measurements available for this indicator. The shape is an optimum-curve. If there is few periphyton, this is an indicator of strong shading due to straightening of the river, or toxic substances in the water, or similar. If there is a lot of periphyton, there is no shading of the river, which indicates that there is a large distance from the river to the riparian vegetation. The optimum is around 50%.
Seasonally averaged density of periphyton	periph-BB	Benthos diversity depends strongly on the river type and can be different for different rivers. According to the river-continuum-concept, you would have more shredders, the higher up you are (i.e. in the wooden zones); the nearer you are to the river, the more filterers and periphyton you would have. But the Wigger is not fringed by riparian forest. BioB would need all the benthos feeding groups, but cannot give values; it is fine to use the value functions from BioA. Periphyton is measured as ash free dry biomass (AFDM)/ m^2; there is an optimum at around 100 g AFDM/ m^2 in summer; but BioB is not sure about the numbers.
Reti-index: (scrapers + wood-eaters + shredders)/ all feeding types	reti-index	The Reti-index was developed for smaller rivers and is appropriate for rivers up to the 4th order, i.e. such as the Wigger. It assesses the relative proportion of the most-important feeding groups for a Wigger-type river. It cannot increase to 100% because there are follow-organisms. The best-possible state is at around 40 – 50% of scapers, wood-eaters, and shredders. If there are fewer, there will be many filterers, and sediment loads or impacts of other substances. An improvement in the Reti-index always reflects a more-natural state of the river.
F13 Yoshimura-index: (scrapers + filterers)/ (shredders +	F13-index	The F13-index from Yoshimura et al. (2006) is also well suited to assess 4th order water bodies such as the Wigger. BioA calculated a few examples. Optimum (near-natural river): (30% shredders + 20% filterers)/ (20% shredders + 20% collectors-gatherers) = 1.25. Worst-case river: (5% scrapers + 10% filterers)/ (40% shredders + 30% gatherer-collectors) = 0.214. Another bad example with no riparian vegetation, and lots of fine sediments: (5% scrapers + 10% filterers)/ (0% shredders + 70% gatherer-collectors) = 0.214. A value of zero is hardly possible because this would mean that all feeding groups are reduced to zero. In reality, however, they would be replaced by other feeding groups. As the calculations demonstrate, to get a low value of the F13-index, different causes are possible. Therefore, BioA prefers an individual

Attribute	Abbreviation	Comments
gatherer-collectors)		assessment of the different feeding groups (which is required anyway to be able to calculate the index). This increases transparency and helps to evaluate the causes of a problem, while the causes are easily masked when using an index.
Natural diversity of macro-invertebrates	macroinvert	BioA would not use the diversity of macroinvertebrates as indicator; one would have to state exactly, which level one is assessing (species-level, genus-level?). Experience concerning diversity valuations is not so good; diversity can strongly fluctuate, is dependent of the season, amongst many other factors. Hence, even if diversity of macroinvertebrates is theoretically important, in practice it is not a very useful indicator.
Natural diversity of macro-invertebrates	macrinv-BP	BioPhys cannot give value functions for benthic organisms. One should use either the feeding groups or an index (not both). With respect to scrapers there is a certain overlap with primary production, which feeds the scrapers, but it is not the only thing that affects scrapers. BioPhys would prefer an assessment of the different feeding groups to an index. He gave a (linear) value function for the diversity of macroinvertebrates, for which he suggests using the Shannon Weaver index H', where S is the total number of species and p_i is the frequency of the ith species (probability that any given individual belongs to the species, hence p). $H' = -\sum_{i=1}^{S}(p_i \ln p_i)$
Natural diversity of macro-invertebrates	macrinv-BB	The diversity of the macroinvertebrates can substitute the other benthos feeding groups and can be used as attribute instead. The value function increases: the more diversity the better. It is possible to use any diversity index.
Mean density of ground beetles	grbeetl	BioA assessed ground beetles in his PhD; he found differences to other assessments, but these are mainly caused by more nutrient-rich or nutrient-poor rivers. However, this objective should not be used to assess the nutrient content of the river, but rather whether the shoreline is in a natural state. Ultimately, the more beetles, the better. There is also an extreme variance, depending on the season, the spot, etc. The relationship is not linear. It is a combination of nutrient richness, the proportion of near-natural shorelines, and aquatic-terrestrial interactions. If the latter alone were accessed, the relationship would be linear, but the nutrients cause an exponential value function. The maximum value is reached at about ten individuals per m^2 and stays there for up to 50 individuals/ m^2.
Mean density of rove beetles	rovbeetl	The density of rove beetles is highest when the flooding regime is (near-)natural. This equals around four individuals/ m^2; the more, the better. Variability is very high and depends on river type. Effects of morphology and habitat quality are not so strong. Shape of the value function is very similar to that of ground beetles.
Natural fish diversity, lower reaches Wigger	fish_low	
Total biomass of trout	totbiomasst	*Salmo trutta*. The value function is steeper if the river is in a bad state, because it is more important to improve the river if it is in a bad state than when it is already in a good state. The maximum in lower reaches is at around 250 kg/ ha.

Attribute	Abbreviation	Comments
Number of young of the year trout	YOYt	The young of the year are an indicator of natural reproduction. The value function is steeper when the river is in a bad state.
Number of juvenile trout	juvent	The juvenile trouts are the rest of the young trouts, i.e. without young of the year. Again, the value function is steeper when the river is in a bad state.
Total biomass of adult trout	adbiomasst	It is easier to assess the biomass of adults (kg/ ha) than to count individuals. The value function is steeper when the river is in a bad state.
Total biomass of adult barbel and/ or chub	adbiomass	*Barbus barbus*; *Leuciscus cephalus*. The value function is steeper if the river is in a bad state.
Number of young of the year barbel	YOYb	Barbel is a migrating species. Young barbel are very small and rather difficult to catch. Therefore, abundance is a difficult measure; they do not gather in swarms. Time of assessment is important. It should be autumn, e.g. September. The populations are not that large. Value function is steeper if the river is in a bad state.
Number of juvenile barbel and/ or chub	juvenb	The value function is steeper if the river is in a bad state.
Number of adult nase	adultn	*Chondrostoma nasus*. For the nase, the number of adult fish are counted at the spawning ground. If there are several spawning grounds, they are summed up, but typically there is only one. Today one would find around 100 adult nase in the Wigger. The increase from 0 to 300 individuals is very steep, because it is important to Fish to have some nase if there are none at the spawning ground. Hence, the first individuals are very valuable.
Occurrence of young of the year nase	YOYn	The young of the year nase are a very difficult attribute, and Fish thinks that there are no experts who could give a value function. The question is how to measure them. Additionally, in the Wigger they wander into the river Aare. However, if they do occur in the Wigger, it is very special. Therefore, the Fish-expert decided that two (yes/ no) classes for occurrence would best reflect this. One could, additionally, assess the occurrence in additional classes (sporadic, numerous), but the occurrence alone suffices.
Total biomass of spirlin	totbiomasssp	*Albumoides bipunctatus*. The Wigger has a rather low density of spirlin, typically, one does not find many. The juvenile spirlin were not assessed separately in the lower reaches, it is sufficient to have the total biomass of spirlin.
Dominance of any fish species	domin	In the Wigger it would be barbel (*Barbus barbus*) or chub (*Leuciscus cephalus*) that could dominate, both in the lower and the mid-reaches. In the lower reaches it could theoretically also be stoan loach (*Barbatula barbatula*), but this species does not have any weight compared to the other two species. If it were to be included, one would have to use another measure than kg/ ha.

Attribute	Abbreviation	Comments
Number of non-site-specific species	nonsite	Some fish can enter the lower reaches that do not really belong there. The values and range should be the same as in the middle reaches. The maximum would be around ten non site-specific species.
Percent fish with anomalies or injuries	anom	One would compare the percentage of fish with anomalies or injuries to all fish. The maximum of 50% is an extreme value that is hardly reached in reality (but is possible). There is a very strong decrease in value in the beginning, i.e. if one finds the first few fish with anomalies or injuries.
Natural fish diversity, mid reaches Wigger	fish_mid	
Total biomass of trout	totbiomasstm	*Salmo trutta*. Trout in mid-reaches is identical to head waters. It is still the dominant fish species. Additional indicators are barbel and a small fish species: stone loach.
Number of young of the year trout	YOYtm	Identical to head waters.
Number of juvenile trout	juventm	Identical to head waters.
Total biomass of adult trout	adbiomasstm	Identical to head waters.
Total biomass of barbel	totbiomassbm	*Barbus barbus*. If number of attributes is to be kept as small as possible, it suffices to assess total biomass of barbel. It is not necessary to assess biomass of adult barbel separately, since this is more or less the same. Rationale: total biomass of barbel is dominated by adult barbell and barbel are a migrating species.
Number of young of the year barbel	YOYbm	Barbel is a migrating species. The young barbels are very small and rather difficult to catch. Therefore, abundance is a difficult measure; they don't gather in swarms. The time of the measure is important. You would have to carry it out in autumn, for instance September. The populations are not that large.
Number of juvenile barbel	juvenbm	Without young of the year (YOY).
Biomass of stone loach	totbiomasslm	*Barbatula barbatula*. This is a site-specific species, but not a very sensitive indicator. However, Fish needs to know whether they occur or not. They should not be massively abundant.

Attribute	Abbreviation	Comments
Dominance of any fish species	dominm	Compared with trout, i.e. in the middle reaches of the Wigger, the dominating species would be barbel (*Barbus barbus*), or chub (*Leuciscus cephalus*). For this attribute it does not matter whether the dominating species is site-specific or not. Important is only that trout is not dominated by another species.
Number of non-site-specific species	nonsitem	To have ten non-site-specific species would be an extreme value.
Percent fish with anomalies or injuries	anomm	
Natural fish diversity, head water Wigger	fish_head	
Total bio-mass trout	totbiomassth	*Salmo trutta*. In head waters, the "total biomass of trout" is the same as "total biomass of fish".
Number of young of the year trout	YOYth	The young of the year are an indicator of natural reproduction. The value function is steeper when the river is in a bad state.
Number of juvenile trout	juventh	These are the remaining of the young trouts, i.e. without YOY.
Total bio-mass of adult trout	adbiomassth	It is easier to assess the biomass of adults (kg/ ha) than to count individuals.
Percent fish with anomalies or injuries	anomh	One would compare the percentage of fish with anomalies or injuries to all fish.

References

Langhans, S.D., Lienert, J., Schuwirth, N., Reichert, P., 2013. How to make river assessments comparable: A demonstration for hydromorphology. Ecological Indicators 32, 264-275.

LAWA, 2004. "Gewässerstrukturkartierung in der Bundesrepublik Deutschland - Übersichtsverfahren“; in German (Mapping the quality of water body structures in Germany - Overview procedures), Schwerin, Germany. ISBN 987-3-88961-249-6, http://www.lawa.de/, order number 300823, accessed 22.12.2014.

Schälchli, U., 2002. Innere Kolmation - Methoden zur Erkennung und Bewertung; in German (Inner substrate clogging - Methods for detection and assessment), Fischnetz-Publication (Project 01/11), Eawag, Dübendorf, Switzerland. http://www.fischnetz.ch/content_d/publ/tp.htm, accessed 22.12.2014.

Strager, M.P., Fletcher, J.J., Yuill, C.B., Strager, J.M., 2000. Not in My Watershed! An Interactive Tool to Evaluate Land Use Changes on Stream Water Quality, Prodeedings of the ESRI International User Conference. http://proceedings.esri.com/library/userconf/proc00/professional/papers/PAP663/p663.htm, accessed 22.12.2014.

USEPA, 2003. Developing water quality criteria for suspended and bedded sediments (SABS), Potential approaches, US EPA Office of Water, Office of Science and Technology, Draft, August 2003. http://water.epa.gov/scitech/swguidance/standards/criteria/aqlife/pollutants/sediment/upload/2004_08_17_criteria_sediment_sab-discussion-paper.pdf, accessed 22.12.2014.

van der Nat, D., Schmidt, A.P., Tockner, K., Edwards, P.J., Ward, J.V., 2002. Inundation dynamics in braided floodplains: Tagliamento River, Northeast Italy. Ecosystems 5, 636-647.

Yoshimura, C., Tockner, K., Omura, T., Moog, O., 2006. Species diversity and functional assessment of macroinvertebrate communities in Austrian rivers. Limnology 7, 63-74.
