# Supplementary material for: Four Common Simplifications of Multi-Criteria Decision Analysis do not hold for River Rehabilitation
Source: PLoS One. 2016 Mar 8;11(3):e0150695. doi: 10.1371/journal.pone.0150695 (PMC4783037; doi:10.1371/journal.pone.0150695)
Supplement: S4 Table — Certainty Equivalents (CE) elicited from the BioA-, BioB-, and BioPhys-expert. (PDF) [file pone.0150695.s011.pdf]

S4 Table. Risk attitudes. Points of utility functions = Certainty Equivalents (CE), compared to the 0.25, 0.5, and 0.75 points of the value functions (in the header row) elicited from three experts (BioA, BioB, and BioPhys).

Expert	Values	0.25	0.5	0.75
BioA		0.20	0.60	0.70
BioB		0.20	0.40	0.70
BioPhys		0.15	0.30	0.65
